# Mechanism of spindle stability and poleward flux regulating spindle length during the metaphase

**DOI:** 10.1016/j.isci.2025.113506

**Published:** 2025-09-04

**Authors:** Yao Wang, Yu-Ru Liu, Peng-Ye Wang, Hui Li, Ping Xie

**Affiliations:** 1 Key Laboratory of Soft Matter Physics, Institute of Physics, Chinese Academy of Sciences, Beijing 100190, China; 2 School of Physical Sciences, University of Chinese Academy of Sciences, Beijing 100049, China; 3 School of Systems Science and Institute of Nonequilibrium Systems, Beijing Normal University, Beijing 100875, China; 4 Key Laboratory of Cell Proliferation and Regulation Biology, Ministry of Education, Beijing Normal University, Beijing 100875, China

**Keywords:** Cell biology, Organizational aspects of cell biology, Bioinformatics

## Abstract

During metaphase, the spindle stabilizes chromosomes and maintains its size despite continuous microtubule poleward flux. To investigate the mechanism of the spindle stability and how the poleward flux regulates the spindle size, we establish a minimal spindle model that incorporates kinetochores, microtubules, spindle poles, and microtubule sliding proteins such as kinesin-5, microtubule depolymerizing proteins such as kinesin-13, and microtubule crosslinking proteins such as NuMA. We find that the poleward flux stabilizes the spindle by regulating the spindle length and the length of antiparallel microtubule overlaps to achieve equal rates of microtubule sliding, plus-end polymerization, and minus-end depolymerization. We reveal the underlying mechanism of how the poleward flux rate scales linearly with the spindle length and microtubule overlap length in small cells and how microtubule nucleation affects spindle dynamics in large cells.

## Highlights

• A minimal spindle model is proposed to study spindle dynamics during metaphase
• Shows how the spindle system maintains its stability
• Explains how flux rate scales linearly with spindle length and overlap length
• Explains how microtubule nucleation affects spindle dynamics in large cells

## Graphical abstract

**Figure:**
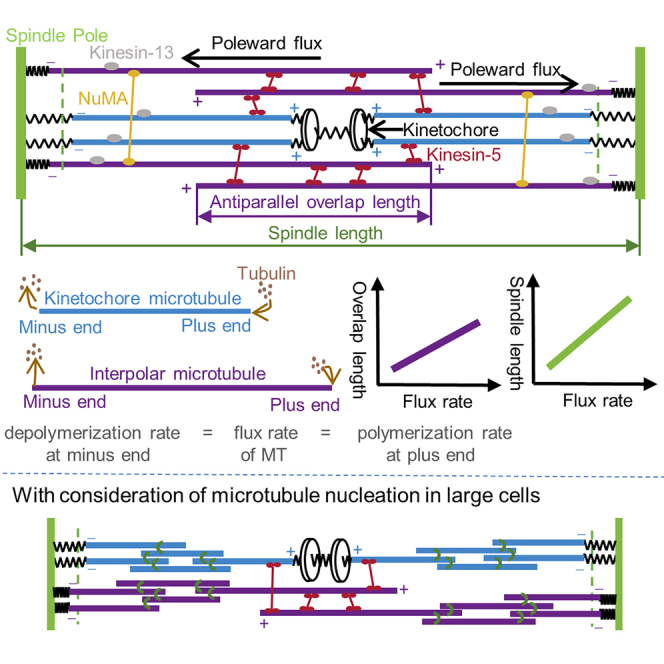

## Introduction

Cell division is mediated by a microtubule (MT)-based dynamic apparatus, known as the spindle, which ensures the alignment and segregation of chromosomes. During the metaphase of mitosis, the spindle maintains a bipolar structure with chromosomes situated at its center. Within the spindle, MTs can be categorized into three types based on their positions: kinetochore MTs (kMTs), interpolar MTs (iMTs) and astral MTs (aMTs). kMTs connect kinetochores to spindle poles, iMTs emanate from two spindle poles and form antiparallel overlaps in the midzone, and aMTs originate from the spindle poles and extend to the cell cortex. Most eukaryotic cells exhibit a spindle capable of maintaining a constant length while the iMTs and kMTs comprising the spindle continuously move toward the spindle poles.^1^ The dynamic process of MTs is termed as poleward flux.

Previous works suggested several roles for poleward flux during metaphase, such as assisting in the correct organization of MTs within the spindle and counteracting the extra nucleation of MTs in the spindle.^2^^,^^3^ It also helps in correcting erroneously connected MTs and kinetochores located at specific sites on the chromosome,^4^^,^^5^ facilitates the uniform distribution and association of kinetochores with kMTs,^6^^,^^7^ and aids in the segregation of chromosomes following kMTs to move toward the poles during late anaphase.^8^ However, whether the poleward flux has additional functions and how it operates during metaphase requires further analysis.

Mitotic cells require their spindles to maintain a constant length at metaphase to promote proper chromosome alignment and achieve faithful chromosome segregation. Experimental data showed that various factors can regulate spindle length, including molecular gradients,^9^^,^^10^ the density of kMTs,^11^ a balance of the spindle forming forces,^12^^,^^13^ and so forth. The observations of a correlation between flux rate and spindle length,^9^^,^^14^^,^^15^^,^^16^ concretely both spindle length and overlap length being proportional to the poleward flux rate,^6^^,^^17^^,^^18^^,^^19^ suggested that the poleward flux plays a key role in spindle length control.

Previous works have established models of the metaphase spindle to explain mechanisms regulating spindle length. Some studies have modeled motor proteins and/or MT cross-linking proteins that counteract each other in the antiparallel iMTs, investigating conditions for forming stable overlaps or spindle length, and proposing that the balance of pushing forces and pulling forces generated by proteins regulates the spindle length.^12^^,^^20^^,^^21^ Some studies have modeled the dynamics at the plus ends of MTs; for instance, the plus-end MT depolymerase kinesin-8s accumulate at the plus ends in a length-dependent manner, thereby limiting spindle length.^22^^,^^23^ The slide-and-cluster model proposed that the spindle length is determined by the sliding distance of MTs before it depolymerizes.^24^ It was also proposed that spindle poles can limit spindle length through forces generated by aMTs and through the mass conservation of tubulins.^25^ However, these models have not considered the MT poleward flux, rendering such spindle models unsuitable for cells such as mammals, where the poleward flux exists. To include the poleward flux, some models have been established to investigate the characteristics of the metaphase spindle, yet they have not considered the regulation on spindle length.^4^^,^^26^^,^^27^^,^^28^^,^^29^^,^^30^ Goshima et al. and Loughlin et al. have established metaphase spindle models considering the poleward flux and explained spindle length regulation.^31^^,^^32^^,^^33^ However, the detailed mechanism of the interaction between iMTs and kMTs and the coordination between polymerization and depolymerization to describe the dynamic process of the spindle and the direct correlation between flux rate and spindle length is still illusive.

In this article, we set out to identify how the poleward flux regulates the antiparallel overlap length and spindle length. We propose a spindle model encompassing kinetochores, kMTs, iMTs, spindle poles, and MT-associated proteins, including kinesin-5, kinesin-13, and nuclear mitotic apparatus protein (NuMA). Kinesin-5 is a homologous tetrameric bipolar protein, which can crosslink adjacent antiparallel MTs and drive them sliding apart.^14^^,^^34^ Kinesin-13 protein can bind to a single MT, and the MT sliding can deliver the MT-bound kinesin-13 to the minus end, where it performs the depolymerization activity.^35^^,^^36^ NuMA has two heads that are connected by a long (200-nm) stalk, which can crosslink two distant parallel iMTs, synchronizing the movement of multiple MTs.^6^^,^^37^ Our model deduces that the poleward flux aligns MT polymerization, sliding, and depolymerization by regulating overlap length and spindle length, thereby stabilizing the spindle.

## Results

In this work, we study numerically the spindle dynamics and compare our numerical results with the published experimental data for human cells of relatively small sizes^6^^,^^17^ and those for zebrafish embryo cells of large sizes.^38^ For ease of illustrating the mechanism of the spindle stability and for simplicity of analysis, in all sections except for the last section of Results, we firstly use the simplistic model that each kMT or iMT is a long MT spanning the centrosome-to-chromosome distance to make the study for the relatively small human cells. Then, in the last section of Results, we extend the simplistic model by considering the more realistic and more complicated case that the spindles are made of smaller MTs than the spindle size and the MT nucleation is present throughout the spindle to make the study for the large zebrafish cells.

### The minimal model for the spindle

**Figure 1:**
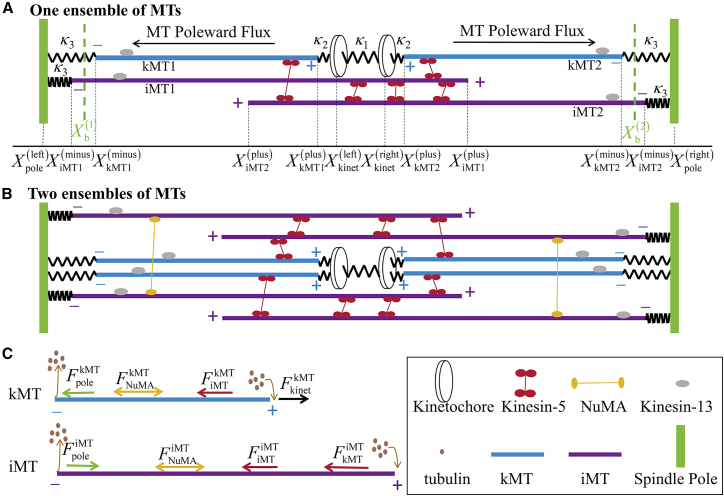
Schematic of the minimal model for the mitotic spindle (A) Spindle system composed of 1 ensemble of MTs (1 pair of kMTs and iMTs). (B) Spindle system composed of 2 ensembles of MTs (2 pairs of kMTs and iMTs). (C) Forces acting on the kMT and iMT, with arrows indicating the direction of the force exerted on the MT. $F_{\text{pole}}^{\text{kMT}}$ is the force on kMT resulting from the spindle pole, $F_{\text{pole}}^{\text{iMT}}$ is the force on iMT resulting from the spindle pole, $F_{\text{iMT}}^{\text{iMT}}$ is the force on iMT resulting from kinesin-5s in the iMT-iMT overlap, $F_{\text{kMT}}^{\text{iMT}}$ is the force on iMT resulting from kinesin-5s in the iMT-kMT overlap, $F_{\text{iMT}}^{\text{kMT}}$ is the force on kMT resulting from kinesin-5s in the iMT-kMT overlap, $F_{\text{kinet}}^{\text{kMT}}$ is the force on kMT resulting from the kinetochore, $F_{\text{NuMA}}^{\text{kMT}}$ is the force on kMT resulting from NuMAs and $F_{\text{NuMA}}^{\text{iMT}}$ is the force on iMT resulting from NuMAs.

We present a minimal model here. We exclude the role of aMTs and consider solely the role of iMTs and kMTs in the model, as generally done in the literature.^19^ We consider two cases. The first one is the simplest case that the spindle is composed of two kinetochores, two spindle poles, two kMTs and a pair of antiparallel iMTs (Figure 1A). This case is termed as 1 ensemble of MTs. There exist a spring of elastic constant $\kappa_{1}$ connecting two kinetochores,^4^^,^^39^ with an equilibrium distance of 1 μm,^6^^,^^39^^,^^40^ a spring of elastic constant $\kappa_{2}$ connecting the plus end of each kMT to the kinetochore and a spring of elastic constant $\kappa_{3}$ connecting the minus end of each MT to the spindle pole.^41^^,^^42^^,^^43^ The pair of iMTs form an antiparallel overlap near the plus end. Note that one iMT can also form an antiparallel overlap with one kMT.

The second one is the more complicated case in the spindle is composed of two kinetochores, two spindle poles, 2*N* kMTs and *N* pairs of antiparallel iMTs (*N* ≥ 2 is an integer). This case is termed as *N* ensembles of MTs. For example, for 2 ensembles of MTs, there are 4 kMTs and 2 pairs of antiparallel iMTs (Figure 1B). In the spindle, there exists one or more pairs of kinetochores, each of which may be connected to one or multiple kMTs.^29^^,^^42^^,^^44^ Our simulations show that, with given numbers of kMTs and iMTs, the variation of the number of kinetochore pairs does not affect our results for the spindle length, overlap length, and flux rate (see Figure S3). For simplicity, we use two kinetochores for our simulations here.

For 1 ensemble of MTs, only two types of proteins are considered. One type is kinesin-5 motors, and the other type is kinesin-13 motors. Since the homotetrameric kinesin-5 has a much larger probability of being located in the antiparallel-overlap region than in the parallel-overlap region,^45^ for simplicity, it is considered that kinesin-5 can only bind to MTs in the antiparallel overlap. Kinesin-13 can only bind to the MT regions where the antiparallel overlap is not formed. The models of kinesin-5 and kinesin-13 motors are presented in STAR Methods. For *N* ensembles of MTs, besides kinesin-5 and kinesin-13 motors, NuMA proteins^6^^,^^17^^,^^46^ are also considered, which crosslink two distant parallel iMTs, ensuring all iMTs slide at nearly the same rate. The model of NuMA is presented in STAR Methods.

Each MT can be polymerized at the plus end. The spontaneous polymerization and depolymerization, the polymerization promoted by polymerase enzymes^47^^,^^48^ as well as the depolymerization promoted by kinesin-8 and kinetochore-localized kinesin-13 motors,^17^^,^^22^^,^^46^^,^^49^^,^^50^^,^^51^^,^^52^ collectively result in the net polymerase activity at the plus end. We denote by *v*_p0_ the effective polymerization rate of iMTs. Since no force is present on the plus end of iMTs, the polymerization rate of iMTs is always *v*_p0_. Since the plus end of kMTs is connected to the kinetochore by linking proteins such as Ndc80, the linking proteins occupy some tubulins near the plus end of kMTs, and thus the number of the polymerase enzymes at the plus end of kMTs is smaller than at the plus end of iMTs.^10^^,^^41^ Moreover, the experimental data showed that the kinesin-13 proteins can localize at kinetochores, contributing to the kMT plus-end depolymerization.^17^^,^^51^^,^^52^ Hence, when no pulling force is present on the plus end of kMTs, the polymerization rate of kMTs should be smaller than that of iMTs, with the polymerization rate of kMTs being as $v_{p0}/B$, where *B* is a constant larger than one. When a pulling force $F_{\text{kinet}}^{\left(\text{kMT}\right)}$ is present on the plus end of kMTs, which arises from the stretching of the linkers connecting the linking proteins and the kinetochore, the polymerization rate of kMTs can be accelerated,^23^^,^^53^^,^^54^^,^^55^ which can be written as

(Equation 1) $$v_{\text{pol}}^{\left(\text{kMT}\right)}=\frac{v_{p0}}{B}\left(1+\frac{F_{\text{kinet}}^{\left(\text{kMT}\right)}}{F_{p0}}\right)\text{,}$$

where *F*_p0_ is the force-sensitivity parameter for the polymerization.

Here, we take *v*_p0_ as a preset value. In experiments, *v*_p0_ can be varied by the depletion of the plus-end tracking proteins, such as polymerase enzymes, kinesin-8 motors, etc.^6^^,^^16^^,^^17^^,^^18^^,^^30^^,^^46^^,^^47^ In order to compare quantitatively with the experimental data of Steblyanko et al.^17^ and Risteski et al.^6^ for human cells, unless otherwise pointed out, we take two sets of parameter values in our simulations, with one under the condition of Steblyanko et al.^17^ (called the First set of values) and the other one under the condition of Risteski et al.^6^ (called the Second set of values). The choice of the parameter values is described in STAR Methods.

### The stability of the spindle

In the main text, we mainly present the numerical results for 2 ensembles of MTs and some numerical results for 3 ensembles of MTs. The results for 1 ensemble of MTs are presented in supplemental information.

**Figure 2:**
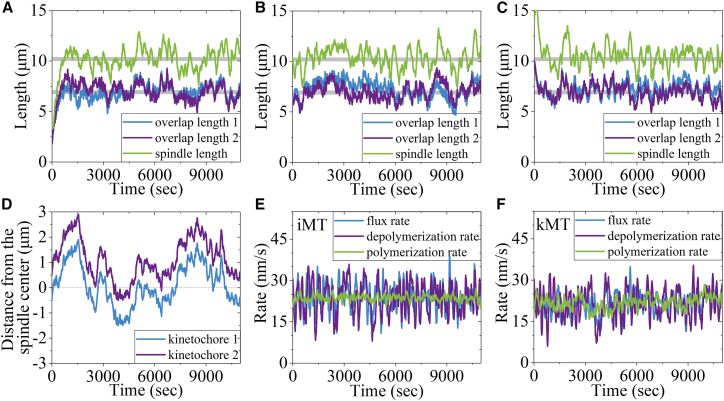
The stationary state of the spindle is independent of the initial condition in the simulations The simulations are done for 2 ensembles of MTs using the Second set of values for kinesin-5 motor given in Table S1, the Second set of values related to the polymerization of kMT from the plus end given in Table S4, and other parameter values given in Tables S2, S3, and S5, as well as *v*_p0_ = 24 nm/s. (A–C) Some examples for the temporal evolution of the overlap length and spindle length are simulated with different values of the initial overlap length and spindle length. The initial lengths (at *t* = 0) in (A) are evidently smaller than, those in (B) are close to, and those in (C) are evidently larger than the final stationary lengths. The thick gray lines indicate the same average overlap length of 6.92 μm and the same average spindle length of 10.2 μm for the three examples. The results show that different initial overlap lengths and spindle lengths give the same final stationary overlap length and spindle length. Moreover, different overlap regions have the same mean overlap length. (D) Temporal evolution of the positions of two sister kinetochores. The results show that the two sister kinetochores oscillate back and forth in the middle position of the spindle. (E and F) Temporal evolution of the flux rate, depolymerization rate, and polymerization rate for iMT and kMT. The results show that the flux rate, depolymerization rate, and polymerization rate for iMT have the same mean value, and those for kMT have the same mean value.

In this section, we study the spindle stability. To this end, we use different initial conditions to make the simulation. We use the preset value *v*_p0_ = 24 nm/s and the Second set of values to make the simulation. We note that taking different numbers and different distributions of kinesin-5, kinesin-13, and NuMA proteins on MTs has no effect on the final stationary poleward-flux rate, antiparallel-iMTs-overlap length (simply called overlap length), and spindle length (Video S1). We also show that by taking different initial overlap lengths and spindle lengths we always obtain the same final stationary mean overlap length and spindle length (Figures 2A–2C). These are consistent with the available experimental results showing that by perturbing the environment, the spindle can always return to its original stationary state.^56^ As expected, different pairs of antiparallel iMTs have the same mean overlap length (Figures 2A–2C). At the stationary state, the overlap length and spindle length fluctuate around the fixed values (Figures 2A–2C, Video S2). This feature is also consistent with that from the experimental results.^13^^,^^15^^,^^47^ In addition, we note that at the stationary state, the positions of the two kinetochores do not keep still but oscillate largely around the fixed values (Figure 2D), which is also consistent with the available experimental results^40^^,^^57^ (see discussion for details). Interestingly, at the stationary state, the flux rate of iMTs, polymerization rate of iMTs at the plus end and depolymerization rate of iMTs at the minus end have the same mean value (Figure 2E). So do the mean flux rate of kMTs, mean polymerization rate of kMTs at the plus end, and mean depolymerization rate of kMTs at the minus end (Figure 2F).

Video S1. The stationary state of the spindle is independent of the initial number of kinesin-5s and that of kinesin-13s The simulation results shown here are performed using *v*_p0_ = 24 nm/s and the Second set of values. For clarity, NuMAs are not shown here. Green dashed lines near spindle poles represent rest positions of spring linkers connecting MTs and poles. Pink blocks represent added tubulins at the MT plus end every 3 s.

Video S2. Temporal evolution of the spindle system The simulation results shown here are performed using *v*_p0_ = 24 nm/s and the Second set of values. For clarity, NuMAs are not shown here. Green dashed lines near spindle poles represent rest positions of spring linkers connecting MTs and poles. Pink blocks represent added tubulins at the MT plus end every 3 min.

The underlying mechanism that the flux rate of iMTs can be tuned automatically to a value equal to the polymerization rate of iMTs at the plus end can be explained as follows. Consider a given polymerization rate of iMTs at the plus end, *v*_p0_. If the initial overlap length *L*_i_ is smaller than the overlap length *L*_si_ at the stationary state, the number of kinesin-5 motors bound to the overlap of length *L*_i_ would be smaller than that bound to the overlap of length *L*_si_ (see Figure S4), resulting in that each kinesin-5 motor experiences a larger resistance force *F*_m_ for a constant pushing force on the minus ends of the two iMTs. The larger *F*_m_ induces the iMT-sliding rate *v*_slide_ to be smaller than *v*_p0_. This would result in the increase of the overlap length and thus the increase of the number of kinesin-5 motors bound to the overlap, which decreases the resistance force *F*_m_ on each motor and thus increases the iMT-sliding rate *v*_slide_ until it becomes equal to *v*_p0_ and the overlap length becomes equal to *L*_si_. Similarly, if the initial overlap length *L*_i_ is larger than the stable *L*_si_, giving the initial iMT-sliding rate *v*_slide_ to be larger than *v*_p0_, *v*_slide_ would decrease until it becomes equal to *v*_p0,_ and the overlap length would decrease until it becomes equal to *L*_si_.

The underlying mechanism that the depolymerization rate at the iMT minus end can be tuned to a value equal to the iMT-sliding rate *v*_slide_ can be explained as follows. Denote by L the length of the non-overlapping region on an iMT, where kinesin-13 motors can bind. The depolymerization rate at the minus end, *v*_dep_, increases with *L* (see Methods S8 and Figure S6 in supplemental information, where we show that kinesin-13 motors depolymerize MTs from the minus end in a length-dependent manner). Considering, e.g., the initial length *L* is smaller than the length at the stationary state, *L*_s_, the depolymerization rate *v*_dep_ is smaller than the MT sliding rate *v*_slide,_ and thus the non-overlapping length would increase until reaching *L*_s_. If the depolymerization rate becomes larger than *v*_slide_, the length *L* would decrease, resulting in a decrease in the depolymerization rate. Thus, at the stationary state, the depolymerization rate of iMTs at the minus end would fluctuate around the value equal to *v*_slide_ = *v*_p0_.

The underlying mechanism by which the depolymerization rate of kMT at the minus end can be tuned to a value equal to the polymerization rate of kMT at the plus end can be explained as follows. Consider that at one moment, the polymerization rate of kMT at the plus end is smaller than the depolymerization rate of kMT at the minus end. The length of kMT would decrease, leading to an increase in the distance between the kinetochore and the plus end of kMT and thus the increase of the pulling force $F_{\text{kinet}}^{\left(\text{kMT}\right)}$ on both the plus and minus ends of the kMT. As a result, the kMT polymerization rate at the plus end would increase [see Equation 1] and the kMT depolymerization rate at the minus end would decrease due to the decrease of the non-overlapping length *L* until the two rates become equal to each other. Similarly, if the polymerization rate is larger than the depolymerization rate, the kMT polymerization rate would decrease and the kMT depolymerization rate would increase due to the increase of *L* until the two rates become equal to each other. Therefore, at the stationary state, the depolymerization rate would fluctuate around the value equal to the polymerization rate.

**Figure 3:**
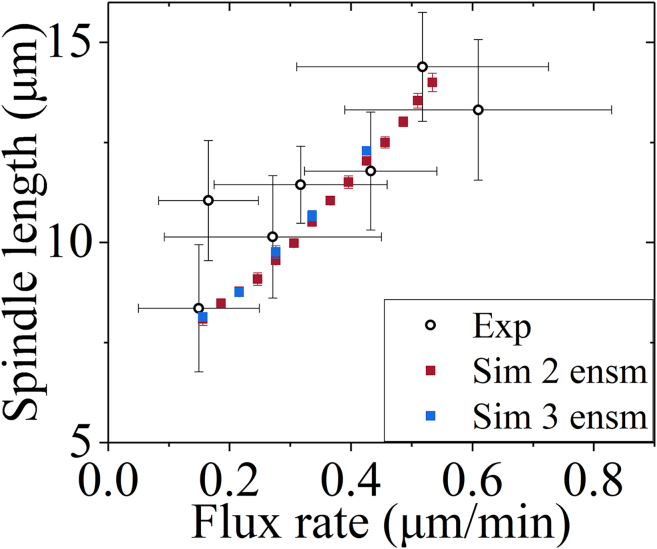
The spindle length increases with the increase in MT flux rate Filled squares represent the simulation data (mean ± SEM), which are computed for 2 and 3 ensembles of MTs using the First set of values for kinesin-5 motor given in Table S1, the First set of values related to the polymerization of kMT from the plus end given in Table S4 and other parameter values given in Tables S2, S3, and S5. Black hollow circles represent experimental data of Steblyanko et al.^17^ The spindle length is defined as the average distance between the two spindle poles after the spindle reaching the stationary state. The flux rate is defined as the average value of the 2*N* (*N* = 2 and 3) iMT-sliding and 2*N* kMT-sliding rates after the spindle reaches the stationary state, with $v_{\text{flux}}=\left(\sum_{m=1}^{2N}v_{m}^{\left(\text{iMT}\right)}+\sum_{n=1}^{2N}v_{n}^{\left(\text{kMT}\right)}\right)/\left(4N\right)$ (m≤2N, n≤2N), where *v*_flux_ is the flux rate, $v_{m}^{\left(\text{iMT}\right)}$ is the *m*th iMT-sliding rate and $v_{n}^{\left(\text{kMT}\right)}$ is the *n*th kMT-sliding rate. *N* = 2 and 3 correspond to 2 and 3 ensembles of MTs, respectively.

**Figure 4:**
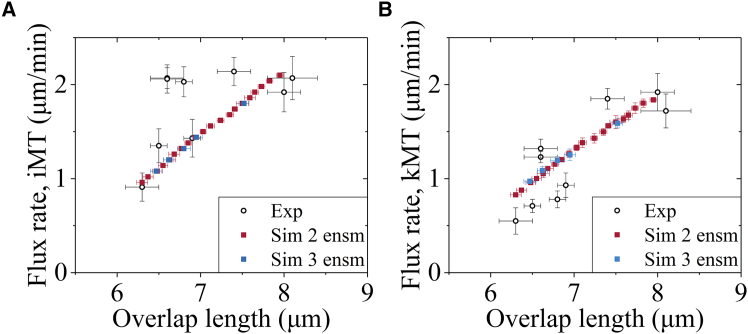
Flux rates of iMTs and kMTs increase with the increase of the overlap length Filled squares represent the simulation data (mean ± SEM), which are computed for 2 and 3 ensembles of MTs using the Second set of values for kinesin-5 motor given in Table S1, the Second set of values related to the polymerization of kMT from the plus end given in Table S4 and other parameter values given in Tables S2, S3, and S5. The black hollow circles represent experimental data of Risteski et al.^6^ The flux rate of iMTs and that of kMTs are defined as the average value of the sliding rates of 2*N* iMT and that of the sliding rates of 2*N* kMTs, respectively, and the overlap length is the average value of the *N* overlap lengths after the spindle reaches the stationary state. (A) Flux rate of iMTs versus overlap length. (B) Flux rate of kMTs versus overlap length.

It was shown before that kinesin-13 can only diffuse unidirectionally and cannot move directionally on MT lattices.^49^^,^^58^ As a result, it was proposed before that kinesin-13 makes use of the unbiased-diffusion mechanism to reach both the plus and minus ends with the equal probability and thus depolymerizes MTs from both ends with nearly the same rate.^36^^,^^49^ However, it is generally believed that during the metaphase, the depolymerization of iMTs and kMTs from the minus end was mainly performed by kinesin-13 motors, while the depolymerization of iMTs from the plus end was mainly performed by kinesin-8 motors, and the depolymerization of kMTs from the plus end was performed by both the kinesin-8 motors and kinetochore-localized kinesin-13 motors. Here, we note that kinesin-13 motor makes mainly use of the poleward sliding of the iMTs and kMTs to be delivered to the minus end (Figures S6G–S6I). Thus, it is understandable that why kinesin-13 does not have the ability to move directionally toward the minus end. Since the movement velocity of the kinesin-8 KIF18B motor on MTs, which is about 350 nm/s *in vitro* and 700 nm/s *in vivo*,^49^ is much larger than the poleward-sliding velocity of iMTs and kMTs during the metaphase, which is usually smaller than about 34 nm/s^6^^,^^15^^,^^42^ (see Figures 3 and 4), the kinesin-8 KIF18B motors can make a net movement toward the plus end of iMTs and kMTs, where they perform the depolymerase activity.^22^^,^^49^

### The flux rate determines the overlap length and spindle length

In this section, we study the effects of the flux rate on the overlap length and spindle length, comparing the numerical results with the available experimental data for human cells.^6^^,^^17^ To this end, we vary the value of the preset parameter *v*_p0_, as mentioned above.

First, we use the First set of values. The numerical results of the spindle length versus the flux rate for 2 ensembles of MTs and those for 3 ensembles of MTs, which are consistent with each other, show a strong positive correlation between the flux rate and spindle length (Figure 3). The numerical results are in good agreement with the experimental data of Steblyanko et al.^17^ The results for 1 ensemble of MTs are shown in Figure S7 (see supplemental information). By comparing Figure 3 with Figure S7, it is seen that at a given flux rate, the spindle length for 1 ensemble of MTs is smaller than that for *N* ensembles of MTs (*N* = 2 or 3). This is because for *N* ensembles of MTs, the presence of NuMA affects the sliding of iMT driven by kinesin-5, whereas for 1 ensemble of MTs, no NuMA is present. For 1 ensemble of MTs, by adjusting only one parameter *F*_p0_ while with other parameter values being kept unchanged, our numerical results agree with the experimental data (Figure S7).

Second, we use the Second set of values. The numerical results of the flux rates of iMTs and kMTs versus the overlap length for 2 ensembles of MTs and those for 3 ensembles of MTs, which are consistent with each other, reproduce quantitatively the experimental data of Risteski et al.^6^ (Figure 4). Some results for the temporal evolution of the overlap length and spindle length at the stationary state for 3 ensembles of MTs are shown in Figure S8 (see supplemental information). The results for 1 ensemble of MTs are shown in Figure S9 (see supplemental information). For 1 ensemble of MTs, by adjusting only one parameter *F*_p0_ while with other parameter values being kept unchanged, our numerical results agree with the experimental data (Figure S9).

### Effects of perturbations of parameters on spindle dynamics

Since the depolymerization of iMTs and kMTs at the minus end and the polymerization at the plus end play important roles in the spindle dynamics, it is interesting to study the effect of varying the value of each parameter related to the two activities on the spindle dynamics. We use the preset value *v*_p0_ = 24 nm/s and the Second set of values to make the simulation. As noted above, the iMT flux rate is equal to *v*_p0_, independent of the variation of other parameters.

**Figure 5:**
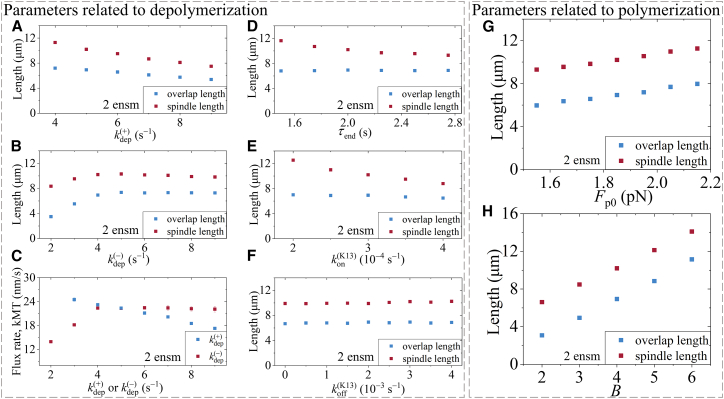
Parameter sensitivity analysis at the stationary state of the spindle The simulations are done for 2 ensembles of MTs using the Second set of values for kinesin-5 motor given in Table S1, the Second set of values related to the polymerization of kMT from the plus end given in Table S4, and other parameter values given in Tables S2, S3, and S5, as well as *v*_p0_ = 24 nm/s. (A–F) Effects of the variation of each parameter associated with kinesin-13 at the minus end and on the MT lattice on overlap length, spindle length, and kMT flux rate. (G and H) Effects of the variation of each parameter associated with the MT polymerization at the plus end on overlap length and spindle length.

First, we investigate how the variation of the parameters related to kinesin-13 at the minus end ($k_{\text{dep}}^{\left(+\right)}$, $k_{\text{dep}}^{\left(-\right)}$ and $\tau_{\text{end}}$) and those related to kinesin-13 on the MT lattice ($k_{\text{on}}^{\left(K13\right)}$ and $k_{\text{off}}^{\left(K13\right)}$) affect the spindle dynamics. Here, $k_{\text{dep}}^{\left(+\right)}$ and $k_{\text{dep}}^{\left(-\right)}$ are depolymeriztion rates of the kinesin-13 motor at the minus end under the pushing and pulling forces, respectively, on the minus end, $\tau_{\text{end}}$ is the MT-end residence time of the kinesin-13 motor, $k_{\text{on}}^{\left(K13\right)}$ is the binding rate of kinesin-13 to MT, and $k_{\text{off}}^{\left(K13\right)}$ is the detachment rate of kinesin-13 from MT. We note that an increase in $k_{\text{dep}}^{\left(+\right)}$ results in the decrease in the overlap length, spindle length, and kMT flux rate (Figures 5A and 5C). By comparison, when $k_{\text{dep}}^{\left(-\right)}$ < $k_{\text{dep}}^{\left(+\right)}$ (5 s^−1^) an increase in $k_{\text{dep}}^{\left(-\right)}$ leads to the increase in the overlap length, spindle length, and kMT flux rate, whereas when $k_{\text{dep}}^{\left(-\right)}$ > $k_{\text{dep}}^{\left(+\right)}$ (5 s^−1^) the increase in $k_{\text{dep}}^{\left(-\right)}$ has a slight effect on the overlap length, spindle length, and kMT flux rate (Figures 5B and 5C). The MT-end residence time, $\tau_{\text{end}}$, only affects the spindle length but does not affect the overlap length (Figure 5D). The parameters of kinesin-13 on the MT lattice, $k_{\text{on}}^{\left(K13\right)}$ and $k_{\text{off}}^{\left(K13\right)}$, do not affect the overlap length (Figures 5E and 5F). However, the increase in $k_{\text{on}}^{\left(K13\right)}$ results in the reduction in the spindle length, making the spindle length be closer to the overlap length (Figure 5E). Since $k_{\text{on}}^{\left(K13\right)}$ is positively correlated with kinesin-13 concentration (see STAR Methods), the results of Figure 5E thus imply that the kinesin-13 concentration exhibits a negative correlation with the spindle length, which is consistent with experimental data.^59^ Varying the detachment rate $k_{\text{off}}^{\left(K13\right)}$ in the range from 0 to 0.004 s^−1^ has a little effect on the spindle length and overlap length (Figure 5F). It is noted that an upper limit is present, above which the depolymerization rate becomes not dependent on the MT length, thus preventing the spindle from achieving equilibrium. Additionally, it is noted that $\tau_{\text{end}}$, $k_{\text{on}}^{\left(K13\right)}$ and $k_{\text{off}}^{\left(K13\right)}$ have a little impact on the kMT flux rate (data not shown). For 1 ensemble of MTs, the effects of $k_{\text{dep}}^{\left(+\right)}$, $k_{\text{dep}}^{\left(-\right)}$, $\tau_{\text{end}}$, $k_{\text{on}}^{\left(K13\right)}$ and $k_{\text{off}}^{\left(K13\right)}$ are similar (Figures S10A–S10F).

Then, we investigate how the variation of the parameters related to the polymerization at the plus ends (*F*_p0_ and *B*, defined above) affect the dynamics of the spindle. We find that the overlap length is proportional to both *F*_p0_ and *B*, and the increase in spindle length mirrors the change in the overlap length (Figures 5G and 5H). As mentioned above, the kinetochore-localized kinesin-13 motors contribute to the kMT plus-end depolymerization, equivalent to the reduction of the effective polymerization rate at the plus end of kMT, which is characterized by the variation of *B*. Thus, the results about the effect of *B* on the spindle length (Figure 5H) indicate that the kinetochore-localized kinesin-13 motors can regulate the spindle length, which is consistent with the published experimental data.^17^^,^^51^^,^^52^ *F*_p0_ and *B* have a little impact on the kMT flux rate (data not shown). For 1 ensemble of MTs, the effects of *F*_p0_ and *B* are similar (Figures S10G and S10H).

We also investigate the effects of the three spring elastic constants $\kappa_{1}$, $\kappa_{2}$ and $\kappa_{3}$ on the spindle dynamics (see Methods S9, Figures S11 and S12 in supplemental information). We find that in the wide range of values for each elastic constant, both the overlap length and spindle length change slightly, implying that the overlap length and spindle length are insensitive to the spring elastic constants (Figures S11 and S12).

We also investigate the effect of the variation of parameters related to NuMA, the binding rate of NuMA in the region of parallel iMTs, $k_{\text{on}}^{\left(\text{NuMA}\right)}$, and the elastic constant of the NuMA stalk, $K_{\text{NuMA}}$, on the spindle dynamics (see Figure S13). We find that the variation of $k_{\text{on}}^{\left(\text{NuMA}\right)}$ or $K_{\text{NuMA}}$ has a little effect on the overlap length and spindle length (Figures S13A and S13B), because the variation of $k_{\text{on}}^{\left(\text{NuMA}\right)}$ or $K_{\text{NuMA}}$ has a little effect on the mean total force on an iMT along the plus-end direction, which arises from the stretching of all NuMA stalks, and that along the minus-end direction (Figures S13C and S13D).

As noted above, with the unloaded velocity of kinesin-5 being larger than *v*_p0_/2, the length of the antiparallel overlap and thus the number of kinesin-5s in the overlap can be automatically tuned to give the iMT-sliding rate *v*_slide_ to be equal to *v*_p0_ under the constant pushing force on the iMT minus end. The velocity of kinesin-5 is determined mainly by *k*^(+)^, *k*_r_ and *E*_D_, where *k*^(+)^ is the rate of ATP transition to ADP in the trailing head, *k*_r_ is the rate of the tail domain releasing from the nucleotide-free head and *E*_D_ is the energy change associated with the conformational change of the head and NL docking induced by ATP binding. Under a given overlap length, the number of kinesin-5s in the antiparallel overlap is determined by the parameters related to the binding and unbinding of kinesin-5. $k_{\text{on}}^{\left(m\right)}$, $\varepsilon_{w0}$, $\delta_{w}$, $\varepsilon_{s0}$ and $\delta_{s}$. Here, $k_{\text{on}}^{\left(m\right)}$ is the MT-binding rate, $\varepsilon_{w0}$ and $\varepsilon_{s0}$ are unloaded dissociation rates in weak- and strong-MT binding states, respectively, and $\delta_{w}$ and $\delta_{s}$ are load-sensitivity distances for the dissociation in weak- and strong-MT binding states, respectively. Thus, it is interesting to investigate the effects of *k*^(+)^, *k*_r_, *E*_D_, $k_{\text{on}}^{\left(m\right)}$, $\varepsilon_{w0}$, $\varepsilon_{s0}$, $\delta_{w}$ and $\delta_{s}$ on the overlap length and spindle length (see Figure S14). The increase of either *k*^(+)^ or *k*_r_, giving the increase of the unloaded velocity, increases the overlap length and spindle length (Figures S14A and S14B). The increase of *E*_D_, giving the increase of the loaded velocity, increases the overlap length and spindle length (Figure S14C). The increase of $k_{\text{on}}^{\left(m\right)}$, giving the increase of kinesin-5 number in the antiparallel overlap, decreases the overlap length and spindle length (Figure S14D). The increase of $\varepsilon_{w0}$, $\varepsilon_{s0}$ or $\delta_{s}$, giving the decrease of kinesin-5 number in the antiparallel overlap, increases the overlap length and spindle length (Figures S14E, S14F, and S14H). The change of $\delta_{w}$ has a little effect on the overlap length and spindle length (Figure S14G), because kinesin-5 dissociation in the weak MT-binding state is insensitive to the backward load^34^^,^^60^ and thus the change of $\delta_{w}$ has a little effect on the dissociation.

### Dynamics of the spindle system with the consideration of microtubule nucleation in large cells

In large embryos, such as zebrafish and Xenopus egg extract, branching MT nucleation is a critical phenomenon in spindle assembly, with MTs being shorter than the distance from the poles to the spindle center.^3^^,^^38^ We designate MTs directly connected to kinetochores as “stem” kMTs and those near the spindle center forming antiparallel overlaps as “stem” iMTs. Between the stem kMTs (or iMTs) and the spindle poles, smaller MTs are distributed. Multiple smaller MTs interconnect to facilitate connections between the poles and kinetochores and connections between the poles and central overlap, which collectively contribute to the structural integrity of the spindle.

**Figure 6:**
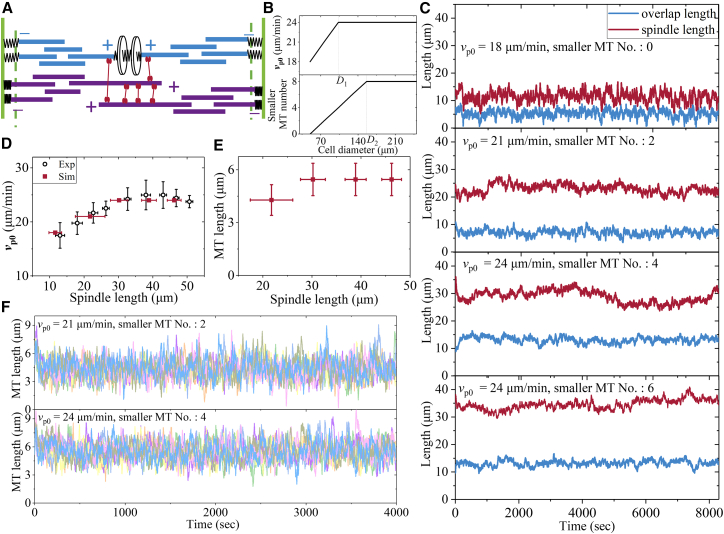
Results for the dynamics of the spindle system made of multiple smaller MTs in zebrafish cells The numerical results are computed using parameter values for the kinesin-5 motor given in Table S6, parameter values for the kinesin-13 motor given in Table S7, the Second set of values related to the polymerization of kMT from the plus end given in Table S4, and parameter values for spring elastic constants given in Table S5. (A) Schematic of the spindle system with the consideration of MT nucleation. (B) Effective polymerization rate *v*_p0_ correlates with cell size when the cell diameter *D* is smaller than *D*_1,_ but remains constant when *D* is larger than *D*_1_. Smaller MT number correlates with cell size when *D* is smaller than *D*_2_, but remains constant when *D* is larger than *D*_2_. Smaller MT number (No.) represents the number of smaller MTs (except for the stem kMT or iMT) in each iMT or kMT. (C) Temporal evolution of the overlap length and spindle length simulated with different numbers of smaller MTs. (D) Effective polymerization rate *v*_p0_ scales linearly with spindle length (mean ± SD) in smaller cells and remains constant in larger cells. The experimental data are taken from Rieckhoff et al.^38^ (E) Average length of smaller MTs versus spindle length (mean ± SD). (F) Length of smaller MTs, with different colors representing different MTs.

For this more realistic and more complicated case in large cells, we extend the above simplistic model by considering that the spindles are made of smaller MTs than the spindle size, and MT nucleation is present throughout the spindle (Figure 6A). In more detail, each long “kMT” is made of many smaller kMTs besides the stem kMT, and all kMTs are connected rigidly with each other via proteins called nucleation proteins located in the parallel overlap regions. Similarly, each long “iMT” is made of many smaller iMTs besides the stem iMT and all iMTs are connected rigidly with each other via nucleation proteins located in the parallel overlap regions. Except for the stem kMT connected to the kinetochore, whose plus end is not free, each kMT or iMT grows independently with velocity *v*_p0_ from its free plus end, as in the above simplistic model. The stem kMT grows with velocity $v_{\text{pol}}^{\left(\text{kMT}\right)}$ from its plus end, which is still described by Equation 1. Because all iMT are connected with each other, they move concertedly in the minus-end direction driven by kinesin-5 motors in the antiparallel overlap of the stem iMTs. Similarly, because all kMTs are connected with each other, they also move concertedly in the minus-end direction. The minus-end movement of each kMT or iMT transports kinesin-13 motors bound to it to its minus end, where they depolymerize the MT in a length-dependent manner, as shown above (noting that the depolymerization activity in one smaller MT is independent of that in another smaller MT). Furthermore, for the large cells, the saturation of the MT growth rate must be considered. Based on the experimental data showing that *v*_p0_ increases with the increase of the cell diameter (*D*) when *D* is small and becomes saturated when *D* is large (*D* > *D*_1_),^38^ we assume the simple relationship between *v*_p0_ and *D* as shown in Figure 6B (upper panel). Based on the experimental data showing that the MT number is proportional to the cell volume when *D* is small (*D* < *D*_2_, with *D*_2_ > *D*_1_) and becomes saturation when *D* > *D*_2_,^38^ we assume the simple relationship between the MT number and *D* as shown in Figure 6B (lower panel).

With this more complicated model and the method described in Methods S6 (see supplemental information), we can also obtain the stable spindle system for any *v*_p0_, with the average length of each MT, antiparallel MT overlap length, and spindle length being independent of the initial condition, similar to the case with the simplistic model. The spindle length firstly increases with *v*_p0_ and still increases but with *v*_p0_ being kept unchanged (Figures 6C and 6D). Each smaller MT has a stable length, with the length also increasing firstly with the increase of the spindle length and then becoming unchanged with the further increase of the spindle length (Figures 6E and 6F). All these numerical results are in good agreement with the experimental data measured by Rieckhoff et al. for large zebrafish cells.^38^ For clarity, we provide three short video segments from our simulations for the spindle systems made of smaller MTs of different numbers (see Video S3, noting that the length scales in the three video segments are different).

Video S3. Temporal evolution of the spindle system with branching nucleation The simulation results shown here are performed using *v*_p0_ = 24 μm/min. Green dashed lines near spindle poles represent rest positions of spring linkers connecting MTs and poles. Pink blocks represent added tubulins at the MT plus end.

## Discussion

In our previous work,^61^ we presented a minimal model for spindle elongation during anaphase, where, besides MTs and spindle poles, only two important participants—kinesin-5 motors and passive PRC1 proteins that can crosslink the two neighboring antiparallel MTs—are considered. Using the model, we explained the mechanism of how PRC1 can regulate the spindle elongation in a dose-dependent manner. In this work, we present a minimal model for spindle stability during metaphase, where, besides MTs, kinetochores, and spindle poles, only three important participants—kinesin-5 motors, kinesin-13 motors, and NuMA proteins—are considered. For 1 ensemble of MTs, only two important participants—kinesin-5 and kinesin-13 motors—are required. Using the model, we explain the mechanism of how the spindle can maintain the stationary state during metaphase and how the flux rate can regulate the overlap length and spindle length. In the model, the spindle dynamics are dictated mainly by three independent activities—the polymerization activity at the plus end, the MT-sliding activity performed by the kinesin-5 motors, and the depolymerization activity at the minus end performed by the kinesin-13 motors. The polymerization velocity *v*_p0_ at the free plus end of iMTs or smaller kMTs is taken as a preset value, and the effects of other two parameters related to the polymerization activity at the plus end of stem kMTs on the spindle dynamics are explored (Figures 5G and 5H). The variations of parameter values for the kinesin-5 motor can reproduce quantitatively the experimental data measured by three research groups which showed very large differences in the flux rate or *v*_p0_^6^^,^^17^^,^^38^ (Figures 3, 4, and 6). The effects of parameters for the kinesin-13 motor on the spindle dynamics are explored (Figures 5A–5F).

Prior models assumed that the depolymerization rate of MTs at the minus end depends sensitively on the force on the MT^26^^,^^31^ or on the distance between the MT minus end and the spindle pole.^30^^,^^61^ If the assumption (called assumption 1) that the variation of the depolymerization rate arises solely from the variation of the force on the MT is correct, since a pushing force is on the minus end of iMTs whereas a pulling force is on kMTs, in order to maintain the depolymerization rate of iMTs (kMTs) at the minus end equal to the sliding rate of iMTs (kMTs) it is required that both the pushing and pulling forces increase the depolymerization rate. This requirement seems unreasonable because the pushing force induces the tubulins near the end to be more curved, while the pulling force induces the tubulins near the end to be straighter. Even if the argument that both the pushing and pulling forces increase the depolymerization rate is correct, we have checked that with assumption 1, for a given polymerization rate of iMTs at the plus end, *v*_p0_, by adjusting the form of the depolymerization rate at the minus end versus the force for iMTs and that for kMTs we can get an stationary overlap length and spindle length, but changing *v*_p0_ slightly to another value and with the above-determined forms we cannot obtain the stationary overlap length and spindle length. Thus, with assumption 1, the experimental results showing that the flux rate regulates the overlap length and spindle length cannot be explained. Because the distance between the minus end and spindle pole is also determined by the force on the MT, the assumption that the variation of the depolymerization rate at the minus end arises from the variation of the distance between the minus end and spindle pole (called assumption 2) would have a similar effect on the dynamics of the spindle to assumption 1. Thus, with assumption 2, the experimental results showing that flux rate determines the overlap length and spindle length also cannot be explained.

Our model proposes that the depolymerization at the minus end is performed by kinesin-13 depolymerases at the minus end, which is supported by the available experimental results. For instance, the depolymerization rate of the minus end was found to be one hundred times faster in the presence of depolymerases compared to their absence.^62^ Spindles in human mitotic cells depleted of kinesin-13 depolymerases Kif2A and MCAK exhibit a lack of detectable flux.^5^ In our model, kinesin-13s bind to MTs, and thus the depolymerization rate is correlated with the MT length.

In our model, the polymerization activity, the depolymerization activity of the kinesin-13 motors, and the binding, unbinding, and stepping activities of the Eg5 motors occur stochastically. These stochastic activities collectively induce irregular fluctuations of the flux rate and polymerization/depolymerization rates (Figures 2E and 2F), in turn inducing spontaneously the large-scale oscillation of the kinetochore position (Figure 2D). Our results showed that the oscillation amplitude increases with the increase of *v*_p0_ (Figure S15). Since the depletion of kinesin-8 motors reduces the depolymerization rate at the plus end, equivalent to the enhancement of *v*_p0_, the above results (Figure S15) thus imply that the kinesin-8 depletion enhances the oscillation amplitude, which is consistent with the experimental evidence.^27^^,^^63^^,^^64^ Note that from Figure 2D, we see that the kinetochores oscillate with a timescale of about 3000 s, which is about 7.5-fold larger than about 400 s observed experimentally.^65^ We have checked that decreasing $\kappa_{1}$ can make the oscillation timescale be consistent with the experimental one. For example, taking $\kappa_{1}$ = 0.04 pN/nm, the oscillation timescale is about 450 s (Figure S16), which is close to the experimental one.^65^ This is because weakening the coupling between the two kinetochores enhances the asynchronous motion of the two kinetochores, resulting in a faster change of the kinetochore motion direction. Note that changing $\kappa_{1}$ has little effect on other spindle dynamics such as overlap length, spindle length, and flux rate (see Figure S11A). Note that the origin of the kinetochore oscillation in our model is different from those proposed in the literature, where it was generally believed that the oscillation is due mainly to the transitions between rescue and catastrophe phases at the plus end of kMTs.^28^^,^^29^^,^^66^ The detailed analyses of how the rapid and irregular fluctuations of the flux rate and polymerization/depolymerization rates (Figures 2E and 2F) can induce the slow and large-scale oscillation of the kinetochore position (Figure 2D; Figures S15 and S16) as well as the role of the oscillation in the elimination of erroneous MT-kinetochore attachment will be analyzed and studied in the future.

Our model takes into account the cross-linking role of NuMA on distant parallel iMTs. This makes all iMTs slide with nearly the same rate, ensuring the stability of the system. Due to the poleward flux, the number of NuMAs per μm in the overlap of two parallel iMTs increases as the distance to the spindle pole decreases (see Figure S17). If no NuMA is present, in the system with multiple ensembles of MTs, different iMT pairs can have very different sliding rates, which can result in the overlap length of one iMT pairs becomes smaller and smaller while the overlap length of another iMT pair becomes larger and larger, the system becoming unstable (see Figure S18). For simplicity, we do not consider NuMA cross-linking kMTs and cross-linking iMT and kMT. In addition, the model does not consider other roles played by NuMA within the spindle, such as its ability to enrich depolymerases Kif2A and affect depolymerization efficiency.^67^ There are many proteins involved in driving or resisting MT sliding, providing pushing or pulling force other than kinesin-5, such as kinesin-12, passive crosslinker, etc.^6^^,^^17^^,^^68^ The future studies are expected to include these aspects.

In summary, we explore the stabilization mechanism of the metaphase spindle in the presence of the MT poleward flux. The poleward flux stabilizes the spindle through the regulation of both spindle length and the antiparallel overlap length, thereby achieving equal rates of MT plus-end polymerization, sliding, and minus-end depolymerization. We find that the polymerization rate at the free iMT plus end determines the iMT and kMT flux rates. When changing the polymerization rate at the free iMT plus end, the spindle length and overlap length are automatically tuned to be proportional to the flux rate. We analyze the effects of the changes in the parameters related to MT depolymerization at the minus end and those related to kMT polymerization at the plus end on the dynamics of the spindle. Our stable metaphase spindle model can predict the spindle’s response to disturbances and help identify conditions that maintain spindle stability, thereby reducing the error rate of mitosis.

### Limitations of the study

In this work, we show that the kinetochores can oscillate slowly with a large amplitude, which is consistent with the prior experimental observations.^27^^,^^63^^,^^64^^,^^65^ However, the mechanism of how the large-amplitude oscillation can occur is not explained, which requires exploration in the future. Moreover, in the work we only consider the correct bio-oriented attachments of kMT to the kinetochore and have not considered the detachment of kMT from the kinetochore and the kMT reattachment to the kinetochore. Future studies are required to include the kMT detachment/reattachment and investigate how the erroneous attachments of kMT to the kinetochore can be corrected using our model proposed here. In addition, there are many proteins involved in driving or resisting MT sliding, providing pushing or pulling force other than kinesin-5, such as kinesin-12, passive crosslinker, etc.^6^^,^^17^^,^^68^ The future studies should include these aspects.

## Resource availability

### Lead contact

Further information and requests for resources and reagents should be directed to and will be fulfilled by the Lead Contact, Ping Xie (pxie@aphy.iphy.ac.cn).

### Materials availability

This study did not generate new unique reagents.

### Data and code availability

• All data reported in this article will be shared by the lead contact upon reasonable request.
• All original codes have been deposited at Zenodo and are publicly available as of the date of publication. Accession numbers are listed in the key resources table.
• Any additional information required to reanalyze the data reported in this article is available from the lead contact upon request.

## Acknowledgments

This study is supported by the 10.13039/501100001809National Natural Science Foundation of China (12122402), and the 10.13039/501100012226Fundamental Research Funds for the Central Universities.

## Author contributions

P.X. designed and organized the research; Y.W. wrote the simulation code and performed simulations; Y.-R. L., H.L., and P.-Y.W. assisted in doing research and discussed results; Y.W. and P.X. wrote the article. All authors reviewed the article.

## Declaration of interests

The authors declare no competing interest.

## STAR★Methods

### Key resources table

**Table:** 

REAGENT or RESOURCE	SOURCE	IDENTIFIER
**Software and algorithms**		

Python version 3.9	Python Software Foundation	https://www.python.org/
Original code and analysis	This paper	https://doi.org/10.5281/zenodo.16792415

### Method details

#### Model of kinesin-5

Kinesin-5 is a homotetramer, having two pairs of heads at opposite ends of a common stalk. The two pairs of heads act as two independent dimeric motors that can move processively toward the MT plus end. We use the model and dynamics of the full-length kinesin-5 motor as described in details before (see Methods S1).^34^^,^^61^

#### Model of kinesin-13

Kinesin-13 can bind to one tubulin on MT with rate, $k_{\text{on}}^{\left(K13\right)}=k_{\text{on}0}^{\left(K13\right)}\left[K13\right]$, where $k_{\text{on}}^{\left(K13\right)}$ is the second-order binding rate and [K13] is the kinesin-13 concentration. After binding to MT, kinesin-13 can make unbiased diffusion along the MT filament with diffusion constant *D*_K13_ and can dissociate from MT with rate $k_{\text{off}}^{\left(K13\right)}$. The poleward flux can transport kinesin-13 bound to MT toward the minus end. After reaching the minus end, kinesin-13 can depolymerize MT processively with rate *k*_dep_ for a time period $\tau_{\text{end}}$ before dissociation, as previous studies indicated.^35^^,^^36^ For both kMTs and iMTs, if a pushing force acts on the minus end, *k*_dep_ = $k_{\text{dep}}^{\left(+\right)}$, and if a pulling force acts on the minus end, *k*_dep_ = $k_{\text{dep}}^{\left(-\right)}$. The activity of kinesin-13 can be schematically depicted in Figure S19B.

#### Model of NuMA

NuMA has two MT-binding heads at opposite ends of a long stalk. For multiple ensembles of MTs, NuMA can crosslink two distant parallel iMTs. NuMA can bind to one tubulin on MT with rate $k_{\text{on}}^{\left(\text{NuMA}\right)}=k_{\text{on}0}^{\left(\text{NuMA}\right)}\left[\text{NuMA}\right]$, where $k_{\text{on}0}^{\left(\text{NuMA}\right)}$ is the second-order binding rate and [NuMA] is the NuMA concentration. When one head binds to a MT, another head can bind to the parallel distant MT with rate $\mu_{\text{NuMA}}$. We do not consider the movement of the attached NuMA head relative to MT, based on the evidence that NuMA moves at a similar rate to the flux rate.^6^^,^^69^ When the tubulin bound by the NuMA head is depolymerized, the head dissociates.

#### The choice of parameter values

For human cells, the choice of the parameter values is discussed as follows. The parameter values of full-length kinesin-5 Eg5 for the First set of values are the same as those determined before^34^^,^^61^ (Methods S2 and Table S1). Since the flux rate under the condition of Risteski et al.^6^ is evidently larger than that under the condition of Steblyanko et al.,^17^ we take values of the parameters related to the velocity for the Second set of values to be 2 times of those for the First set of values while values of other parameters being the same (Table S1). The parameter values of kinesin-13 MACK motor are discussed in Methods S2 and listed in Table S2. The value of *v*_p0_ is taken as a preset one, which in the experiments was varied by the depletion of the plus-end tracking proteins, such as polymerase enzymes, kinesin-8 motors, etc.^6^^,^^16^^,^^17^^,^^18^^,^^30^^,^^46^^,^^47^ Other parameters related to the polymerization are discussed in Methods S2 and listed in Table S4. The parameter values of NuMA are discussed in Methods S2 and listed in Table S3. The values of elastic constants of the three springs are discussed in Methods S2 and listed in Table S5.

For zebrafish cells, the parameter values for kinesin-5 and kinesin-13 motors are given in Tables S6 and S7, respectively. The value of *v*_p0_ is still taken as a preset one, which in the experiment was varied due to the variation of the cell volume.^38^ The parameters for the polymerization of stem kMTs at the plus end are still taken as the Second set of values listed in Table S4. The parameters for the three spring elastic constants are still taken as those listed in Table S5.

#### Brief mathematical descriptions of the temporal evolution of overlap length and spindle length

The overlap length formed by iMT1 and iMT2 pairs, $L_{\text{OL}}$, is determined by their polymerization rates at plus end and their poleward sliding rates. The temporal evolution of $L_{\text{OL}}$ can be expressed as $dL_{\text{OL}}/dt=v_{\text{pol}}^{\left(\text{iMT}1\right)}-v_{\text{slide}}^{\left(\text{iMT}1\right)}+v_{\text{pol}}^{\left(\text{iMT}2\right)}-v_{\text{slide}}^{\left(\text{iMT}2\right)}$, where $v_{\text{pol}}^{\left(\text{iMT}1\right)}$ and $v_{\text{pol}}^{\left(\text{iMT}2\right)}$ are the polymerization rate at plus end of iMT1 and iMT2, respectively, with the average values of $v_{\text{pol}}^{\left(\text{iMT}1\right)}$ and $v_{\text{pol}}^{\left(\text{iMT}2\right)}$ equal to $v_{p0}$, while $v_{\text{slide}}^{\left(\text{iMT}1\right)}$ and $v_{\text{slide}}^{\left(\text{iMT}2\right)}$ are the poleward sliding rates of iMT1 and iMT2, respectively, driven by the kinesin-5 motors. The spindle length, $X_{\text{SL}}$, is determined by depolymerization rates and the poleward sliding rates of the iMTs and kMTs. For 1 ensemble of MTs, the temporal evolution of the spindle length can be expressed as $dX_{\text{SL}}/dt=\left(v_{\text{slide}}^{\left(\text{iMT}1\right)}-v_{\text{dep}}^{\left(\text{iMT}1\right)}+v_{\text{slide}}^{\left(\text{iMT}2\right)}-v_{\text{dep}}^{\left(\text{iMT}2\right)}+v_{\text{slide}}^{\left(\text{kMT}1\right)}-v_{\text{dep}}^{\left(\text{kMT}1\right)}+v_{\text{slide}}^{\left(\text{kMT}2\right)}-v_{\text{dep}}^{\left(\text{kMT}2\right)}\right)/2$, where $v_{\text{dep}}^{\left(\text{iMT}1\right)}$ and $v_{\text{dep}}^{\left(\text{iMT}2\right)}$ are the depolymerization rates at the minus end of iMT1 and iMT2, respectively, $v_{\text{dep}}^{\left(\text{kMT}1\right)}$ and $v_{\text{dep}}^{\left(\text{kMT}2\right)}$ are the depolymerization rates at the minus end of kMT1 and kMT2, respectively, while $v_{\text{slide}}^{\left(\text{kMT}1\right)}$ and $v_{\text{slide}}^{\left(\text{kMT}2\right)}$ are the poleward sliding rates of kMT1 and kMT2, respectively.

#### Simulation methods

The binding to MT, stepping on MT and dissociating from MT of kinesin-5 and kinesin-13, the binding of NuMA as well as the MT polymerase and depolymerase activities, are simulated with the Monte-Carlo method, as usually done in the literature.^57^ The simulation methods and procedures are described in detail in Methods S3–S7.

### Quantification and statistical analysis

Each data point represents five simulation trials for more than 10000 seconds. Error bars represent the standard error of the mean (SEM) except where noted.

## Footnotes

Supplemental information can be found online at https://doi.org/10.1016/j.isci.2025.113506.
