# Supplementary material for: Mechanism of spindle stability and poleward flux regulating spindle length during the metaphase
Source: iScience. 2025 Sep 4;28(10):113506. doi: 10.1016/j.isci.2025.113506 (PMC12478053; doi:10.1016/j.isci.2025.113506)
Supplement: Document S1. Figures S1–S19 and Tables S1–S7 [file mmc1.pdf]

iScience, Volume *28*

Supplemental information

Mechanism of spindle stability and poleward flux regulating spindle length during the metaphase

Yao Wang, Yu-Ru Liu, Peng-Ye Wang, Hui Li, and Ping Xie

Document S1

Methods S1: Calculations of stepping rates and dissociation rate of the single kinesin-5 motor on the single MT

In this paper, we consider full-length kinesin-5 motor, as done before [S1, S2]. We focus on physiological or saturating ATP concentrations. Before presenting equations for the forward stepping rate, backward stepping rate and dissociation rate of a kinesin dimer, we define some parameters, as done before [S2-S5]. We denote by $k^{(+)}$ the rate of ATP transition to ADP in the head with the forward NL orientation (e.g., the trailing head) and by $k^{(-)}$ the rate of ATP transition to ADP in the head without the forward NL orientation (e.g., the leading head). Rates $k^{(+)}$ and $k^{(-)}$ are independent of the force on the NLs [S3 – S5]. We denote by k_D the rate of ADP release from the head bound to MT, which for simplicity, is treated to be independent of NL direction and force on NL. Since both the rate of weakening the affinity of the MT-bound ATP-head to the detached ADP-head and the rate of NL docking of the MT-bound ATP-head is determined by the rate of the large conformational change of the ATP-head, the three rates have approximately the same values. Thus, we use k_{NL} to represent both the rate of NL docking and that of weakening between the two heads. As mentioned before [S4 – S6], to be consistent with the available experimental data, these rates $k^{(+)}$, $k^{(-)}$, k_D and rate k_{NL} are independent of the load on the motor.

During the processive motion of the kinesin-5 dimer on a MT, the overall ATPase rate of the trailing head can be approximately written as [S2]

$$k_T = P_{E1}\left(\frac{1}{k^{(+)}} + P_{0T}\frac{1}{k_r}\right)^{-1} + (1 - P_{E1})\left(\frac{1}{k_D} + \frac{1}{k_r} + \frac{1}{k^{(+)}}\right)^{-1}, \tag{S1}$$

where $P_{0T} = (1/k_D + 1/k_r)/(1/k_D + 1/k_r + 1/k^{(+)})$, k_r is the rate of the tail domain releasing from the nucleotide-free head. The rate k_r is also independent of the load on the motor. The overall ATPase rates of the leading head can be approximately written as [S2]

$$k_L = P_{E2}\left(\frac{1}{k_D} + \frac{1}{k_r} + \frac{1}{k^{(-)}}\right)^{-1} + (1 - P_{E2})\left(\frac{1}{k^{(-)}} + P_{0L}\frac{1}{k_r}\right)^{-1}, \tag{S2}$$

where $P_{0L} = (1/k_D + 1/k_r)/(1/k_D + 1/k_r + 1/k^{(-)})$. In Eqs. (S1) and (S2), probabilities P_{E1} and P_{E2} can be written as [S7]

$$P_{E1} = \frac{\exp(\beta E_D)\exp[\beta\lambda(\varepsilon_0 - \varepsilon_1)]}{\exp(\beta E_D)\exp[\beta\lambda(\varepsilon_0 - \varepsilon_1)] + 1}, \tag{S3}$$

$$P_{E2} = \frac{\exp(\beta E_D)\exp[\beta\lambda(\varepsilon_{-1} - \varepsilon_0)]}{\exp(\beta E_D)\exp[\beta\lambda(\varepsilon_{-1} - \varepsilon_0)] + 1}, \tag{S4}$$

where E_D is the energy change associated with both the NL docking and the conformational change of the head induced by ATP binding, λ is the energy-slitting factor, $\beta^{-1} = k_B T$ the thermal energy, ε_0 is the potential energy of the motor before taking the step, ε_1 is the potential energy after taking the forward step, and ε_{-1} is the potential energy after taking the backward step. As determined before [S6], λ = 1 for the

full-length kinesin-5 Eg5 motor. It is noted that P_{E1} and P_{E2} depend on the load acting on the motor via the dependences of $\varepsilon_0 - \varepsilon_1$ and $\varepsilon_{-1} - \varepsilon_0$ upon the load.

The forward stepping rate of the motor can be written as [S2]

$$k_F^{(m)} = P_{E1} k_T . \tag{S5}$$

The backward stepping rate of the motor can be written as [S2]

$$k_B^{(m)} = (1 - P_{E2}) k_L . \tag{S6}$$

The velocity of the motor can then be written as

$$v = \left[P_{E1} k_T - (1 - P_{E2}) k_L \right] d , \tag{S7}$$

where $d = 8.2$ nm is the step size, equal to the period of tubulins on a MT filament. From Eqs. (S1) – (S7), it is noted that the dependence of v on the load is via the dependences of P_{E1} and P_{E2} on the load, because $k^{(+)}$, $k^{(-)}$, k_D, k_{NL} and k_r are independent of the load (see above).

The dissociation of the motor can occur in both the weak MT-binding state and strong MT-binding state. The period of the weak MT-binding state includes Period I and Period II [S2, S6, S8]. In Period I, one ADP-head binds to the local tubulin with the very weak affinity E_{w1} and the other detached ADP-head has the high binding energy to the MT-bound head. In Period II, one ADP-head binds to MT with the weak affinity E_{w2} (>>E_{w1}) and the other detached ADP-head has the high binding energy to the MT-bound head.

The dissociation rate of the motor can be written as [S2, S6, S8]

$$k_{off}^{(m)} = k_T P_I P_{dI} + \left(k_T P_{II}^{(T)} + k_L P_{II}^{(L)} \right) P_{dII} + \varepsilon_s , \tag{S8}$$

where P_I is the occurrence probability of Period I, P_{dI} is the dissociation probability during Period I, $P_{II}^{(T)}$ and $P_{II}^{(L)}$ are the occurrence probability of Period II, P_{dII} is the dissociation probability during Period II, and ε_s is the dissociation rate in the strong MT-binding state. Since during the chemomechanical cycle the weak MT-binding state only occurs occasionally and if occurs its lifetime is much shorter than the lifetime of a chemomechanical cycle, for simplicity, it is considered here that the motor is almost always in the strong MT-binding state.

P_I can be written as [S2]

$$P_I = \frac{k}{k_{NL} + k} , \tag{S9}$$

$$k = k^{(-)} , \qquad \text{when } F \le 0, \tag{S10}$$

$$k = k^{(-)} + \frac{k^{(+)} - k^{(-)}}{4} F , \qquad \text{when } 0 < F < 4 \text{ pN}, \tag{S11}$$

$$k = k^{(+)} , \qquad \text{when } F \ge 4 \text{ pN}. \tag{S12}$$

In Period I, since E_{w1} is very small the motor can dissociate from MT with a nearly 100% probability even under no load, giving the dissociation probability in Period I $P_{dI} \approx 1$ under any load.

$P_{II}^{(T)}$ and $P_{II}^{(L)}$ can be written as [S2, S6, S8]

$$P_{\mathrm{II}}^{(\mathrm{T})} = P_{\mathrm{E1}} \frac{k^{(+)}}{k^{(+)} + k_{\mathrm{D}}} + \left(1 - P_{\mathrm{E1}}\right) \frac{k^{(-)}}{k^{(-)} + k_{\mathrm{D}}}, \tag{S13a}$$

$$P_{\mathrm{II}}^{(\mathrm{L})} = P_{\mathrm{E2}} \frac{k^{(+)}}{k^{(+)} + k_{\mathrm{D}}} + \left(1 - P_{\mathrm{E2}}\right) \frac{k^{(-)}}{k^{(-)} + k_{\mathrm{D}}}. \tag{S13b}$$

The dissociation probability, P_{dII}, in Period II can be calculated with [S2, S6, S8]

$$P_{\mathrm{dII}} = \frac{k_{\mathrm{dII}}}{k_{\mathrm{dII}} + k_{\mathrm{D}}}, \tag{S14}$$

$$k_{\mathrm{dII}} = \varepsilon_{\mathrm{w0}} \exp\left(\beta |F| \delta_{\mathrm{w}}\right), \tag{S15}$$

where k_{dII} is the dissociation rate during Period II, with $\varepsilon_{\mathrm{w0}}$ being the dissociation rate under no load and δ_{w} being the distance parameter for the dissociation.

The dissociation rate in the strong MT-binding state can be written as

$$\varepsilon_{\mathrm{s}} = \varepsilon_{\mathrm{s0}} \exp\left(|F| \delta_{\mathrm{s}} / k_B T\right), \tag{S16}$$

where $\varepsilon_{\mathrm{s0}}$ is the dissociation rate under no load on the motor and δ_{s} is the load-sensitivity distance parameter for the dissociation [S6, S8].

From Eqs. (S1) – (S4) and (S8) – (S16), it is noted that the dependence of $k_{\mathrm{off}}^{(\mathrm{m})}$ on the load is via the dependences of P_{E1}, P_{E2}, P_{I}, k_{dII} and ε_{s} on the load, because $k^{(+)}$, $k^{(-)}$, k_{D}, k_{NL} and k_{r} are independent of the load.

For the case of the single full-length kinesin-5 motor moving on the single MT in the single-molecule optical trapping experiments with the moving trap to ensure a constant load, $\varepsilon_0 - \varepsilon_1 = \kappa x^2/2 - \kappa(x+d)^2/2 = -Fd - \kappa d^2/2$ and $\varepsilon_{-1} - \varepsilon_0 = \kappa(x-d)^2/2 - \kappa x^2/2 = -Fd + \kappa d^2/2$, where κ is the effective elastic constant of the trapping and motor stalk and $F = \kappa x$ is the load on the motor [S7]. Since the optical trapping has a much smaller elastic constant of about 0.05 pN/nm than the motor stalk, we approximately have κ = 0.05 pN/nm. With above expressions for $\varepsilon_0 - \varepsilon_1$ and $\varepsilon_{-1} - \varepsilon_0$, Eqs. (S3) and (S4) become [S7]

$$P_{\mathrm{E1}} = \frac{\exp\left[\beta E_{\mathrm{D}} - \lambda\left(Fd + \kappa_{\mathrm{trap}} d^2/2\right)\right]}{\exp\left[\beta E_{\mathrm{D}} - \lambda\left(Fd + \kappa_{\mathrm{trap}} d^2/2\right)\right] + 1}, \tag{S17}$$

$$P_{\mathrm{E2}} = \frac{\exp\left[\beta E_{\mathrm{D}} - \lambda\left(Fd - \kappa_{\mathrm{trap}} d^2/2\right)\right]}{\exp\left[\beta E_{\mathrm{D}} - \lambda\left(Fd - \kappa_{\mathrm{trap}} d^2/2\right)\right] + 1}. \tag{S18}$$

With Eqs. (S1), (S2) and (S5) – (S18) and parameter values of the full-length kinesin-5 Eg5 motor for the First set of values (Table S1), the calculated velocity and dissociation rate of the single motor versus F are shown in Fig. S1. It is seen that the theoretical results of the velocity versus F are consistent with the available single molecule optical trapping data [S9].

Throughout, on the basis of the force dependencies of velocity and dissociation rate for the full-length Eg5 motor, as shown in Fig. S1, we study the spindle dynamics. We have checked that using the simple

linear form of velocity versus force (Fig. S2A), where the force in one mechanical step is equal to the average value of the force on Eg5 motor before it taking the mechanical step and the one after it taking the mechanical step, and the same form of dissociation rate versus force as shown in Fig. S1B, we can obtain the similar results for the spindle dynamics. Some numerical results are shown in Fig. S2B. It is seen that the spindle length increases with the increase of v_{p0} that is equal to the flux rate, similar to Fig. 3.

Methods S2: The choice of parameter values for human cells

For the full-length kinesin-5 Eg5, the values of $k^{(+)}$, $k^{(-)}$, E_D, k_{NL}, k_D, k_r, ε_{w0} and δ_w are shown in Table S1, where the parameter values for the First set of values were determined before [S2] by fitting to the available experimental data [S9 – S11] while values of $k^{(+)}$, $k^{(-)}$, E_D, k_{NL}, k_D and k_r for the Second set of values are taken to be two times of those for the First set of values. We take ε_{s0} = 0.1 s^{-1}, as taken before for kinesin-1 [S6], and δ_s = 2.3 nm (Table S1). We fix kinesin-5 concentration [K5] = 3 nM (Table S1). As done before [S1], we denote by $k_{on}^{(m)} = k_{on0}^{(m)}[K5]$ the MT-binding rate of the kinesin-5 motor, with $k_{on0}^{(m)}$ being the second-order MT-binding rate and [K5] being the kinesin-5 concentration, and by μ_m the binding rate of one pair of the heads to one MT when another pair of the heads at the opposite end of the stalk are attached to another MT in the antiparallel overlap zone, with their values shown in Table S1 [S1]. The stalk of the motor behaves elastically as a spring of the elastic constant K_E. As done before [S1], we take K_E = 0.55 pN/nm (Table S1). The activity of kinesin-5 can be schematically depicted in Fig. S19A.

The parameter values of kinesin-13 MACK motor are chosen as follows. The experimental data gave the second-order MT-binding rate of kinesin-13 MCAK of $k_{on0}^{(K13)}$ = 0.0005 $nM^{-1}s^{-1}nm^{-1}$ [S12]. Thus, we take $k_{on0}^{(K13)}$ = 0.0001 nM^{-1} $s^{-1}nm^{-1}$ for the First set of values and $k_{on0}^{(K13)}$ = 0.0003 nM^{-1} $s^{-1}nm^{-1}$ for the Second set of values. We fix [K13] = 1 nM. We take the dissociation rate $k_{off}^{(K13)}$ = 0.001 s^{-1} for the First set of values and $k_{off}^{(K13)}$ = 0.003 s^{-1} for the Second set of values. The experimental data showed the diffusion constant of D_{K13} = 5510 nm^2/s [S13], giving the forward or backward stepping rate $k_{diff}^{(K13)} = D_{K13}/d^2$ = 82 s^{-1} (d = 8.2 nm). Since a pushing force on the minus end of iMT can induce the tubulins near the minus end to be more curved than no force while a pulling force on the minus end of kMT can induce the tubulins near the minus end to be less curved, the depolymerization rate of iMT may be slightly larger than that of kMT. Thus, we take the depolymerization rates of iMT and kMT as $k_{dep}^{(+)}$ = 5 s^{-1} and $k_{dep}^{(-)}$ = 4 s^{-1}, respectively. Note that these depolymerization rates of 4 ~ 5 s^{-1} are consistent with that estimated before [S14]. We take the MT-end residence time τ_{end} = 2 s, which is consistent with the experimental data for MCAK [S15]. The parameter values for kinesin-13 are summarized in Table S2.

The parameters related to the polymerization are chosen as follows. The value of v_{p0} is taken as a preset one, which in the experiments was varied by the depletion of the plus-end tracking proteins, such as polymerase enzymes, kinesin-8 motors, etc. [S16 – S21]. We take B = 4 for both the First and Second sets of values (Table S4). For multiple ensembles of MTs, we take F_{p0} = 3.2 pN for the First set of values and F_{p0} =

1.85 pN for the Second set of values (Table S4).

The parameter values of NuMA are chosen as follows. Considering that the stalk of NuMA has a long (200-nm) stalk [S22], we take its spring coefficient $K_{\rm NuMA}$ = 0.03 pN/nm. We take $k_{\rm on0}^{\rm (NuMA)}$ = 3×10^{-4} nM^{-1} s^{-1}site^{-1} and [NuMA] = 1 nM. The rebinding rate $\mu_{\rm NuMA}$ is taken as 1 s^{-1}. We have checked that varying $\mu_{\rm NuMA}$ has little effect on our results. The parameter values for NuMA are summarized in Table S3.

The elastic constants of the three springs are taken as follows. We take κ_1 having a large value of 10 pN/nm, as the distance between two kinetochores fluctuates little [S23 – S25]. We take κ_2 = 0.1 pN/nm, as taken before [S26, S27]. We take κ_3 = 0.1 pN/nm, as measured experimentally [S28]. The three parameter values are summarized in Table S5.

Methods S3: Kinetic Monte-Carlo simulation method

In the Monte-Carlo simulations of the stepping, binding and unbinding of kinesin-5 and kinesin-13, the binding of NuMA, as well as the polymerization and depolymerization activity of MTs, we take the time step $h = 10^{-3}$ s. We have checked that doubling the time step h does not affect our results.

For each kinesin-5 motor in the overlap region, we take 4 independent random variables uniformly distributed between 0 and 1, *ran*1, *ran*2, *ran*3 and *ran*4. During each time step h, if *ran*1 < $k_{\rm off}^{\rm (m)} h$, the pair of heads bound to the MT detaches from the MT. If *ran*2 < $k_{\rm F}^{\rm (m)} h$, the pair of heads bound to the MT takes a forward step, and if *ran*3 < $k_{\rm B}^{\rm (m)} h$, the pair of heads bound to the MT takes a backward step. When one pair of heads is detached from the MT and another pair of heads of the kinesin-5 motor is bound to another MT in the overlap zone, if *ran*4 < $\mu_{\rm m} h$, the detached pair of heads rebinds to the MT. For the kinesin-5 motor in the solution binding to one MT, we take 1 independent random variable uniformly distributed between 0 and 1, *ran*5. If *ran*5 < $k_{\rm on}^{\rm (m)}[{\rm K5}]h$, where [K5] = 3 nM is the kinesin-5 concentration, one pair of heads of an kinesin-5 motor in the solution binds to an unoccupied tubulin of MT.

For the stepping, binding and unbinding of kinesin-13, we take 4 independent random variables uniformly distributed between 0 and 1, *ran*6, *ran*7, *ran*8 and *ran*9. During each time step h, if *ran*6 < $k_{\rm diff}^{\rm (K13)}$ h, the kinesin-13 bound to the MT takes a forward step, and if *ran*7 < $k_{\rm diff}^{\rm (K13)} h$ the kinesin-13 bound to the MT takes a backward step. Diffusion only occurs when adjacent tubulins are unoccupied. If *ran*8 <$k_{\rm off}^{\rm (K13)} h$ the kinesin-13 bound to the MT detaches from the MT. If *ran*9 < $k_{\rm on}^{\rm (K13)} dh$, the kinesin-13 in the solution binds to an unoccupied tubulin in the region that does not form anti-parallel MT overlap.

For MT polymerization at the plus end of each MT with the polymerization rate $k_{\rm P}$, we take one random variable uniformly distributed between 0 and 1, *ran*10. If *ran*10 < $k_{\rm P}h$, one tubulin is added to the plus end of the MT, increasing the MT length by d. For the iMT, the polymerization rate $k_{\rm P} = v_{\rm P0}/d$ is constant. For the kMT, the polymerization rate $k_{\rm P}$ = $v_{\rm pol}^{\rm (kMT)}/d$ is dependent on the force on the plus end, as calculated by Eq. (1)

(see the main text).

For MT depolymerization at the MT minus end of each MT with the depolymerization rate k_{dep}, we take one random variable uniformly distributed between 0 and 1, *ran*11. If a kinesin-13 protein is located at the nearest binding tubulin from the minus end and *ran*11 < $k_{\mathrm{dep}}h$, one tubulin heterodimer is removed from the minus end of the MT, decreasing the MT length by *d*. If the second binding tubulin closest to the MT minus end is occupied, the kinesin-13 protein detaches from MT after depolymerizing the nearest tubulin. If the second binding tubulin closest to the minus end is unoccupied, the kinesin-13 takes a backward step after depolymerizing the nearest tubulin, remaining bound to the minus end. For both kMT and iMT, if a pushing force is exerted on the MT minus end, $k_{\mathrm{dep}} = k_{\mathrm{dep}}^{(+)}$, and if a pulling force is exerted on the MT minus end, $k_{\mathrm{dep}} = k_{\mathrm{dep}}^{(-)}$.

For *N* ensembles of MTs, NuMA protein is considered to crosslink two parallel iMTs. For NuMA in the solution binding to one iMT, we take 1 independent random variable uniformly distributed between 0 and 1, *ran*12. If *ran*12 < $k_{\mathrm{on}}^{(\mathrm{NuMA})}h$, one MT-binding domain of NuMA in the solution binds to an unoccupied tubulin of iMT. For NuMA with one MT-binding domain connected to one iMT, we take one independent random variable uniformly distributed between 0 and 1, *ran*13. During each time step *h*, if *ran*13 < $\mu_{\mathrm{NuMA}}\, h$, the detached MT-binding domain of NuMA binds to the parallel iMT.

Note that the activities of kinesin-5, kinesin-13, NuMA, MT polymerization and MT depolymerization are independent of each other.

Methods S4: Method for simulations of spindle system of one ensemble of MTs

To simulate the movement of the spindle poles, kinetochores and MTs, we arrange two antiparallel iMTs to overlap in the spindle midzone and make kMTs connect the kinetochores and poles (Fig. 1). The simulation procedure is described as follows.

We take a random number of kinesin-5 motors that are positioned randomly within the overlap region formed by the antiparallel MTs. The two MT-binding domains of the kinesin-5 motor on opposite sides of the stalk connect to two antiparallel MTs. We take a random number of kinesin-13 proteins that are positioned randomly on the MT non-overlap region, with each kinesin-13 protein having one MT-binding domain binding to the MT. Regardless of the initial numbers and distributions of kinesin-5 and kinesin-13 proteins, the simulations show that the spindle system consistently reaches the same stationary state (video S1). The motions of each MT-binding domain are simulated using the Monte-Carlo methods described in Section S3.

First, we consider the case of one ensemble of MTs (Fig.1A). Three overlap regions are formed by three pairs of antiparallel MTs: one by iMT1 and iMT2, another one by iMT2 and kMT1, the third one by iMT1 and kMT2, which are termed as overlap 1, overlap 2, and overlap 3, respectively. Kinesin-5 proteins are located in these three overlap zones. We denote by $\Delta x_i^{(\alpha)}$ (α =1, 2, 3 representing the three antiparallel

overlap zones) the ith motor's center-of-mass position of the pair of heads bound to one MT relative to that of another pair of heads bound to another MT along the x direction or the filament direction. When $\left|\Delta x_i^{(\alpha)}\right|$ $\leq$ l_0 the stalk of kinesin-5 motor is not stretched and when $\left|\Delta x_i^{(\alpha)}\right|$ $>$ l_0 the stalk is stretched, as all-atom molecular dynamics simulations showed that the motor's stalk can rotate freely relative to the head in a large range of angles [S29]. For the simulations, we take l_0 = 8.2 nm here. Thus, the extension of the ith motor's stalk is $\xi_i^{(\alpha)}=\left(\Delta x_i^{(\alpha)}-l_0\right)$ when Δx_i $>$ l_0, $\xi_i^{(\alpha)}=\left(\Delta x_i^{(\alpha)}+l_0\right)$ when $\Delta x_i^{(\alpha)}$ $<-l_0$ and $\xi_i^{(\alpha)}$ $=0$ when $\left|\Delta x_i^{(\alpha)}\right|$ $<$ l_0. The ith motor experiences an external elastic force $F_i^{(\mathrm{m})}=K_E\xi_i^{(\alpha)}$. We denote by $X_{\mathrm{pole}}^{(\mathrm{left})}(t)$ ($X_{\mathrm{pole}}^{(\mathrm{right})}(t)$) the center-of-mass position of the left (right) spindle pole at moment t along the x direction. We denote by $X_{\mathrm{b}}^{(1)}(t)$ ($X_{\mathrm{b}}^{(2)}(t)$) the position of $X_{\mathrm{pole}}^{(\mathrm{left})}(t)$ ($X_{\mathrm{pole}}^{(\mathrm{right})}(t)$) deviating away from the rest length of the spring connecting the minus end of left (right) MT and the left (right) spindle pole at moment t along the x direction. When the minus end is on the left (right) side of $X_{\mathrm{b}}^{(1)}(t)$ ($X_{\mathrm{b}}^{(2)}(t)$), the left (right) MT is pushed by the left (right) spindle pole. Conversely, when the minus end is on the right (left) side of $X_{\mathrm{b}}^{(1)}(t)$ ($X_{\mathrm{b}}^{(2)}(t)$), the left (right) MT is pulled by the left (right) spindle pole. Similarly, we denote by $X_{\mathrm{kinet}}^{(\mathrm{left})}(t)$ ($X_{\mathrm{kinet}}^{(\mathrm{right})}(t)$) the center-of-mass position of the left (right) kinetochore at moment t along the x direction. We denote by $X_{\mathrm{iMT1}}^{(\mathrm{minus})}(t)$ ($X_{\mathrm{iMT1}}^{(\mathrm{plus})}(t)$) the position of the minus (positive) end of the iMT connected to the left spindle pole at moment t along the x direction. We denote by $X_{\mathrm{iMT2}}^{(\mathrm{minus})}(t)$ ($X_{\mathrm{iMT2}}^{(\mathrm{plus})}(t)$) the position of the minus (positive) end of the iMT connected to the right spindle pole at moment t along the x direction. We denote by $X_{\mathrm{kMT1}}^{(\mathrm{minus})}(t)$ ($X_{\mathrm{kMT1}}^{(\mathrm{plus})}(t)$) the position of the minus (positive) end of the kMT connected to the left spindle pole at moment t along the x direction. We denote by $X_{\mathrm{kMT2}}^{(\mathrm{minus})}(t)$ ($X_{\mathrm{kMT2}}^{(\mathrm{plus})}(t)$) the position of the minus (positive) end of the kMT connected to the right spindle pole at moment t along the x direction (Fig. 1A). After a time period h, the positions can be written as $X_{\mathrm{pole}}^{(\mathrm{left})}(t+h)=X_{\mathrm{pole}}^{(\mathrm{left})}(t)+z_1$, $X_{\mathrm{pole}}^{(\mathrm{right})}(t+h)=X_{\mathrm{pole}}^{(\mathrm{right})}(t)+z_2$, $X_{\mathrm{b}}^{(1)}(t+h)=X_{\mathrm{b}}^{(1)}(t)+z_1$, $X_{\mathrm{b}}^{(2)}(t+h)=X_{\mathrm{b}}^{(2)}(t)+z_2$, $X_{\mathrm{kinet}}^{(\mathrm{left})}(t+h)=X_{\mathrm{kinet}}^{(\mathrm{left})}(t)+z_3$, $X_{\mathrm{kinet}}^{(\mathrm{right})}(t+h)=X_{\mathrm{kinet}}^{(\mathrm{right})}(t)+z_4$, $X_{\mathrm{iMT1}}^{(\mathrm{minus})}(t+h)=X_{\mathrm{iMT1}}^{(\mathrm{minus})}(t)+z_5$, $X_{\mathrm{iMT1}}^{(\mathrm{plus})}(t+h)=X_{\mathrm{iMT1}}^{(\mathrm{plus})}(t)+z_5$, $X_{\mathrm{iMT2}}^{(\mathrm{minus})}(t+h)=X_{\mathrm{iMT2}}^{(\mathrm{minus})}(t)+z_6$, $X_{\mathrm{iMT2}}^{(\mathrm{plus})}(t+h)=X_{\mathrm{iMT2}}^{(\mathrm{plus})}(t)+z_6$, $X_{\mathrm{kMT1}}^{(\mathrm{minus})}(t+h)=X_{\mathrm{kMT1}}^{(\mathrm{minus})}(t)+z_7$, $X_{\mathrm{kMT1}}^{(\mathrm{plus})}(t+h)=X_{\mathrm{kMT1}}^{(\mathrm{plus})}(t)+z_7$, $X_{\mathrm{kMT2}}^{(\mathrm{minus})}(t+h)=X_{\mathrm{kMT2}}^{(\mathrm{minus})}(t)+z_8$ and $X_{\mathrm{kMT2}}^{(\mathrm{plus})}(t+h)=X_{\mathrm{kMT2}}^{(\mathrm{plus})}(t)+z_8$, where z_1, z_2, z_3, z_4, z_5, z_6, z_7 and z_8 are the movement distances of the left spindle pole, right spindle pole, left kinetochore, right kinetochore, iMT1, iMT2, kMT1 and kMT2, respectively. z_1, z_2, z_3, z_4, z_5, z_6, z_7 and z_8 are positive when the moving directions are from left to right. Meanwhile, the MT-binding domains of proteins connected to iMT1, iMT2, kMT1 and kMT2 move the distances of z_5, z_6, z_7 and z_8 following the movement of iMT1, iMT2, kMT1 and kMT2,

respectively. The movement distances are determined as follows.

We denote by $N_{\mathrm{E}}^{(\alpha)}$ (α = 1, 2, 3 representing three antiparallel overlap zones) the total number of the kinesin-5 motors with two MT-binding domains connected to MTs. Considering that the stalk of kinesin-5 motor has a rest length l_0, we need to judge whether the connected kinesin-5 motors are under stress or not. We denote by $n_0^{(\alpha)}$ (α =1, 2, 3 representing three antiparallel overlap zones, as defined just above) the total number of the motors in the spindle under the condition of $\Delta x_i^{(\alpha)} < -l_0$ and by $n_1^{(\alpha)}$ the total number of the motors in the spindle under the condition of $\Delta x_i^{(\alpha)} > l_0$ at moment *t*. When the moving MT moves forward or backward, the *i*th motor can transit from the state with no external elastic force on it to the state with an external elastic force on it. In the spindle, we denote by $n_2^{(\alpha)}$ the total number of the motors that transition from the state with no external elastic force on it to the state with an external elastic force on it under the condition of $l_0 > \Delta x_i^{(\alpha)}(t) > -l_0$ and $\Delta x_i^{(\alpha)}(t+h) < -l_0$ and by $n_3^{(\alpha)}$ the total number of the motors that transition from the state with an external elastic force on it to the state with no external elastic force on it under the condition of $\Delta x_i^{(\alpha)}(t) > l_0$ and $-l_0 < \Delta x_i^{(\alpha)}(t+h) < l_0$ after the time period *h*. We denote by $n_4^{(\alpha)}$ the total number of the motors that transition from the state with no external elastic force on it to the state with an external elastic force on it under the condition of $-l_0 < \Delta x_i^{(\alpha)}(t) < l_0$ and $\Delta x_i^{(\alpha)}(t+h) > l_0$ and by $n_5^{(\alpha)}$ the total number of the motors that transition from the state with an external elastic force on it to the state with no external elastic force on it under the condition of $\Delta x_i^{(\alpha)}(t) < -l_0$ and $-l_0 < \Delta x_i^{(\alpha)}(t+h) < l_0$ after the time period *h*. For convenience, we rearrange the sequence $\Delta x_i^{(\alpha)}(t)$ into a new sequence $\Delta x_m^{(\alpha')}(t)$ in the order of $\Delta x_1^{(\alpha')}(t) \le \Delta x_2^{(\alpha')}(t) \le \cdots \le \Delta x_{N_E^{(\alpha)}}^{(\alpha')}(t)$. Considering that at moment *t,* the total force acting on the left spindle pole, right spindle pole, left kinetochore, right kinetochore, iMT1, iMT2, kMT1 and kMT2 is zero, we have

$$\kappa_3(X_{\mathrm{iMT1}}^{(\mathrm{minus})} - X_{\mathrm{b}}^{(1)}) + \kappa_3(X_{\mathrm{kMT1}}^{(\mathrm{minus})} - X_{\mathrm{b}}^{(1)}) = 0, \tag{S19}$$

$$\kappa_3(X_{\mathrm{iMT2}}^{(\mathrm{minus})} - X_{\mathrm{b}}^{(2)}) + \kappa_3(X_{\mathrm{kMT2}}^{(\mathrm{minus})} - X_{\mathrm{b}}^{(2)}) = 0, \tag{S20}$$

$$\kappa_2(X_{\mathrm{kMT1}}^{(\mathrm{plus})} - X_{\mathrm{kinet}}^{(\mathrm{left})}) + \kappa_1(X_{\mathrm{kinet}}^{(\mathrm{right})} - X_{\mathrm{kinet}}^{(\mathrm{left})} - d_{\text{K-K}}) = 0, \tag{S21}$$

$$\kappa_2(X_{\mathrm{kMT2}}^{(\mathrm{plus})} - X_{\mathrm{kinet}}^{(\mathrm{right})}) + \kappa_1(X_{\mathrm{kinet}}^{(\mathrm{left})} - X_{\mathrm{kinet}}^{(\mathrm{right})} + d_{\text{K-K}}) = 0, \tag{S22}$$

$$\begin{aligned}&\kappa_3(X_{\mathrm{iMT1}}^{(\mathrm{minus})} - X_{\mathrm{b}}^{(1)}) - \sum_{m=1}^{n_0^{(3)}} K_{\mathrm{E}}\left(\Delta x_m^{(3')}(t) + l_0\right) - \sum_{m=N_{\mathrm{E}}^{(3)}-n_1^{(3)}+1}^{N_{\mathrm{E}}^{(3)}} K_{\mathrm{E}}\left(\Delta x_m^{(3')}(t) - l_0\right) + \sum_{m=1}^{n_0^{(1)}} K_{\mathrm{E}}\left(\Delta x_m^{(1')}(t) + l_0\right)\\ &+\sum_{m=N_{\mathrm{E}}^{(1)}-n_1^{(1)}+1}^{N_{\mathrm{E}}^{(1)}} K_{\mathrm{E}}\left(\Delta x_m^{(1')}(t) - l_0\right) = 0\end{aligned}, \tag{S23}$$

$$\begin{aligned}&\kappa_3(X_{\mathrm{b}}^{(2)} - X_{\mathrm{iMT2}}^{(\mathrm{minus})}) + \sum_{m=1}^{n_0^{(2)}} K_{\mathrm{E}}\left(\Delta x_m^{(2')}(t) + l_0\right) + \sum_{m=N_{\mathrm{E}}^{(2)}-n_1^{(2)}+1}^{N_{\mathrm{E}}^{(2)}} K_{\mathrm{E}}\left(\Delta x_m^{(2')}(t) - l_0\right) + \sum_{m=1}^{n_0^{(1)}} K_{\mathrm{E}}\left(\Delta x_m^{(1')}(t) + l_0\right)\\ &+\sum_{m=N_{\mathrm{E}}^{(1)}-n_1^{(1)}+1}^{N_{\mathrm{E}}^{(1)}} K_{\mathrm{E}}\left(\Delta x_m^{(1')}(t) - l_0\right) = 0\end{aligned}, \tag{S24}$$

$$\sum_{m=1}^{n_0^{(2)}} K_\mathrm{E}\left(\Delta x_m^{(2')}(t)+l_0\right)+\sum_{m=N_\mathrm{E}^{(2)}-n_1^{(2)}+1}^{N_\mathrm{E}^{(2)}} K_\mathrm{E}\left(\Delta x_m^{(2')}(t)-l_0\right)+\kappa_3(X_\mathrm{kMT1}^{(\mathrm{minus})}-X_\mathrm{b}^{(1)})+\kappa_2(X_\mathrm{kinet}^{(\mathrm{left})}-X_\mathrm{kMT1}^{(\mathrm{plus})})=0\,, \quad \text{(S25)}$$

$$-\sum_{m=1}^{n_0^{(3)}} K_\mathrm{E}\left(\Delta x_m^{(3')}(t)+l_0\right)-\sum_{m=N_\mathrm{E}^{(3)}-n_1^{(3)}+1}^{N_\mathrm{E}^{(3)}} K_\mathrm{E}\left(\Delta x_m^{(3')}(t)-l_0\right)+\kappa_3(X_\mathrm{b}^{(2)}-X_\mathrm{kMT2}^{(\mathrm{minus})})+\kappa_2(X_\mathrm{kinet}^{(\mathrm{right})}-X_\mathrm{kMT2}^{(\mathrm{plus})})=0\,, \quad \text{(S26)}$$

where $d_\text{K-K}$ is the equilibrium distance (1 μm) between two kinetochores. Eqs. (S19–S26) include the force generated by kinesin-5 proteins (terms $\propto K_\mathrm{E}$), the force from the spring connecting the spindle pole and the minus end of MT (terms $\propto \kappa_3$), the force from the spring connecting the kinetochore and the plus end of kMT (terms $\propto \kappa_2$), and the force from the spring connecting the two kinetochores (terms $\propto \kappa_1$).

After the time period *h*, the stepping, binding and unbinding of kinesin-5, as well as the polymerization and depolymerization activity of MTs, may influence the force equilibrium of the left spindle pole, right spindle pole, left kinetochore, right kinetochore, iMT1, iMT2, kMT1 and kMT2. We denote the forces acting on the left spindle pole, right spindle pole, left kinetochore, right kinetochore, iMT1, iMT2, kMT1 and kMT2 as F_1, F_2, F_3, F_4, F_5, F_6, F_7 and F_8, respectively (where positive force is in the direction pointing right) after the stepping, binding and unbinding of kinesin-5, or the polymerization and depolymerization activity of MTs. To reach a new equilibrium, where the total force acting on the two spindle poles, two kinetochores and four MTs at moment *t* + *h* is zero, the two spindle poles, two kinetochores and four MTs will move. The center-of-mass position of one MT-binding domain of the kinesin-5 relative to another MT-binding domain, $\Delta x_i^{(\alpha)}$, will change. When $\Delta x_i^{(\alpha)}(t)$ decreases, we define $n_>^{(\alpha)}=n_1^{(\alpha)}-n_3^{(\alpha)}$ and $n_<^{(\alpha)}=n_0^{(\alpha)}+n_2^{(\alpha)}$. We denote by $F_\mathrm{K}^{(\alpha)}=\sum_{m=n_0^{(\alpha)}}^{n_0^{(\alpha)}+n_2^{(\alpha)}} K_\mathrm{E}(\Delta x_m^{(\alpha)}+l_0)$ the increase of the force and by $F_0^{(\alpha)}=\sum_{m=N_\mathrm{E}^{(\alpha)}-n_1^{(\alpha)}+1}^{N_\mathrm{E}^{(\alpha)}-n_1^{(\alpha)}+n_3^{(\alpha)}+1} K_\mathrm{E}(\Delta x_m^{(\alpha)}-l_0)$ the decrease of the force because of the change of $\Delta x_i^{(\alpha)}$. When $\Delta x_i^{(\alpha)}(t)$ increases, we define $n_>^{(\alpha)}=n_1^{(\alpha)}+n_4^{(\alpha)}$ and $n_<^{(\alpha)}=n_0^{(\alpha)}-n_5^{(\alpha)}$. We denote by $F_\mathrm{K}^{(\alpha)}=\sum_{m=N_\mathrm{E}^{(\alpha)}-n_1^{(\alpha)}-n_4^{(\alpha)}+1}^{N_\mathrm{E}^{(\alpha)}-n_1^{(\alpha)}+1} K_\mathrm{E}(\Delta x_m^{(\alpha)}-l_0)$ the increase of the force and by $F_0^{(\alpha)}=\sum_{m=n_0^{(\alpha)}-n_5^{(\alpha)}}^{n_0^{(\alpha)}} K_\mathrm{E}(\Delta x_m^{(\alpha)}+l_0)$ the decrease of the force because of the change of $\Delta x_i^{(\alpha)}$. Thus, we have the following relations

$$\kappa_3(X_\mathrm{iMT1}^{(\mathrm{minus})}+z_5-X_\mathrm{b}^{(1)}-z_1)+\kappa_3(X_\mathrm{kMT1}^{(\mathrm{minus})}+z_7-X_\mathrm{b}^{(2)}-z_1)=-F_1\,, \quad \text{(S27)}$$

$$\kappa_3(X_\mathrm{iMT2}^{(\mathrm{minus})}+z_6-X_\mathrm{pole}^{(\mathrm{right})}-z_2)+\kappa_3(X_\mathrm{kMT2}^{(\mathrm{minus})}+z_8-X_\mathrm{b}^{(2)}-z_2)=-F_2\,, \quad \text{(S28)}$$

$$\kappa_2(X_\mathrm{kMT1}^{(\mathrm{plus})}+z_7-X_\mathrm{kinet}^{(\mathrm{left})}-z_3)+\kappa_1(X_\mathrm{kinet}^{(\mathrm{right})}+z_4-X_\mathrm{kinet}^{(\mathrm{left})}-z_3-d_\text{K-K})=-F_3\,, \quad \text{(S29)}$$

$$\kappa_2(X_\mathrm{kMT2}^{(\mathrm{plus})}+z_8-X_\mathrm{kinet}^{(\mathrm{right})}-z_4)+\kappa_1(X_\mathrm{kinet}^{(\mathrm{left})}+z_3-X_\mathrm{kinet}^{(\mathrm{right})}-z_4+d_\text{K-K})=-F_4\,, \quad \text{(S30)}$$

$$\begin{aligned}&\kappa_3(X_\mathrm{iMT1}^{(\mathrm{minus})}+z_5-X_\mathrm{b}^{(1)}-z_1)-\sum_{m=1}^{n_<^{(3)}} K_\mathrm{E}\left(\Delta x_m^{(3')}(t)+z_8-z_5+l_0\right)-\sum_{m=N_\mathrm{E}^{(3)}-n_>^{(3)}+1}^{N_\mathrm{E}^{(3)}} K_\mathrm{E}\left(\Delta x_m^{(3')}(t)+z_8-z_5-l_0\right)\\&+\sum_{m=1}^{n_<^{(1)}} K_\mathrm{E}\left(\Delta x_m^{(1')}(t)+z_5-z_6+l_0\right)+\sum_{m=N_\mathrm{E}^{(1)}-n_>^{(1)}+1}^{N_\mathrm{E}^{(1)}} K_\mathrm{E}\left(\Delta x_m^{(1')}(t)+z_5-z_6-l_0\right)=F_5\end{aligned}\,, \quad \text{(S31)}$$

$$\begin{aligned}&\kappa_3(X_\mathrm{b}^{(2)}+z_2-X_\mathrm{iMT2}^{(\mathrm{minus})}-z_6)+\sum_{m=1}^{n_<^{(2)}} K_\mathrm{E}\left(\Delta x_m^{(2')}(t)+z_7-z_6+l_0\right)+\sum_{m=N_\mathrm{E}^{(2)}-n_>^{(2)}+1}^{N_\mathrm{E}^{(2)}} K_\mathrm{E}\left(\Delta x_m^{(2')}(t)+z_7-z_6-l_0\right)\\&+\sum_{m=1}^{n_<^{(1)}} K_\mathrm{E}\left(\Delta x_m^{(1')}(t)+z_5-z_6+l_0\right)+\sum_{m=N_\mathrm{E}^{(1)}-n_>^{(1)}+1}^{N_\mathrm{E}^{(1)}} K_\mathrm{E}\left(\Delta x_m^{(1')}(t)+z_5-z_6-l_0\right)=-F_6\end{aligned}\,, \quad \text{(S32)}$$

$$\sum_{m=1}^{n_<^{(2)}} K_E\left(\Delta x_m^{(2')}(t)+z_7-z_6+l_0\right)+\sum_{m=N_E^{(2)}-n_>^{(2)}+1}^{N_E^{(2)}} K_E\left(\Delta x_m^{(2')}(t)+z_7-z_6-l_0\right)+\kappa_3(X_{\text{kMT1}}^{(\text{minus})}+z_7-X_b^{(1)}-z_1) \\ +\kappa_2(X_{\text{kinet}}^{(\text{left})}+z_3-X_{\text{kMT1}}^{(\text{plus})}-z_7)=F_7 \tag{S33}$$

$$-\sum_{m=1}^{n_<^{(3)}} K_E\left(\Delta x_m^{(3')}(t)+z_8-z_5+l_0\right)-\sum_{m=N_E^{(3)}-n_>^{(3)}+1}^{N_E^{(3)}} K_E\left(\Delta x_m^{(3')}(t)+z_8-z_5-l_0\right)+\kappa_3(X_b^{(2)}+z_2-X_{\text{kMT2}}^{(\text{minus})}-z_8) \\ +\kappa_2(X_{\text{kinet}}^{(\text{right})}+z_4-X_{\text{kMT2}}^{(\text{plus})}-z_8)=-F_8 \tag{S34}$$

To obtain the movement distance of two spindle poles, two kinetochores and four MTs, z_1, z_2, z_3, z_4, z_5, z_6, z_7 and z_8, we subtract Eq. (S19) from Eq. (S27), Eq. (S20) from Eq. (S28), Eq. (S21) from Eq. (S29), Eq. (S22) from Eq. (S30), Eq. (S23) from Eq. (S31), Eq. (S24) from Eq. (S32), Eq. (S25) from Eq. (S33) and Eq. (S26) from Eq. (S34), and we get eight linear equations for z_1, z_2, z_3, z_4, z_5, z_6, z_7 and z_8 as follows. In the equation $AZ = B$, A is the coefficient matrix and B is the constant matrix.

$$A=\begin{bmatrix}
-2\kappa_3 & 0 & 0 & 0 & \kappa_3 & 0 & \kappa_3 & 0 \\
0 & -2\kappa_3 & 0 & 0 & 0 & \kappa_3 & 0 & \kappa_3 \\
0 & 0 & -\kappa_1-\kappa_2 & \kappa_1 & 0 & 0 & \kappa_2 & 0 \\
0 & 0 & \kappa_1 & -\kappa_1-\kappa_2 & 0 & 0 & 0 & \kappa_2 \\
-\kappa_3 & 0 & 0 & 0 & \begin{matrix}(n_>^{(1)}+n_<^{(1)}+n_>^{(3)} \\ +n_<^{(3)})K_E+\kappa_3\end{matrix} & -(n_>^{(1)}+n_<^{(1)})K_E & 0 & -(n_>^{(3)}+n_<^{(3)})K_E \\
0 & \kappa_3 & 0 & 0 & (n_>^{(1)}+n_<^{(1)})K_E & \begin{matrix}-(n_>^{(1)}+n_<^{(1)}+n_>^{(2)} \\ +n_<^{(2)})K_E-\kappa_3\end{matrix} & (n_>^{(2)}+n_<^{(2)})K_E & 0 \\
-\kappa_3 & 0 & -\kappa_2 & 0 & 0 & -(n_>^{(2)}+n_<^{(2)})K_E & \begin{matrix}-(n_>^{(2)}+n_<^{(2)})K_E \\ +\kappa_2+\kappa_3\end{matrix} & 0 \\
0 & \kappa_3 & 0 & \kappa_2 & (n_>^{(3)}+n_<^{(3)})K_E & 0 & 0 & \begin{matrix}-(n_>^{(3)}+n_<^{(3)})K_E \\ -\kappa_2-\kappa_1\end{matrix}
\end{bmatrix}$$

$$Z=\begin{bmatrix} z_1 \\ z_2 \\ z_3 \\ z_4 \\ z_5 \\ z_6 \\ z_7 \\ z_8 \end{bmatrix}, \quad B=\begin{bmatrix} -F_1 \\ -F_2 \\ -F_3 \\ -F_4 \\ F_5+F_K^{(3)}-F_0^{(3)}-F_K^{(1)}+F_0^{(1)} \\ -F_6-F_K^{(2)}+F_0^{(2)}-F_K^{(1)}+F_0^{(1)} \\ F_7-F_K^{(2)}+F_0^{(2)} \\ -F_8+F_K^{(3)}-F_0^{(3)} \end{bmatrix}. \tag{S35}$$

To find a unique solution for the displacements, we impose the condition that the middle position of the two spindle poles is stationary, namely $z_1+z_2=0$. By solving the above equation, we get the movement distances of the two spindle poles, two kinetochores and four MTs: z_1, z_2, z_3, z_4, z_5, z_6, z_7 and z_8.

In short, the simulation process is briefly summarized as follows:

1. Initialize the positions of all elements in the spindle system.

1.1) Determine the rest positions of spring linkers connecting MTs and poles, $X_{\mathrm{b}}^{(1)}$ and $X_{\mathrm{b}}^{(2)}$, as well as the positions of the two kinetochores, $X_{\mathrm{kinet}}^{(\mathrm{left})}$ and $X_{\mathrm{kinet}}^{(\mathrm{right})}$.

1.2) Determine the positions of the minus and plus ends of the four MTs, $X_{\mathrm{iMT1}}^{(\mathrm{minus})}$, $X_{\mathrm{iMT1}}^{(\mathrm{plus})}$, $X_{\mathrm{kMT1}}^{(\mathrm{minus})}$, $X_{\mathrm{kMT1}}^{(\mathrm{plus})}$, $X_{\mathrm{iMT2}}^{(\mathrm{minus})}$, $X_{\mathrm{iMT2}}^{(\mathrm{plus})}$, $X_{\mathrm{kMT2}}^{(\mathrm{minus})}$ and $X_{\mathrm{kMT2}}^{(\mathrm{plus})}$.

1.3) Assign a random number of kinesin-5 motors at random positions in the overlap region, with either one pair of heads bound to one MT or two pairs of heads bound to the two MTs. Utilize two two-dimensional lists to characterize the state of kinesin-5. The first two-dimensional list contains information of the binding position of the two pairs of heads of kinesin-5 and that of the kinesin-5 number in the overlap region. The second two-dimensional list characterizes the binding status of the two pairs of heads of kinesin-5 to MTs. For example, the first two-dimensional list [[16.4,24.6], [41,41]] represents that there are two kinesin-5 motors bound in the overlap region, with the first kinesin-5 having the two pairs of heads at positions 16.4 and 24.6 nm, and the second kinesin-5 either having both pairs of heads bound to MTs at 41 nm or having one pair of heads bound to MT at 41 nm and the other pair of head being detached. Correspondingly, in the second two-dimensional list, the binding status of the first kinesin-5 is [1,1] and that of the second kinesin-5 is either [1,1], or [0,1] or [1,0], with '1' representing the pair of heads bound to MT and '0' representing the pair of heads being detached. For example, the second two-dimensional list [[1,1], [0,1]] represents that in the overlap region the first kinesin-5 has two pairs of heads bound to MTs and the second kinesin-5 has only one pair of heads bound to MT.

1.4) Assign a random number of kinesin-13 motors at random positions on each MT in the non-antiparallel region. Use four one-dimensional lists to characterize the number and positions of kinesin-13 motors on the four MTs.

2. Begin the loop. At each time step *h*, determine if there is any change in the state of the spindle apparatus (i.e., whether the parameters and lists mentioned in step 1 have changed).

2.1) Determine if each pair of MT-bound heads of kinesin-5 motor step forward, step backward, or detach. Determine if a pair of heads of a free kinesin-5 bind to MT or another pair of detached heads of the MT-bound kinesin-5 bind to MT.

2.1.1 If the pair of MT-bound heads of kinesin-5 motor step forward, step backward or detach, or if a pair of heads of a free kinesin-5 bind to the MT or another pair of detached heads of the MT-bound kinesin-5 bind to MT, firstly we make the corresponding change to the two-dimensional lists that characterize the status of kinesin-5, and then we determine if this change breaks the force balance on the four MTs, two kinetochores, or two spindle poles.

2.1.1.1—If the force balance is broken, calculate the displacements of MTs, spindle poles, and kinetochores to equilibrate the spindle apparatus again, and then update the positions of all elements within the spindle.

2.1.1.2—If the force balance is not broken, maintain the current state and continue the calculation.

2.1.2 If no pair of MT-bound heads of kinesin-5 motor step forward, step backward or detach, or if no

pair of heads of a free kinesin-5 bind to the MT or no pair of detached heads of the MT-bound kinesin-5 bind to MT, maintain the current state and continue the calculation.

2.2) Determine if there is a new kinesin-13 binding to MTs or if a MT-bound kinesin-13 is detached.

2.2.1 If there is a kinesin-13 binding or detachment, update the lists of kinesin-13 relative to that MT. Concretely, if there is a new kinesin-13 motor binding, add the position of the new kinesin-13 to the list characterizing kinesin-13 for that MT. If there is a kinesin-13 detachment, remove the position of the detached kinesin-13 from the list.

2.2.2 If there is no kinesin-13 binding or detachment, maintain the current state and continue the calculation.

2.3) Determine whether there is a kinesin-13 bound at the last binding position at the minus end of each of the four MTs, and if so, determine whether the kinesin-13 depolymerizes the MT.

2.3.1 If the kinesin-13 depolymerizes the MT, calculate the displacements of MTs, spindle poles, and kinetochores to equilibrate the spindle apparatus again, and then update the positions of all elements in the spindle.

2.3.2 If the kinesin-13 does not depolymerize the MT or if there is no kinesin-13 bound at the last binding position at the minus end of the MT, maintain the current state and continue the calculation.

2.4) Determine whether the plus end of each of the four MTs is elongated by one tubulin.

2.4.1 If the plus end of the MT is elongated, calculate the displacements of MTs, spindle poles, and kinetochores to equilibrate the spindle apparatus again, and then update the positions of all elements within the spindle.

2.4.2 If the plus end of the MT is not elongated, maintain the current state and continue the calculation.

2.5) Increase time t by a time step h and continue the simulation.

The simulation process can be briefly described in the following flow chart.

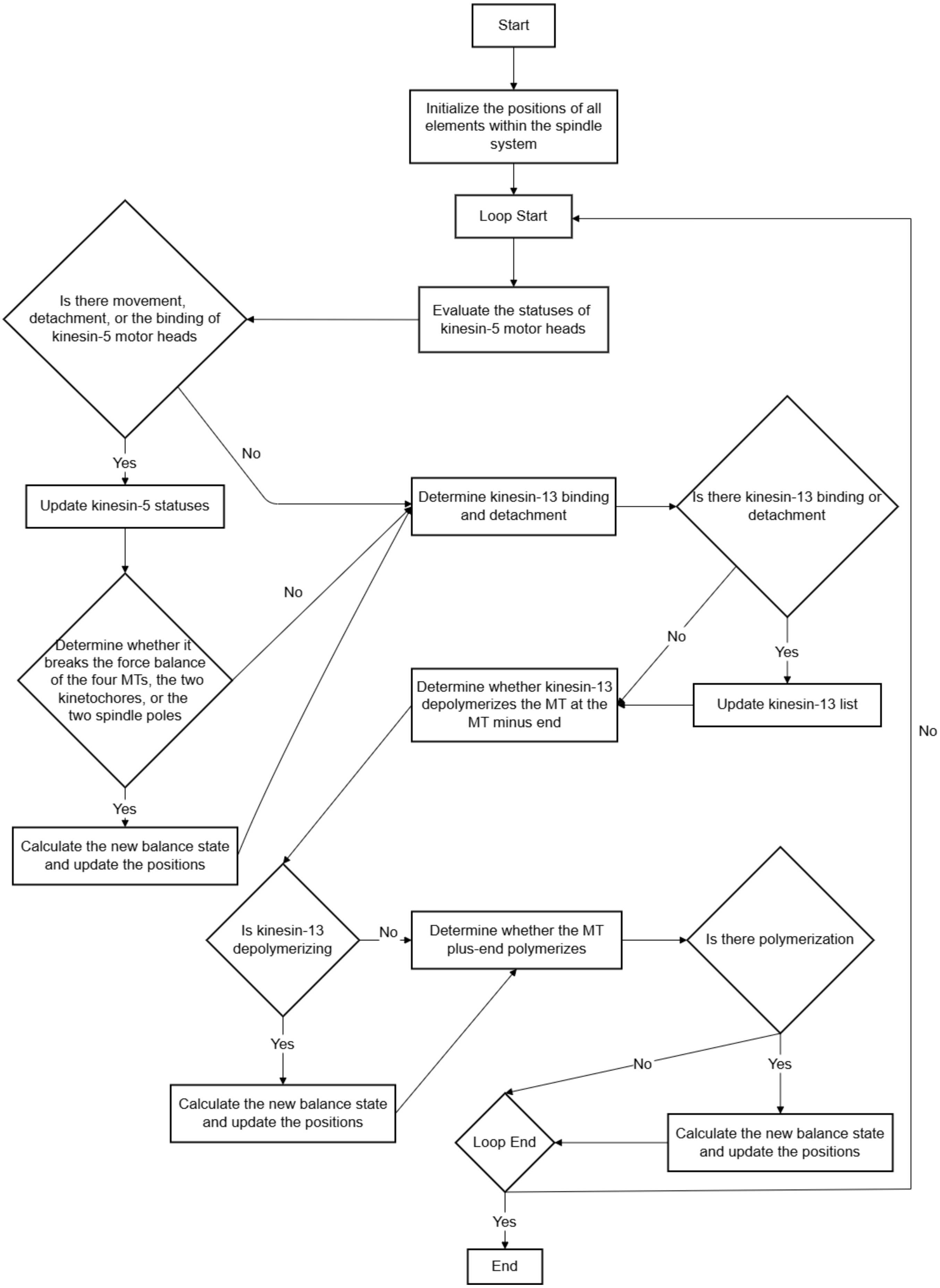

The simulation flow chart.

Methods S5: Method for simulations of spindle system of multiple ensembles of MTs

For multiple ensembles of MTs, there are N pair of iMTs, N pair of kMTs. Taking $N = 2$ as an example, we designate the iMT and kMT connected to the left spindle pole as iMT3 and kMT3, respectively, and the iMT and kMT connected to the right spindle pole as iMT4 and kMT4, respectively. NuMA proteins are located in two parallel overlap regions: one formed by iMT1 and iMT3, and the other one formed by iMT2 and iMT4, which are defined as parallel overlap 1 and parallel overlap 2, respectively. We denote by $\delta_i^{(\gamma)}$ (γ = 1, 2 representing the two parallel overlaps) the ith NuMA's center-of-mass position of the head bound to one MT (iMT1 or iMT2) relative to that of the head bound to another parallel MT (iMT3 or iMT4) along the x direction. The ith NuMA experiences an external elastic force $F_i^{(\text{NuMA})} = K_{\text{NuMA}}\delta_i^{(\gamma)}$. We denote by $N_{\text{NuMA}}^{(\gamma)}(t)$ the total number of the NuMA proteins whose two heads are both bound to MT in the spindle. Owing to the stepping, binding and unbinding of kinesin-5, the binding of NuMA, as well as the polymerization and depolymerization activity of MTs, the spindle system gradually reaches a new equilibrium. We denote the forces acting on the iMT3, iMT4, kMT3 and kMT4 as F_9, F_{10}, F_{11} and F_{12}, respectively (where positive force is in the direction pointing right) after the stepping, binding and unbinding of kinesin-5, or the polymerization and depolymerization activity of MTs. We denote by z_9, z_{10}, z_{11} and z_{12} the movement distances of iMT3, iMT4, kMT3 and kMT4, respectively. On the basis of the force equilibrium equations, Eqs. (S19–S34), it is necessary to include the forces exerted by NuMAs on MTs, and to supplement the force balance equations for iMT3, iMT4, kMT3 and kMT4 at times t and $t+h$, similar to Eqs. (S23–S26) and Eqs. (S31–S34).

Methods S6: Method for simulations of spindle system with branching nucleation

For large cells, both the number of MTs and their polymerization rates within the spindle system vary with the variation of the cell size. We simulate the dynamics of the spindle across different cell sizes, based on the relationship among the polymerization rate v_{p0}, number of smaller MTs attached to each stem MT and cell size, as depicted in Fig. 7B. These smaller MTs, which overlap along the x direction, are connected by nucleation proteins that link the MTs together, facilitating the transfer of forces within the spindle. The distributions of smaller MTs are shown in video S3.

In response to the changed forces acted on the kinetochores, spindle poles and MTs, the displacements of each stem MT and its associated smaller MTs are the same. The displacement of stem iMT1 and its connected smaller MTs is represented by z_5, and that of stem iMT2 and its connected smaller MTs by z_6. The displacement of stem kMT1 and its connected smaller MTs is represented by z_7, and that of stem kMT2 and its connected smaller MTs by z_8. Each MT undergoes polymerization at the plus end and depolymerization at the minus end. Polymerization or depolymerization at ends of MTs that are not directly connected to the spindle poles or kinetochores does not affect the elastic forces throughout the spindle—such as the elastic forces induced by protein stalk stretching, the elastic forces between poles and

MTs, the elastic forces between kinetochores and MTs, and the elastic forces between two kinetochores—and only influences the lengths of the MTs themselves. Apart from the movement of kinesin-5 proteins, the depolymerization at the minus ends directly connected to the poles and the polymerization at the plus ends of kinetochore-associated stem kMTs lead to changes in the forces on the kinetochores, spindle poles and MTs. Using Eqs. (S19-S34), we calculate the displacements of the two spindle poles, two kinetochores, z_1, z_2, z_3, z_4 and the previously defined MTs' displacements z_5, z_6, z_7 and z_8, to equilibrate the spindle apparatus again, thereby updating the positions of all elements in the spindle.

To reproduce quantitatively the experimental data for zebrafish cells, we adjust the values of parameters for kinesin-5 and kinesin-13, with their values being listed in Tables S6 and S7, respectively. The parameters for the polymerization of kMTs at the plus end are still taken as the Second set of values listed in Table S4. The parameters for the three spring elastic constants are still taken as those listed in Table S5.

Methods S7: Calculations of stepping rates of kinesin-5 motor in spindle system

For the spindle system with 1 ensemble of MTs, the stepping rate of a kinesin-5 motor can also be calculated by Eqs. (S3) and (S4), where $\varepsilon_1-\varepsilon_0$ and $\varepsilon_{-1}-\varepsilon_0$ now correspond to the change in the total elastic energy when the moving motor takes one step. The potential energy ε_0 before the moving motor takes the step can be calculated by

$$\begin{aligned}\varepsilon_0 = [&\sum_{j=1}^{N_{\mathrm{E}}^{(1)}} K_{\mathrm{E}}\left(\xi_j^{(1)}\right)^2+\sum_{j=1}^{N_{\mathrm{E}}^{(2)}} K_{\mathrm{E}}\left(\xi_j^{(2)}\right)^2+\sum_{j=1}^{N_{\mathrm{E}}^{(3)}} K_{\mathrm{E}}\left(\xi_j^{(3)}\right)^2+\kappa_1(X_{\mathrm{kinet}}^{(\mathrm{left})}-X_{\mathrm{kinet}}^{(\mathrm{Right})}-d_{\text{K-K}})^2\\ &+\kappa_2(X_{\mathrm{kMT2}}^{(\mathrm{plus})}-X_{\mathrm{kinet}}^{(\mathrm{right})})^2+\kappa_2(X_{\mathrm{kMT1}}^{(\mathrm{plus})}-X_{\mathrm{kinet}}^{(\mathrm{left})})^2+\kappa_3(X_{\mathrm{kMT2}}^{(\mathrm{minus})}-X_{\mathrm{b}}^{(2)})^2+\kappa_3(X_{\mathrm{iMT2}}^{(\mathrm{minus})}-X_{\mathrm{b}}^{(2)})^2\\ &+\kappa_3(X_{\mathrm{iMT1}}^{(\mathrm{minus})}-X_{\mathrm{b}}^{(1)})^2+\kappa_3(X_{\mathrm{kMT1}}^{(\mathrm{minus})}-X_{\mathrm{b}}^{(1)})^2]/2\end{aligned} \quad \text{(S36)}$$

Consider the *i*th motor taking a forward step (towards the MT plus end), which induces the two spindle poles, two kinetochores and four MTs to move the distances that are calculated with the method described in Section S4. We denote by $\xi_i^{(\alpha)}$ the spring extension of the *i*th motor after the movement of the MTs induced by the forward stepping of the *i*th motor in the antiparallel overlap *α*. For the *i*th motor located in the antiparallel overlap 1, if $\Delta x_i^{(1)}+d+z_5-z_6>l_0$, we have $\xi_i^{(1)}=\left(\Delta x_i^{(1)}+d+z_5-z_6-l_0\right)$, where l_0 is the rest distance defined in Section S4. If $\Delta x_i^{(1)}+d+z_5-z_6<-l_0$, we have $\xi_i^{(1)}=\left(\Delta x_i^{(1)}+d+z_5-z_6+l_0\right)$. If $\left|\Delta x_i^{(1)}+d+z_5-z_6\right|<l_0$, we have $\xi_i^{(1)}=0$. If $\Delta x_i^{(2)}+d+z_7-z_6>l_0$, we have $\xi_i^{(2)}=\left(\Delta x_i^{(2)}+d+z_7-z_6-l_0\right)$. For the *i*th motor located in the antiparallel overlap 2, if $\Delta x_i^{(2)}+d+z_7-z_6<-l_0$, we have $\xi_i^{(2)}=\left(\Delta x_i^{(2)}+d+z_7-z_6+l_0\right)$. If $\left|\Delta x_i^{(2)}+d+z_7-z_6\right|<l_0$, we have $\xi_i^{(2)}=0$. For the *i*th motor located in the antiparallel overlap 3, if $\Delta x_i^{(3)}+d+z_8-z_5>l_0$, we have $\xi_i^{(3)}=\left(\Delta x_i^{(3)}+d+z_8-z_5-l_0\right)$. If $\Delta x_i^{(3)}+d+z_8-z_5<-l_0$, we have $\xi_i^{(3)}=\left(\Delta x_i^{(3)}+d+z_8-z_5+l_0\right)$. If $\left|\Delta x_i^{(3)}+d+z_8-z_5\right|<l_0$, we have

$\xi_i^{(3)} = 0$. We denote by $\xi_j^{(\alpha)}$ (j = 1, 2, …, $N_{\rm E}^{(\alpha)}$ and $j \neq i$) the spring extension of the jth motor after the movement of the MTs induced by the forward stepping of the ith motor in the antiparallel overlap α. If $\Delta x_j^{(1)} + z_5 - z_6 > l_0$, we have $\xi_j^{(1)} = \left(\Delta x_j^{(1)} + z_5 - z_6 - l_0\right)$. If $\Delta x_j^{(1)} + z_5 - z_6 < -l_0$, we have $\xi_j^{(1)} = \left(\Delta x_j^{(1)} + z_5 - z_6 + l_0\right)$. If $\left|\Delta x_j^{(1)} + z_5 - z_6\right| < l_0$, we have $\xi_i^{(1)} = 0$. If $\Delta x_j^{(2)} + z_7 - z_6 > l_0$, we have $\xi_j^{(2)} = \left(\Delta x_j^{(2)} + z_7 - z_6 - l_0\right)$. If $\Delta x_j^{(2)} + z_7 - z_6 < -l_0$, we have $\xi_j^{(2)} = \left(\Delta x_j^{(2)} + z_7 - z_6 + l_0\right)$. If $\left|\Delta x_j^{(2)} + z_7 - z_6\right| < l_0$, we have $\xi_j^{(2)} = 0$. If $\Delta x_j^{(3)} + z_8 - z_5 > l_0$, we have $\xi_j^{(3)} = \left(\Delta x_j^{(3)} + z_8 - z_5 - l_0\right)$. If $\Delta x_j^{(3)} + z_8 - z_5 < -l_0$, we have $\xi_j^{(3)} = \left(\Delta x_j^{(3)} + z_8 - z_5 + l_0\right)$. If $\left|\Delta x_j^{(3)} + z_8 - z_5\right| < l_0$, we have $\xi_j^{(3)} = 0$. Then, the potential energy ε_1 in Eqs. (S3) and (S4) for the calculation of the forward stepping rate of the ith motor in the antiparallel overlap α can be calculated by

$$\begin{aligned}\varepsilon_1 = [&\sum_{j=1}^{N_{\rm E}^{(1)}} K_{\rm E}\left(\xi_j^{(1)}\right)^2 + \sum_{j=1}^{N_{\rm E}^{(2)}} K_{\rm E}\left(\xi_j^{(2)}\right)^2 + \sum_{j=1}^{N_{\rm E}^{(3)}} K_{\rm E}\left(\xi_j^{(3)}\right)^2 + \kappa_1(X_{\rm kinet}^{(\rm left)} + z_3 - X_{\rm kinet}^{(\rm Right)} - z_4 - d_{\rm K\text{-}K})^2 \\ &+\kappa_2(X_{\rm kMT2}^{(\rm plus)} + z_8 - X_{\rm kinet}^{(\rm right)} - z_4)^2 + \kappa_2(X_{\rm kMT1}^{(\rm plus)} + z_7 - X_{\rm kinet}^{(\rm left)} - z_3)^2 + \kappa_3(X_{\rm kMT2}^{(\rm minus)} + z_8 - X_{\rm b}^{(2)} - z_2)^2 \\ &+\kappa_3(X_{\rm iMT2}^{(\rm minus)} + z_6 - X_{\rm b}^{(2)} - z_2)^2 + \kappa_3(X_{\rm iMT1}^{(\rm minus)} + z_5 - X_{\rm b}^{(1)} - z_1)^2 + \kappa_3(X_{\rm kMT1}^{(\rm minus)} + z_7 - X_{\rm b}^{(1)} - z_1)^2]/2\end{aligned} \quad \text{(S37)}$$

Consider the ith motor taking a backward step (towards the MT minus end), which induces the two spindle poles, two kinetochores and four MTs to move by the distances that are calculated with the method described in Section S4. Note that the calculated z_1, z_2, z_3, z_4, z_5, z_6, z_7 and z_8 when the ith motor taking a backward step are different from the calculated z_1, z_2, z_3, z_4, z_5, z_6, z_7 and z_8 when the ith motor in the antiparallel overlap α taking a forward step. We still denote by $\xi_i^{(\alpha)}$ the spring extension of the ith motor after the movement of the MTs induced by the backward stepping of the ith motor in the antiparallel overlap α. For the ith motor located in the antiparallel overlap 1, if $\Delta x_i^{(1)} - d + z_5 - z_6 > l_0$, we have $\xi_i^{(1)} = \left(\Delta x_i^{(1)} - d + z_5 - z_6 - l_0\right)$. If $\Delta x_i^{(1)} - d + z_5 - z_6 < -l_0$, we have $\xi_i^{(1)} = \left(\Delta x_i^{(1)} - d + z_5 - z_6 + l_0\right)$. If $\left|\Delta x_i^{(1)} - d + z_5 - z_6\right| < l_0$, we have $\xi_i^{(1)} = 0$. For the ith motor located in the antiparallel overlap 2, if $\Delta x_i^{(2)} - d + z_7 - z_6 > l_0$, we have $\xi_i^{(2)} = \left(\Delta x_i^{(2)} - d + z_7 - z_6 - l_0\right)$. If $\Delta x_i^{(2)} - d + z_7 - z_6 < -l_0$, we have $\xi_i^{(2)} = \left(\Delta x_i^{(2)} - d + z_7 - z_6 + l_0\right)$. If $\left|\Delta x_i^{(2)} - d + z_7 - z_6\right| < l_0$, we have $\xi_i^{(2)} = 0$. For the ith motor located in the antiparallel overlap 3, if $\Delta x_i^{(3)} - d + z_8 - z_5 > l_0$, we have $\xi_i^{(3)} = \left(\Delta x_i^{(3)} - d + z_8 - z_5 - l_0\right)$. If $\Delta x_i^{(3)} - d + z_8 - z_5 < -l_0$, we have $\xi_i^{(3)} = \left(\Delta x_i^{(3)} - d + z_8 - z_5 + l_0\right)$. If $\left|\Delta x_i^{(3)} - d + z_8 - z_5\right| < l_0$, we have $\xi_i^{(3)} = 0$. We denote by $\xi_j^{(\alpha)}$ (j = 1, 2, …, $N_{\rm E}^{(\alpha)}$ and $j \neq i$) the spring extension of the jth motor after the movement of the MTs induced by the backward stepping of the ith motor. If $\Delta x_j^{(1)} + z_5 - z_6 > l_0$, we have $\xi_j^{(1)} = \left(\Delta x_j^{(1)} + z_5 - z_6 - l_0\right)$. If $\Delta x_j^{(1)} + z_5 - z_6 < -l_0$, we have $\xi_j^{(1)} = \left(\Delta x_j^{(1)} + z_5 - z_6 + l_0\right)$. If $\left|\Delta x_j^{(1)} + z_5 - z_6\right| < l_0$,

we have $\xi_i^{(1)}=0$. If $\Delta x_j^{(2)}+z_7-z_6>l_0$, we have $\xi_j^{(2)}=\left(\Delta x_j^{(2)}+z_7-z_6-l_0\right)$. If $\Delta x_j^{(2)}+z_7-z_6<-l_0$, we have $\xi_j^{(2)}=\left(\Delta x_j^{(2)}+z_7-z_6+l_0\right)$. If $\left|\Delta x_j^{(2)}+z_7-z_6\right|<l_0$, we have $\xi_j^{(2)}=0$. If $\Delta x_j^{(3)}+z_8-z_5>l_0$, we have $\xi_j^{(3)}=\left(\Delta x_j^{(3)}+z_8-z_5-l_0\right)$. If $\Delta x_j^{(3)}+z_8-z_5<-l_0$, we have $\xi_j^{(3)}=\left(\Delta x_j^{(3)}+z_8-z_5+l_0\right)$. If $\left|\Delta x_j^{(3)}+z_8-z_5\right|<l_0$, we have $\xi_j^{(3)}=0$. Then, the potential energy ε_{-1} in Eqs. (S3) and (S4) for the calculation of the backward stepping rate of the *i*th motor in the antiparallel overlap *α* can be calculated by

$$\begin{aligned}\varepsilon_{-1}=[&\sum_{j=1}^{N_{\mathrm{E}}^{(1)}}K_{\mathrm{E}}\left(\xi_j^{(1)}\right)^2+\sum_{j=1}^{N_{\mathrm{E}}^{(2)}}K_{\mathrm{E}}\left(\xi_j^{(2)}\right)^2+\sum_{j=1}^{N_{\mathrm{E}}^{(3)}}K_{\mathrm{E}}\left(\xi_j^{(3)}\right)^2+\kappa_1(X_{\mathrm{kinet}}^{(\mathrm{left})}+z_3-X_{\mathrm{kinet}}^{(\mathrm{Right})}-z_4-d_{\mathrm{K\text{-}K}})^2\\&+\kappa_2(X_{\mathrm{kMT2}}^{(\mathrm{plus})}+z_8-X_{\mathrm{kinet}}^{(\mathrm{right})}-z_4)^2+\kappa_2(X_{\mathrm{kMT1}}^{(\mathrm{plus})}+z_7-X_{\mathrm{kinet}}^{(\mathrm{left})}-z_3)^2+\kappa_3(X_{\mathrm{kMT2}}^{(\mathrm{minus})}+z_8-X_{\mathrm{b}}^{(2)}-z_2)^2\\&+\kappa_3(X_{\mathrm{iMT2}}^{(\mathrm{minus})}+z_6-X_{\mathrm{b}}^{(2)}-z_2)^2+\kappa_3(X_{\mathrm{iMT1}}^{(\mathrm{minus})}+z_5-X_{\mathrm{b}}^{(1)}-z_1)^2+\kappa_3(X_{\mathrm{kMT1}}^{(\mathrm{minus})}+z_7-X_{\mathrm{b}}^{(1)}-z_1)^2]/2\end{aligned}\quad . \tag{S38}$$

For the spindle system with *N* ensembles of MTs, we need to supplement two terms in Eqs. (S36–S38) for the calculation of potential energy ε_0, ε_1 and ε_{-1}. The first term is the sum of potential energy of all NuMA proteins in the 2(*N*–1) parallel overlap regions, and the second term is the sum of potential energy of kinesin-5 proteins in 3(*N*–1) anti-parallel overlap regions. For example, for the 2 ensembles of MTs, the first supplemented term can be written as $K_{\mathrm{NuMA}}\left[\sum_{i=1}^{N_{NuMA}^{(1)}}\left(\delta_i^{(1)}\right)^2+\sum_{i=1}^{N_{NuMA}^{(2)}}\left(\delta_i^{(2)}\right)^2\right]\Big/2$ and the second supplemented term can be written as $K_{\mathrm{E}}\left[\sum_{j=1}^{N_{\mathrm{E}}^{(4)}}\left(\xi_j^{(4)}\right)^2+\sum_{j=1}^{N_{\mathrm{E}}^{(5)}}\left(\xi_j^{(5)}\right)^2+\sum_{j=1}^{N_{\mathrm{E}}^{(6)}}\left(\xi_j^{(6)}\right)^2\right]\Big/2$.

Methods S8: Kinesin-13 depolymerizing MTs from the minus end in a length-dependent manner

To investigate the dependences of the MT depolymerization rate v_{dep} by kinesin-13 motors on the MT length *L* and MT sliding rate v_{slide}, we consider a system composed of only a single MT of fixed length *L* and kinesin-13 motors. The MT moves toward the minus end with velocity v_{slide}. Kinesin-13 can bind to a MT, detach from the MT, diffuse on the MT and depolymerize the MT after the motor reaching the minus end. To maintain a constant MT length *L*, it is required that when one tubulin is depolymerized at the MT minus end one tubulin is added at the MT plus end.

The configuration of kinesin-13s on the MT can be described by tubulin occupation number n_{i}, where subscript *i* is the number of the tubulin from the MT minus end, with $n_{\mathrm{i}}=0$ representing the unoccupied tubulin by kinesin-13, $n_{\mathrm{i}}=1$ representing the occupied tubulin by kinesin-13 and MT length $L=id$. Thus, the average depolymerization rate v_{dep} is proportional to the average occupation of the nearest tubulin from the minus end, $\langle n_1\rangle$, with the depolymerization rate $v_{\mathrm{dep}}=k_{\mathrm{dep}}d\langle n_1\rangle$.

We use the Monte-Carlo simulation procedure for kinesin-13, as described in Section S3, and the Second set of values for parameters of kinesin-13 listed in Table S2 to calculate the MT depolymerization rate v_{dep}. The results are shown in the Fig. S4. From Fig. S4 it is seen that the MT depolymerization rate is insensitive to the MT-sliding rate but increases with the increase of the MT length until reaching the maximal value of $v_{dep}^{(max)} = k_{dep}d$.

Methods S9: Effects of elastic constants on dynamics of spindle

In this section, we study the effects of the three spring elastic constants κ_1, κ_2 and κ_3 on the spindle dynamics. We use the preset value v_{p0} = 24 nm/s and the Second set of values to make the simulation. We note that in the wide range of values for each elastic constant, both the overlap length and spindle length change slightly (upper panels of Fig. S9A – C and Fig. S10 A – C), implying that the stationary overlap length and spindle length are insensitive to the spring elastic constants. Except at very small κ_3 (e.g., κ_3 = 0.01 pN/nm), in the wide range of values for each elastic constant, the kMT flux rates also have the similar values (lower panels of Fig. S9 A – C and Fig. S10 A – C).

Supplemental Tables

Table S1. Parameter values of kinesin-5 Eg5 motor for human cells

Parameter	The First set of values	The Second set of values	Source
$k^{(+)}$ (s^{-1})	12.8	25.6	[S2,S10]
$k^{(-)}$ (s^{-1})	$k^{(+)}/15$	$k^{(+)}/15$	[S2]
E_D (k_BT)	2.5	5	[S2]
k_{NL} (s^{-1})	200	400	[S2]
k_D (s^{-1})	50	100	[S2,S11]
k_r (s^{-1})	4.6	9.2	[S2]
δ_w (nm)	1		[S2]
ε_{w0} (s^{-1})	5		[S2]
δ_s (nm)	2.3		Estimated
ε_{s0} (s^{-1})	0.1		[S6]
μ_m (s^{-1})	0.2		[S1]
$k_{on0}^{(m)}$ ($nM^{-1}s^{-1}site^{-1}$)	4×10^{-4}		[S1]
K_E (pN/nm)	0.55		[S1]
[K5] (nM)	3		Estimated

$k^{(+)}$ is the rate of ATP transition to ADP in the head with the forward NL orientation (e.g., the trailing head), $k^{(-)}$ is the rate of ATP transition to ADP in the head without the forward NL orientation (e.g., the leading head), E_D is the energy change associated with the conformational change of the head and NL docking induced by ATP binding, k_{NL} is the rate of NL docking, k_D is the rate of ADP release from the head bound to MT, k_r is the rate of the tail domain releasing from the nucleotide-free head, ε_{w0} is the dissociation rate of the motor under no load during the weak-MT binding state with affinity E_{w2}, δ_w is the load-sensitivity distance for the dissociation during the weak-MT binding state, ε_{s0} is the dissociation rate of the motor under no load during the strong-MT binding state, δ_s is the load-sensitivity distance for the dissociation during the strong-MT binding state, μ_m is binding rate of one pair of the heads to one MT when another pair of the heads at the opposite end of the stalk are attached to another MT in the antiparallel overlap zone, $k_{on0}^{(m)}$ is the second-order MT-binding rate of the kinesin-5 motor in solution, [K5] is kinesin-5 concentration, and K_E is the elastic constant of the motor's stalk. Values of $k^{(+)}$, $k^{(-)}$, E_D, k_{NL}, k_D, k_r, ε_{w0} and δ_w for the First set of values were from Ref. [S2] by fitting to the available experimental data [S10,S11] while values of $k^{(+)}$, $k^{(-)}$, E_D, k_{NL}, k_D and k_r for the Second set of values are taken to be two times of those for

the First set of values.

Table S2. Parameter values of kinesin-13 MCAK motor for human cells

Parameter	The First set of values	The Second set of values	Source
$k_{\text{dep}}^{(+)}$ (s^{-1})	5		[S14]
$k_{\text{dep}}^{(-)}$ (s^{-1})	4		[S14]
τ_{end} (s)	2		[S15]
$k_{\text{diff}}^{(\text{K13})}$ (s^{-1})	82		[S13]
[K13] (nM)	1		Estimated
$k_{\text{on0}}^{(\text{K13})}$ ($nM^{-1}s^{-1}nm^{-1}$)	1×10^{-4}	3×10^{-4}	[S12]
$k_{\text{off}}^{(\text{K13})}$ (s^{-1})	1×10^{-3}	3×10^{-3}	Estimated

$k_{\text{dep}}^{(+)}$ and $k_{\text{dep}}^{(-)}$ are depolymeriztion rates of the kinesin-13 motor at the minus end under the pushing and pulling forces, respectively, on the minus end, τ_{end} is the MT-end residence time of the kinesin-13 motor, $k_{\text{diff}}^{(\text{K13})}$ is the forward or backward stepping rate of kinesin-13 on MT due to diffusion, $k_{\text{off}}^{(\text{K13})}$ is the detachment rate of kinesin-13 from MT, $k_{\text{on}}^{(\text{K13})}$ is the second-order binding rate of kinesin-13 to MT, [K13] is kinesin-13 concentration, with $k_{\text{on}}^{(\text{K13})} = k_{\text{on}}^{(\text{K13})}[\text{K13}]$ corresponding to the binding rate of kinesin-13 to MT.

Table S3. Parameter values of NuMA protein

Parameter	Value	Source
$k_{\text{on0}}^{(\text{NuMA})}$ (nM^{-1} $s^{-1}site^{-1}$)	3×10^{-4}	Estimated
[NuMA] (nM)	1	Estimated
K_{NuMA} (pN/nm)	0.03	[S22]
μ_{NuMA} (s^{-1})	1	Estimated

$k_{\text{on0}}^{(\text{NuMA})}$ is the second-order binding rate to MT, [NuMA] is the NuMA concentration, μ_{NuMA} is the binding rate of one head to MT when another head at the opposite end of the stalk is attached to another parallel MT, and K_{NuMA} is the elastic constant of the NuMA stalk. The parameter values are the same for both the First set of values and the Second set of values.

Table S4. Parameter values related to the polymerization of kMT from the plus end

Parameter		The First set of values	The Second set of values	Source
F_{p0} (pN)	For multiple ensembles of MTs	3.2	1.85	Fitted
	For 1 ensemble of MTs	4.1	2.05	Fitted
B		4		Estimated

F_{p0} is the force-sensitivity parameter for the polymerization at the kMT plus end and B is a factor characterizing the reduction of the unloaded polymerization velocity at the kMT plus end relative to that at the free iMT plus end.

Table S5. Parameter values of spring elastic constants

Parameter	Parameter description	Value	Source
κ_1 (pN/nm)	Elastic constant of spring connecting two kinetochores	10	[S23-S25]
κ_2 (pN/nm)	Elastic constant of spring connecting kinetochore and plus end of each kMT	0.1	[S26, S27]
κ_3 (pN/nm)	Elastic constant of spring connecting pole and minus end of each MT	0.1	[S28]

Parameter values are the same for both the First set of values and the Second set of values.

Table S6. Parameter values of kinesin-5 motor for zebrafish cells

Parameter	Values
$k^{(+)}$ (s^{-1})	25.6×9
$k^{(-)}$ (s^{-1})	$k^{(+)}$/100
E_D ($k_B T$)	5
k_{NL} (s^{-1})	400×9
k_D (s^{-1})	100×9
k_r (s^{-1})	9.2×9
δ_w (nm)	1
ε_{w0} (s^{-1})	3
δ_s (nm)	1
ε_{s0} (s^{-1})	0.06

μ_m (s^{-1})	0.2
$k_{on}^{(m)}$ ($nM^{-1}s^{-1}site^{-1}$)	4×10^{-4}
K_E (pN/nm)	0.55
[K5] (nM)	3

The parameter values were taken to fit the experimental data of Rieckhoff et al. [S30] based on those for the Second set of values listed in Table S1.

Table S7. Parameter values of kinesin-13 motor for zebrafish cells

Parameter	Values
$k_{dep}^{(+)}$ (s^{-1})	90
$k_{dep}^{(-)}$ (s^{-1})	90
τ_{end} (s)	0.3
$k_{diff}^{(K13)}$ (s^{-1})	82
[K13] (nM)	1
$k_{on0}^{(K13)}$ ($nM^{-1}s^{-1}nm^{-1}$)	3×10^{-3}
$k_{off}^{(K13)}$ (s^{-1})	3×10^{-3}

The parameter values were taken to fit the experimental data of Rieckhoff et al. [S30] based on those for the Second set of values listed in Table S2.

Supplemental Figures

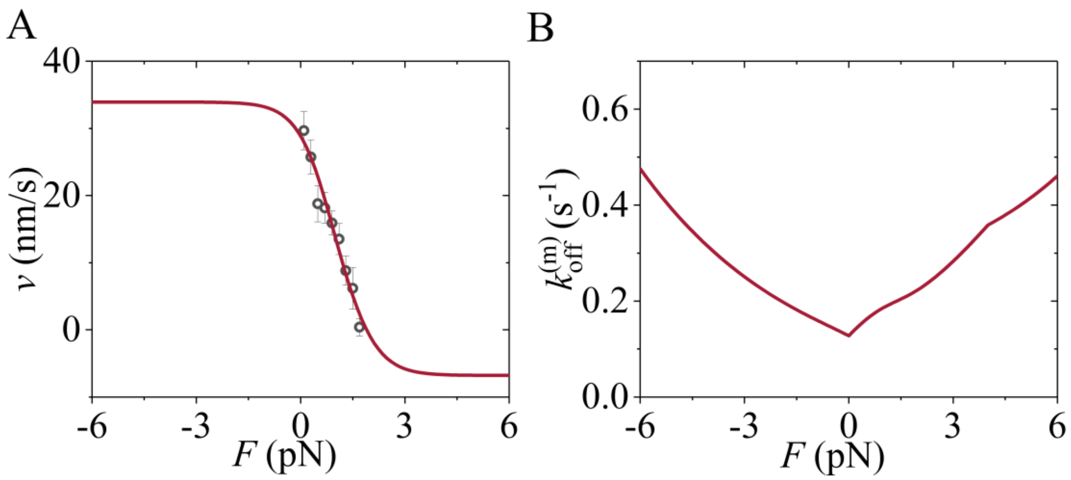

Figure S1: Results for the single full-length Eg5 motor moving on the single MT under load F at saturating ATP concentrations.

(A) Velocity v versus F (line). The positive value of F represents the backward load. Circles are the experimental data measured by Shimamoto et al. at 1-mM ATP concentration [S9].

(B) Dissociation rate $k_{\mathrm{off}}^{(\mathrm{m})}$ versus F.

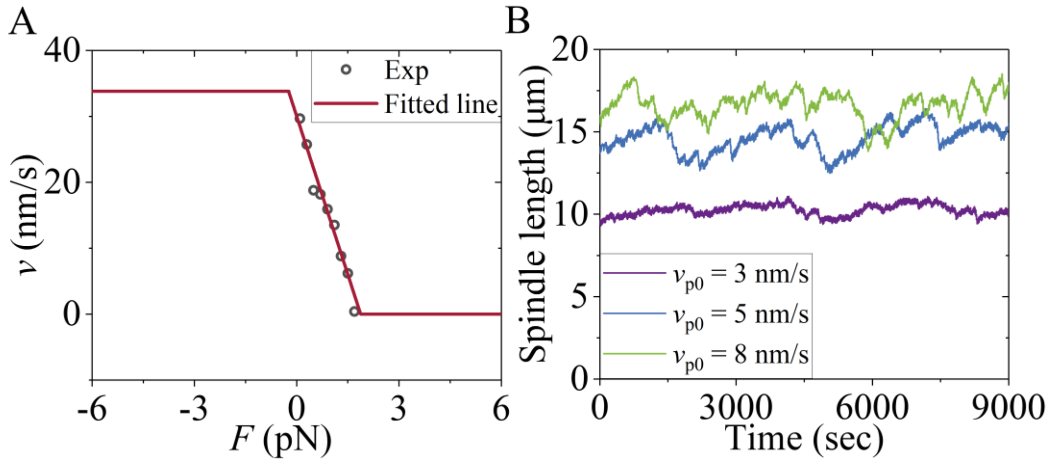

Figure S2: Spindle dynamics simulated using the simple linear form of velocity versus force for kinesin-5.

(A) Linear form of velocity v versus F for the single full-length Eg5 motor moving on the single MT under load F at saturating ATP concentrations (line). The experimental data (dots) are from Shimamoto et al. [S9].

(B) Temporal evolution of the overlap length and spindle length for different values of v_{p0}. The simulations are done for 2 ensembles of MTs using the First set of values for kinesin-5 motor given in Table S1, the First set of values related to the polymerization of kMT from the plus end given in Table S4 and other parameter values given in Tables S2, S3 and S5.

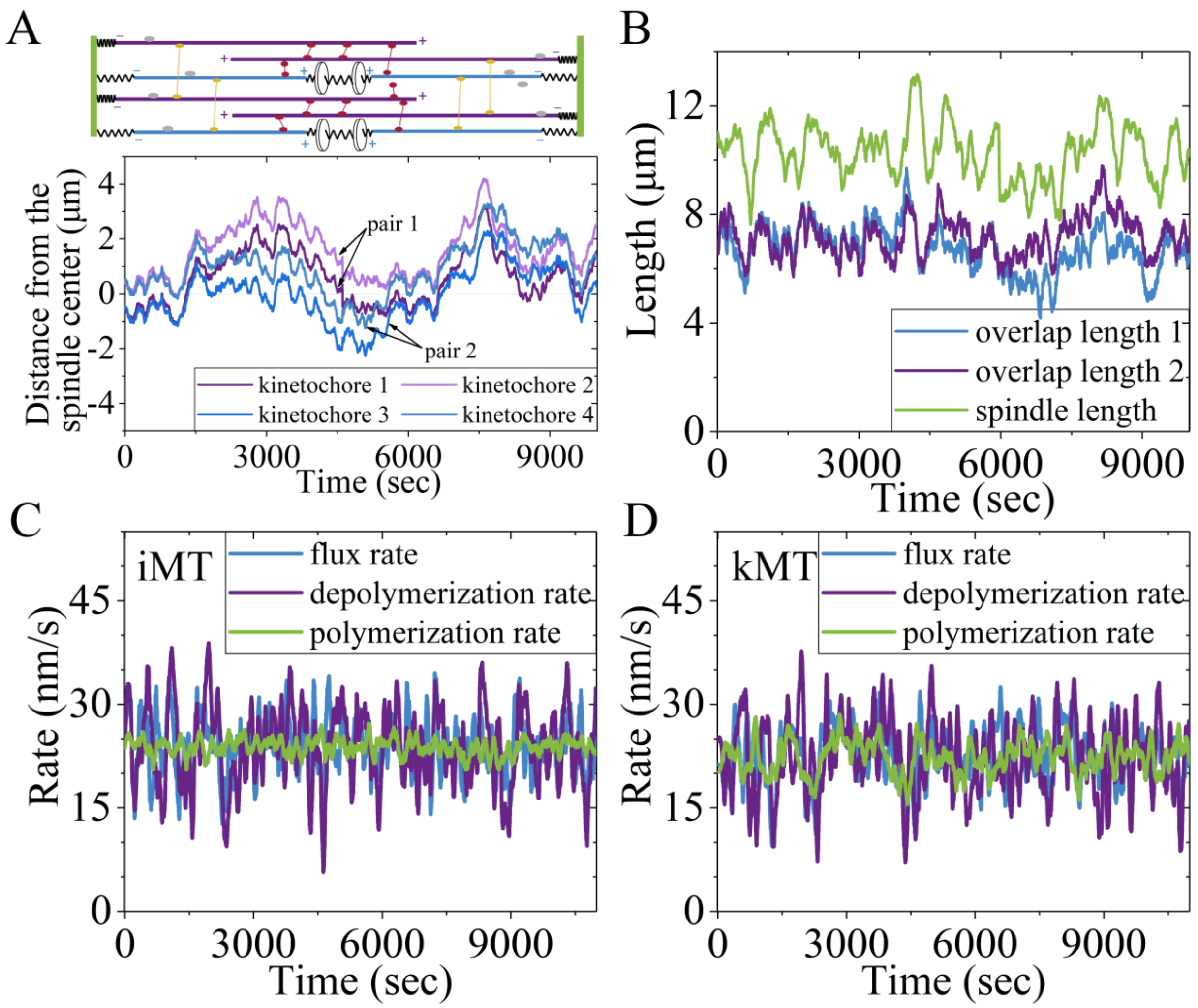

Figure S3: Results for 2 ensembles of MTs with two kinetochore pairs.

The simulations are done with the Second set of values for kinesin-5 motor given in Table S1, the Second set of values related to the polymerization of kMT from the plus end given in Table S4 and other parameter values given in Tables S2, S3 and S5, as well as v_{p0} = 24nm/s, which are the same as those in Fig. 2.

(A) Schematic of the spindle system composed of two pairs of kinetochores (upper panel). Temporal evolution of the positions of two pairs of kinetochores (lower panel).

(B) Temporal evolution of the overlap length and spindle length.

(C, D) Temporal evolution of the flux rate, depolymerization rate and polymerization rate for iMT and kMT. Note that the stationary results shown here are similar to those shown in Fig. 2.

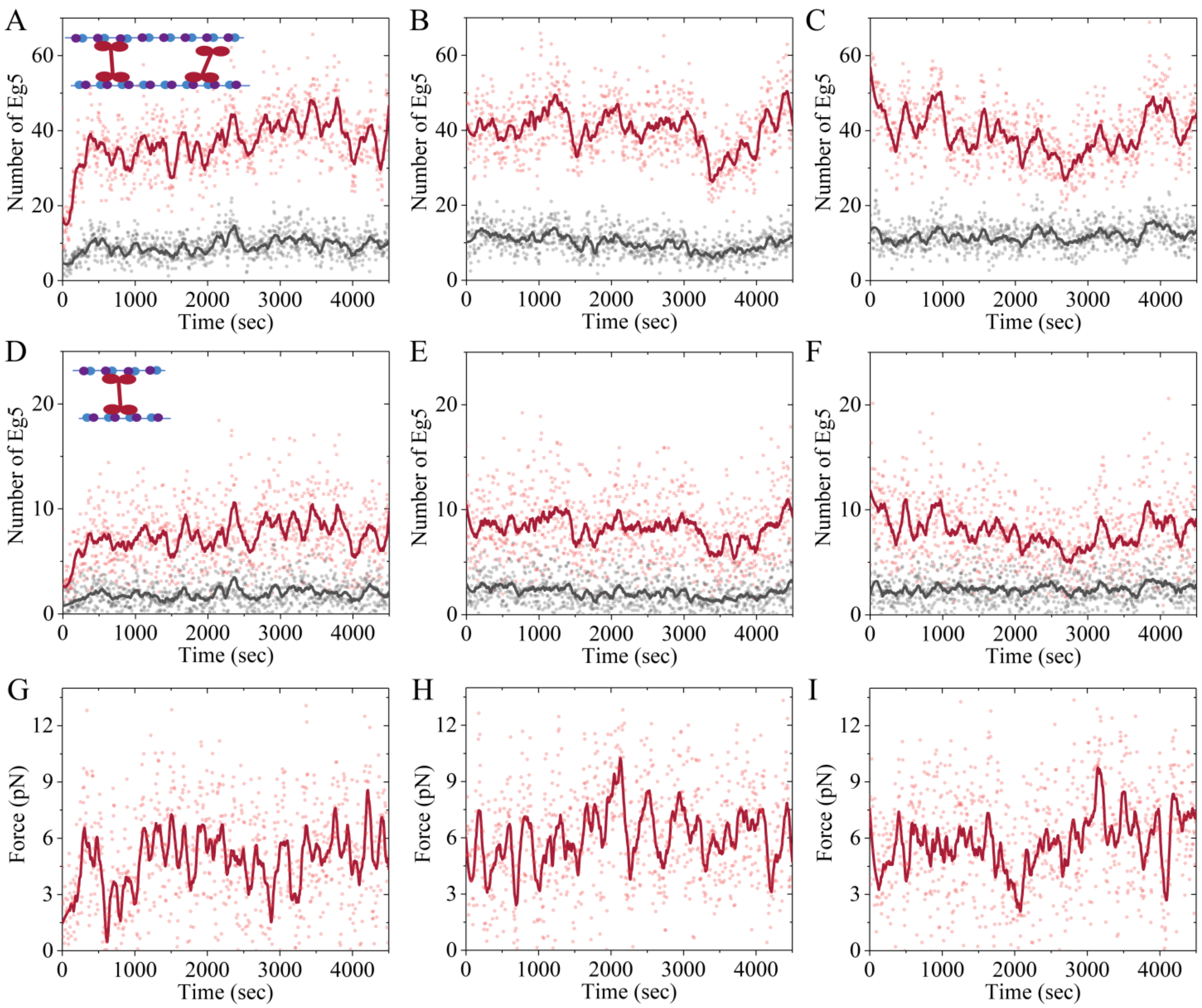

Figure S4: Temporal evolution of the number of kinesin-5 motors in the spindle system for 2 ensembles of MTs.

The results correspond to those in Fig. 2.

(A – C) The total number of kinesin-5s in all overlap zones (red lines and red dots), with black line and black dots representing the number of kinesin-5s in one overlap zone formed by two iMTs.

(D – F) The total number of kinesin-5s with both pairs of heads bound to MTs in all overlap zones (red lines and red dots), with black line and black dots representing the number of kinesin-5s in one overlap zone formed by two iMTs.

(G – I) The pull force on each kMT. The data are smoothed using Savitzky-Golay smoothing method. The data in (A, D, G), (B, E, H) and (C, F, I) are under the same initial conditions as those in Fig. 2A, B and C, respectively. Note that the motor number is directly proportional to the overlap length. Interestingly, from (G, H, I) it is seen that the mean pulling force on each kMT is about 5 pN, which is consistent with the experimental data showing that under the pulling force of about 5 pN the attachment of kMT to kinetochore has the maximum lifetime [S31]. Note that the results here are for $N = 2$ ensembles of MTs (including 2 pairs of antiparallel iMTs and 2 pairs of kMTs) (Fig. 1B). For $N = 3$ ensembles of MTs (including 3 pairs of antiparallel iMTs and 3 pairs of kMTs), with the mean total number of kinesin-5s in the spindle system being

1.5-fold larger than that for $N = 2$, the mean pulling force on each kMT is also about 5 pN (see Fig. S5), indicating that the mean pulling force of about 5 pN on each kMT is independent of N. With the pulling force of about 5 pN on each kMT and the prior electron microscopy data showing that about 30 kMTs are connected to each kinetochore in real spindle system [S32–S34] (corresponding to $N = 30$ ensembles of MTs), with the mean total number of kinesin-5s being 15-fold larger than that for $N = 2$, it is estimated that a force of about 150 pN is present on each kinetochore during metaphase. This force is consistent with the experimental data showing that the chromosome velocity was not affected until the force, which is opposing to that exerted by the spindle during the anaphase, reached about 100 pN and then fell rapidly with increasing force, with the opposing force that caused chromosome velocity to fall to zero being about 700 pN [S35].

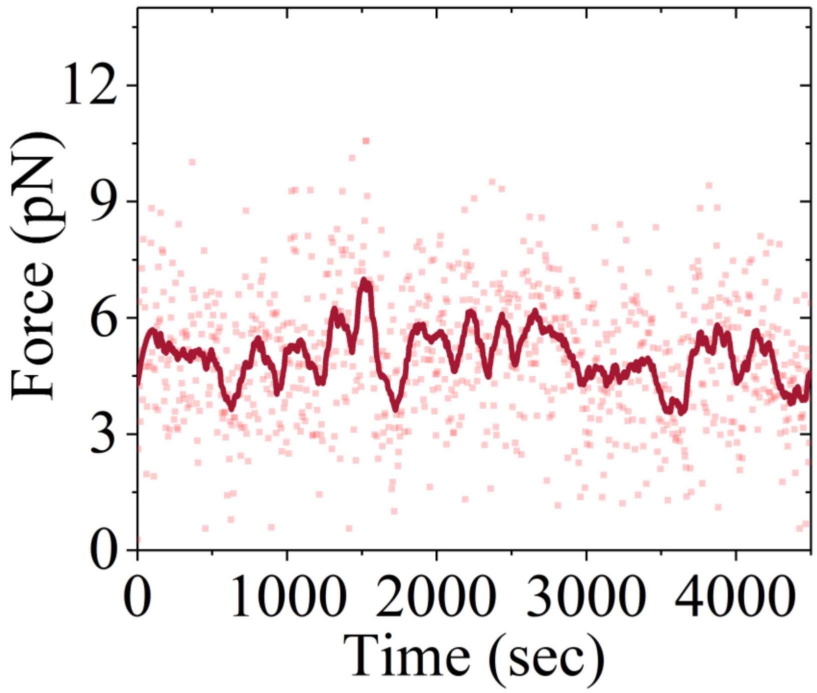

Figure S5: Results for the pulling force on each kMT in the spindle system for $N = 3$ ensembles of MTs at the stationary state.

The data are smoothed using Savitzky-Golay smoothing method. The simulations are done using the same parameter values as those used in Fig. S4. It is seen that the calculated pulling force on each kMT is also about 5 pN, same as that for $N = 2$ ensembles of MTs shown in Fig. S4G – I, indicating that the mean pulling force of about 5 pN on each kMT is independent of N.

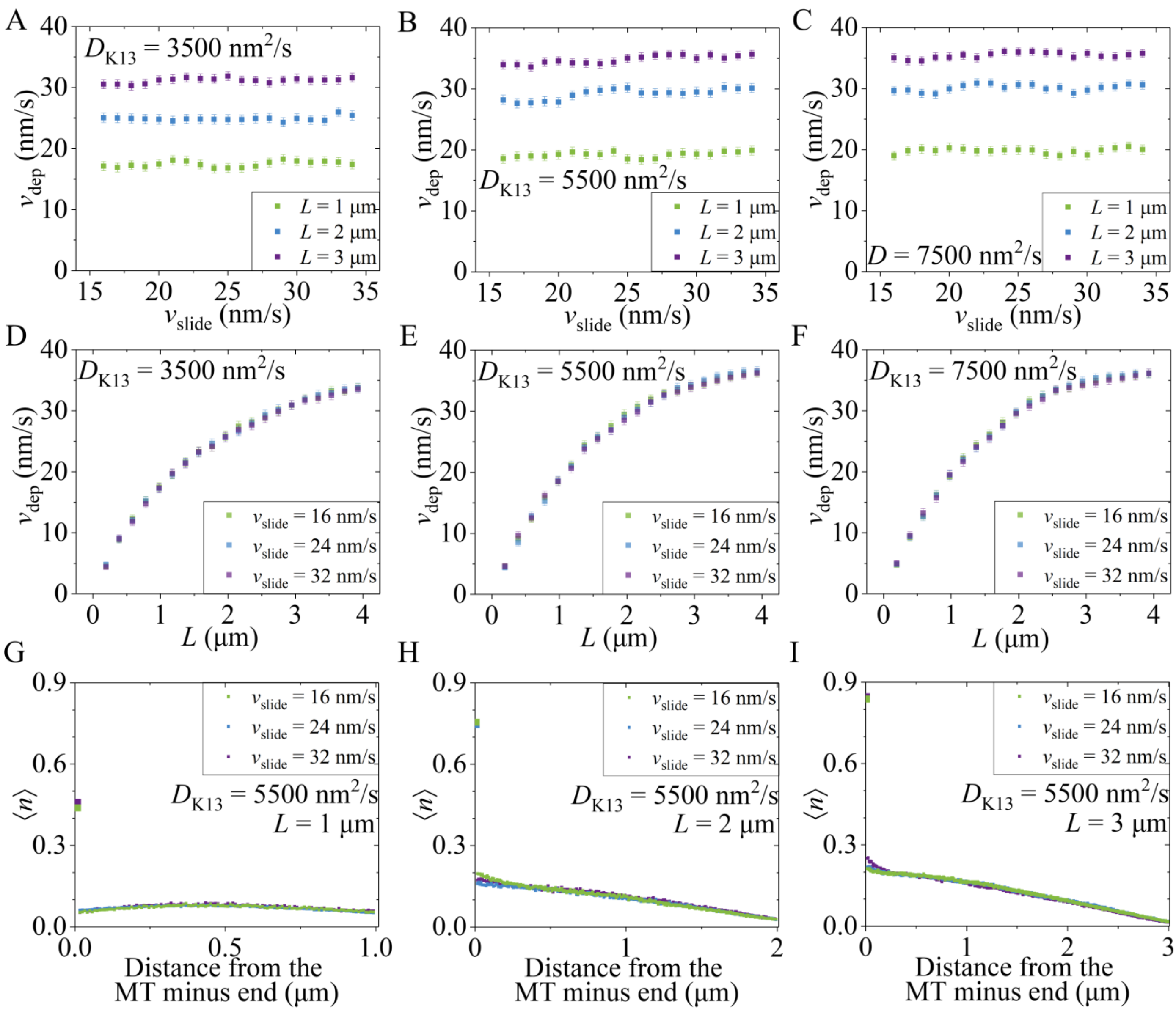

Figure S6: Results for the illustration of kinesin-13s depolymerizing MTs from the minus end in a length-dependent manner.

The simulations are done with k_{dep} = 5 s^{-1} and the Second set of values for parameters of kinesin-13 listed in Table S2.

(A – F) Depolymerization rate at the minus end, v_{dep}, as functions of v_{slide} and L for different values of diffusion constant D_{K13}.

(G – I) Average tubulin occupation $\langle n \rangle$ along the MT for different values of v_{slide} and L.

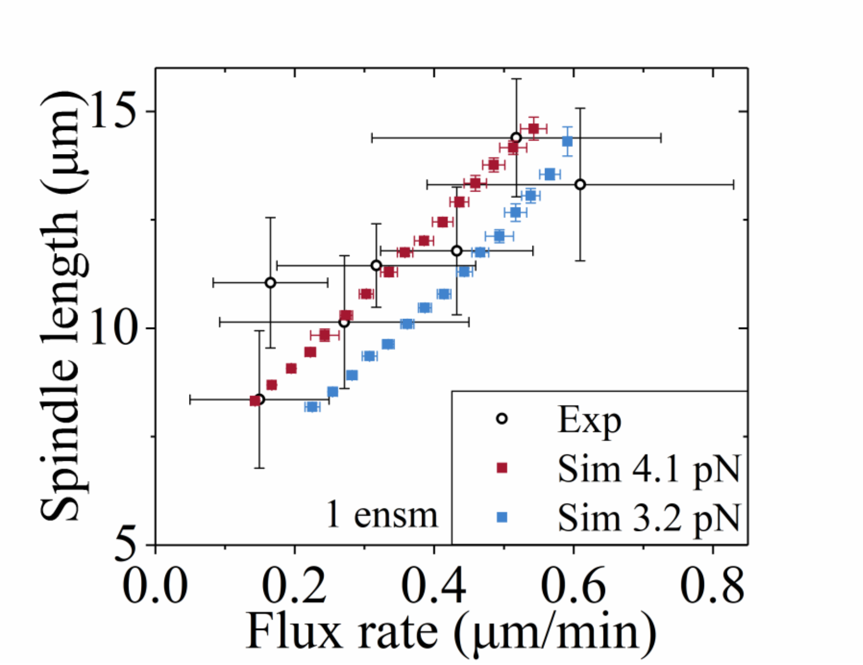

Figure S7: Results of the relationship between the spindle length and flux rate for 1 ensemble of MTs. The simulations are done using the First set of values for kinesin-5 motor given in Table S1, the First set of values related to the polymerization of kMT from the plus end given in Table S4 and other parameter values given in Tables S2, S3 and S5. Red filled squares represent the simulation data (mean ± SEM) with F_{p0} = 4.1 pN. Blue filled squares represent the simulation data (mean ± SEM) with F_{p0} = 3.2 pN. Black hollow circles represent experimental data of Steblyanko et al. [S17].

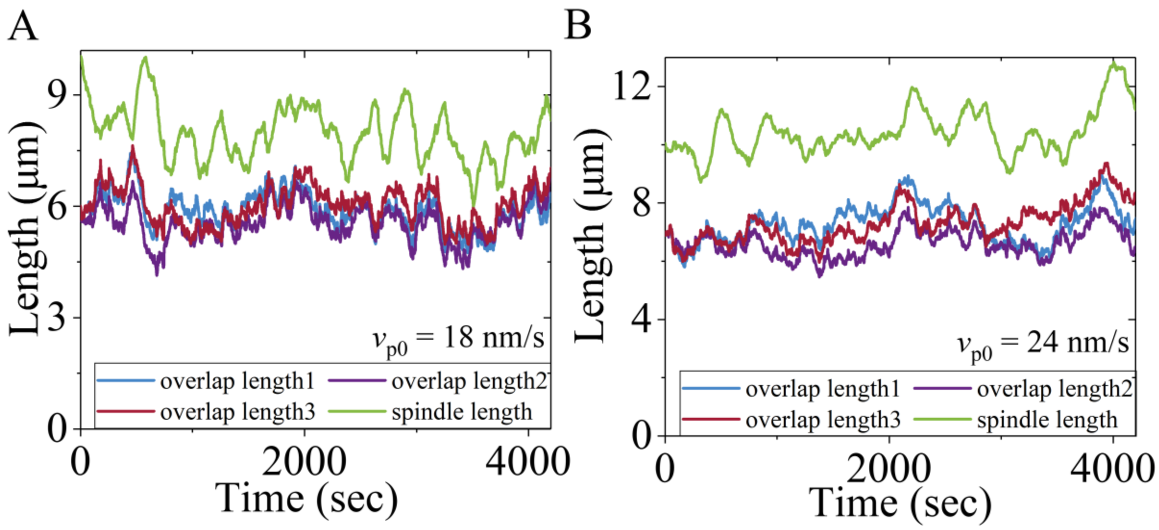

Figure S8: Temporal evolution of the overlap length and spindle length for 3 ensembles of MTs with different values of v_{p0}.

The simulations are done using the Second set of values for kinesin-5 motor given in Table S1, the Second set of values related to the polymerization of kMT from the plus end given in Table S4 and other parameter values given in Tables S2, S3 and S5.

(A) v_{p0} = 18 nm/s.

(B) v_{p0} = 24 nm/s.

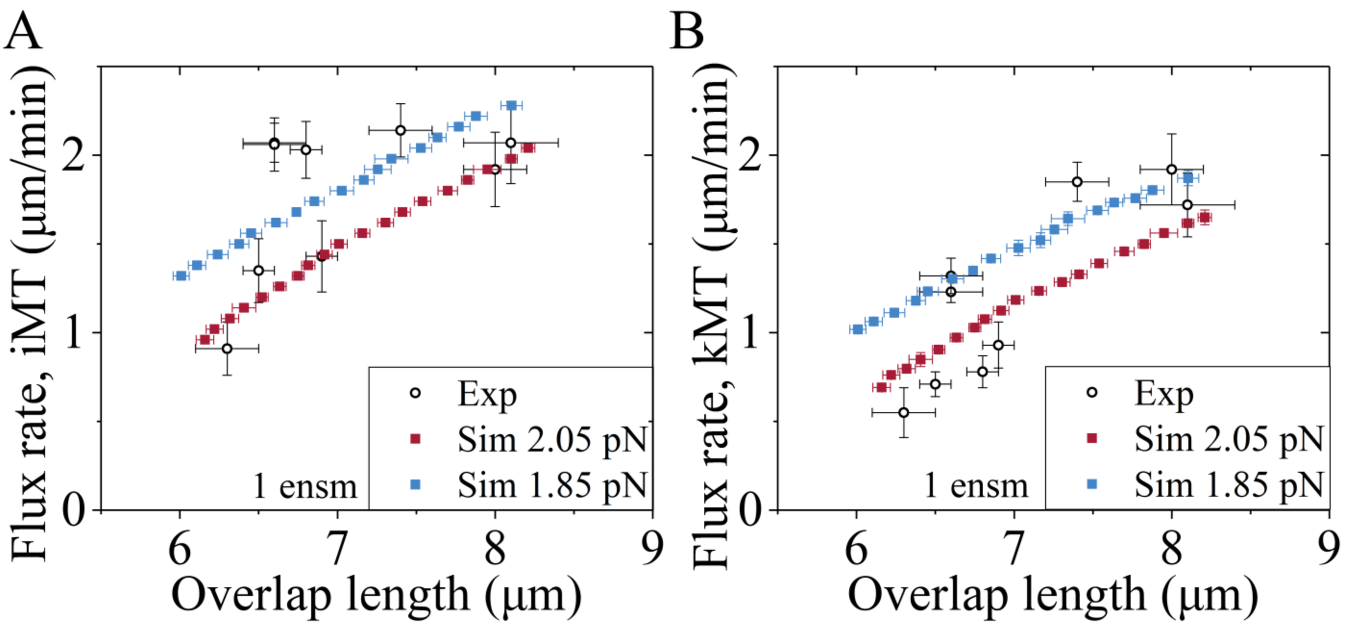

Figure S9: Results of the relationship between the overlap length and flux rate for 1 ensemble of MTs. The simulations are done using the Second set of values for kinesin-5 motor given in Table S1, the Second set of values related to the polymerization of kMT from the plus end given in Table S4 and other parameter values given in Tables S2, S3 and S5. Red filled squares and blue filled squares represent the simulation results (mean ± SEM) with F_{p0} = 2.05 pN and 1.85 pN, respectively. Black hollow circles represent experimental data of Risteski et al. [S36].

(A) Flux rate of iMT versus overlap length.

(B) Flux rate of kMT versus overlap length.

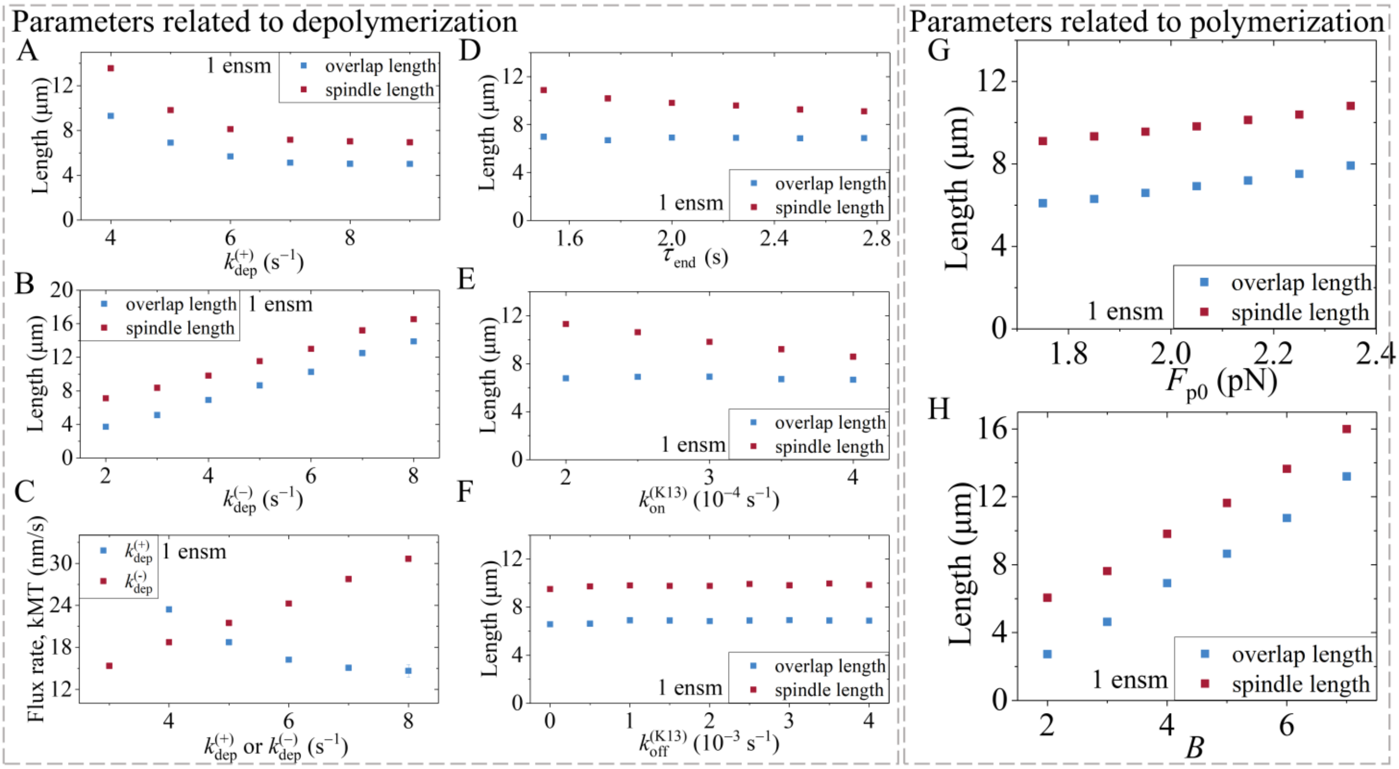

Figure S10: Parameter sensitivity analysis at the stationary state of the spindle for 1 ensemble of MTs.

The simulations are done using the Second set of values for kinesin-5 motor given in Table S1, the Second set of values related to the polymerization of kMT from the plus end given in Table S4 and other parameter values given in Tables S2, S3 and S5, as well as v_{p0} = 24 nm/s.

(A – F) Effects of the variation of each parameter associated with kinesin-13 at the minus end and on the MT lattice on overlap length, spindle length and kMT flux rate.

(G, H) Effects of the variation of each parameter associated with the MT polymerization at the kMT plus end on overlap length and spindle length.

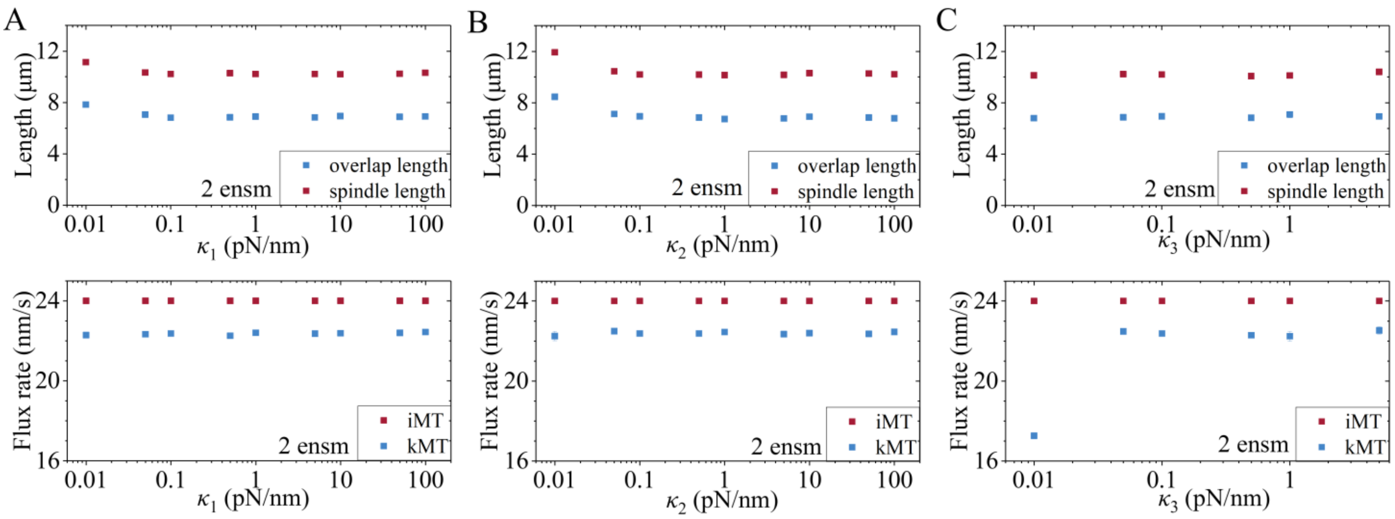

Figure S11: Effects of spring elastic constants on the spindle dynamics for 2 ensembles of MTs.

The simulations are done using the Second set of values for kinesin-5 motor given in Table S1, the Second set of values related to the polymerization of kMT from the plus end given in Table S4 and other parameter values given in Tables S2, S3 and S5, as well as v_{p0} = 24 nm/s.

(A) Effects of the variation of κ_1 on overlap length and spindle length (upper panel) and on kMT flux rate (lower panel).

(B) Effects of the variation of κ_2 on overlap length and spindle length (upper panel) and on kMT flux rate (lower panel).

(C) Effects of the variation of κ_3 on overlap length and spindle length (upper panel) and on kMT flux rate (lower panel).

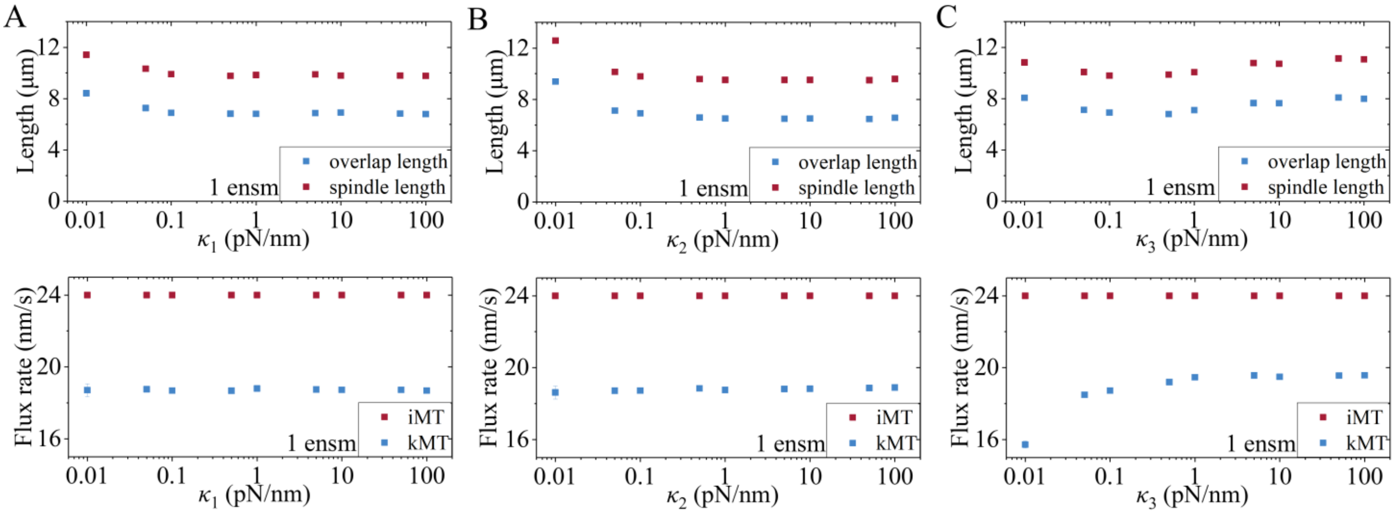

Figure S12: Effects of elastic constants on the dynamics of the spindle for 1 ensemble of MTs.

The simulations are done using the Second set of values for kinesin-5 motor given in Table S1, the Second set of values related to the polymerization of kMT from the plus end given in Table S4 and other parameter values given in Tables S2, S3 and S5, as well as v_{p0} = 24 nm/s.

(A) Effects of the variation of κ_1 on overlap length and spindle length (upper panel) and on kMT flux rate (lower panel).

(B) Effects of the variation of κ_2 on overlap length and spindle length (upper panel) and on kMT flux rate (lower panel).

(C) Effects of the variation of κ_3 on overlap length and spindle length (upper panel) and on kMT flux rate (lower panel).

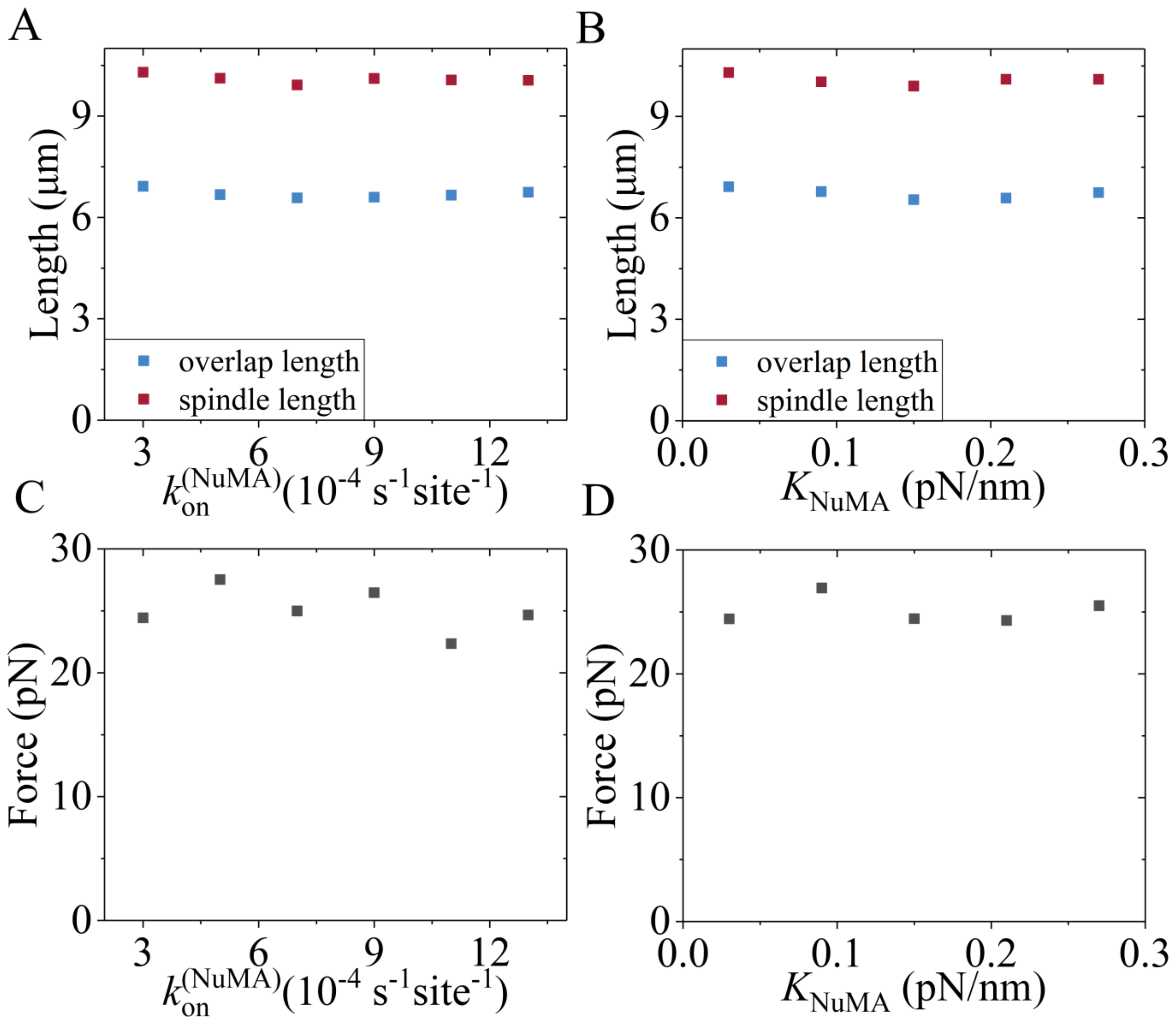

Figure S13: Effects of parameters related to NuMA on the spindle dynamics for 2 ensembles of MTs. The simulations are done using the Second set of values for kinesin-5 motor given in Table S1, the Second set of values related to the polymerization of kMT from the plus end given in Table S4 and other parameter values given in Tables S2, S3 and S5, as well as v_{p0} = 24 nm/s.

(A) The effect of the variation of the binding rate of NuMA to MT in the region of parallel iMTs, $k_{on}^{(NuMA)}$, on the overlap length and spindle length.

(B) The effect of the variation of the elastic constant of the NuMA stalk, K_{NuMA}, on the overlap length and spindle length.

(C) The effect of the variation of $k_{on}^{(NuMA)}$ on the mean total force on an iMT along the plus-end direction, which arises from the stretching of all NuMA stalks.

(D) The effect of the variation of K_{NuMA} on the mean total force on an iMT along the plus-end direction, which arises from the stretching of all NuMA stalks. Note that the magnitude of the mean total force along the minus-end direction is equal to that along the plus-end direction.

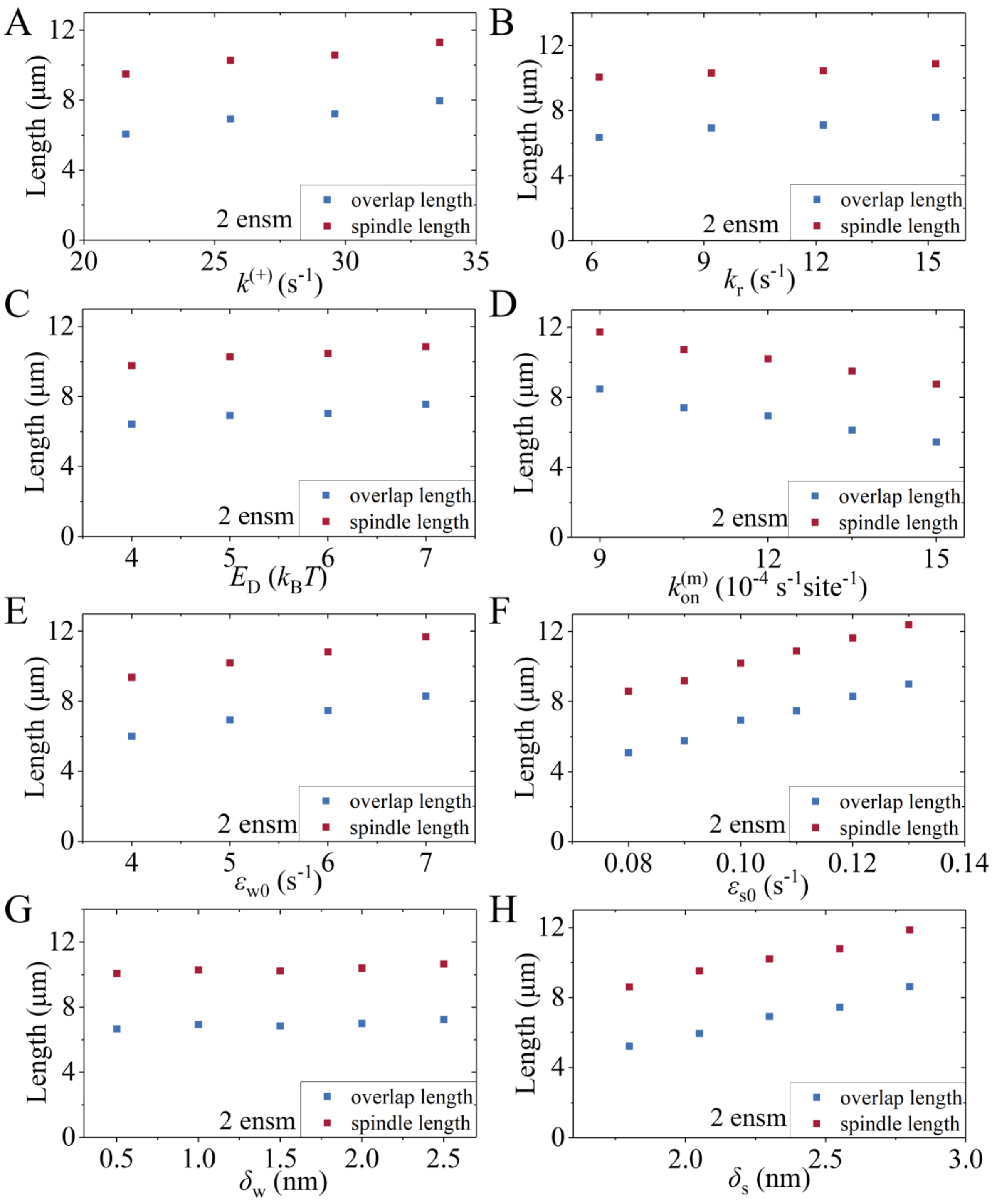

Figure S14: Effects of parameters related to kinesin-5 on the spindle dynamics at the stationary state.

The simulations are done for 2 ensembles of MTs using the Second set of values for kinesin-5 motor given in Table S1, the Second set of values related to the polymerization of kMT from the plus end given in Table S4 and other parameter values given in Tables S2, S3 and S5, as well as v_{p0} = 24 nm/s.

(A – H) Effects of the variation of each parameter on the overlap length and spindle length.

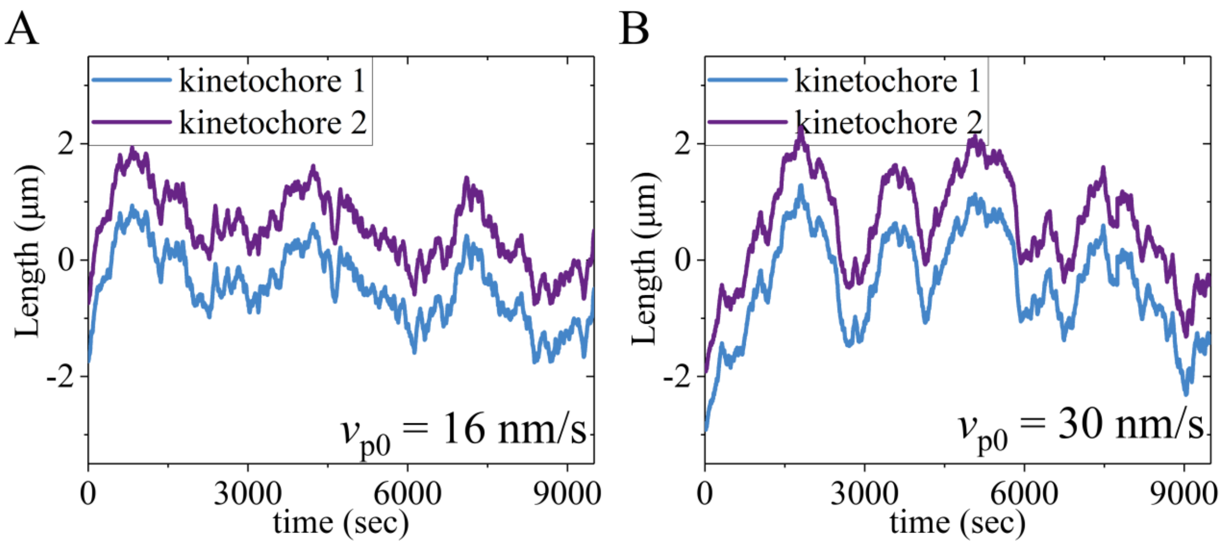

Figure S15: Results for the kinetochore oscillation in the spindle for 2 ensembles of MTs with different values of v_{p0}. The simulations are done using the Second set of values for kinesin-5 motor given in Table S1, the Second set of values related to the polymerization of kMT from the plus end given in Table S4 and other parameter values given in Tables S2, S3 and S5.

(A) v_{p0} = 16 nm/s.

(B) v_{p0} = 30 nm/s. Comparing (A) and (B), it is seen that the oscillation amplitude increases with the increase of v_{p0}.

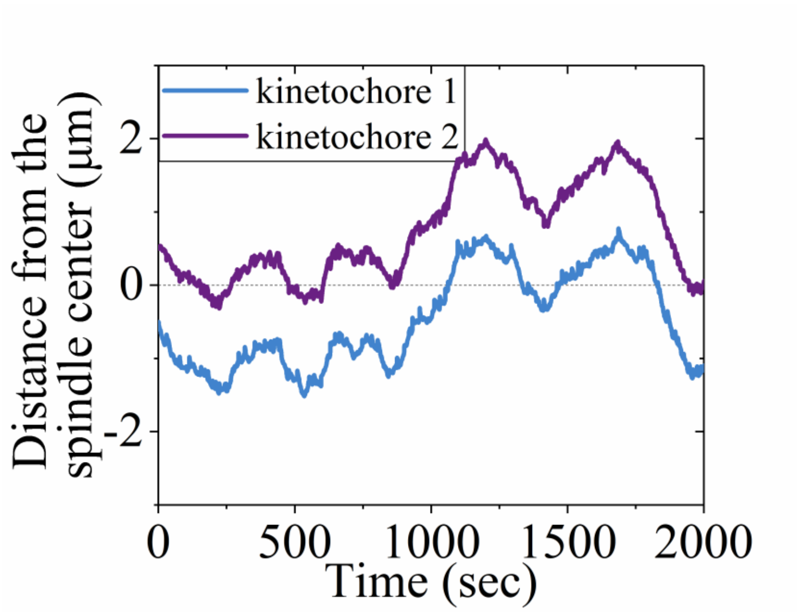

Figure S16: Temporal evolution of the positions of two sister kinetochores in the spindle for 2 ensembles of MTs with v_{p0} = 24 nm/s.

The simulations are done using the Second set of values for kinesin-5 motor given in Table S1, the Second set of values related to the polymerization of kMT from the plus end given in Table S4 and other parameter values given in Tables S2, S3 and S5 except for κ_1 = 0.04 pN/nm. The results show that the two sister kinetochores oscillate with a timescale of about 450 s, which is close to the experimental one [S37].

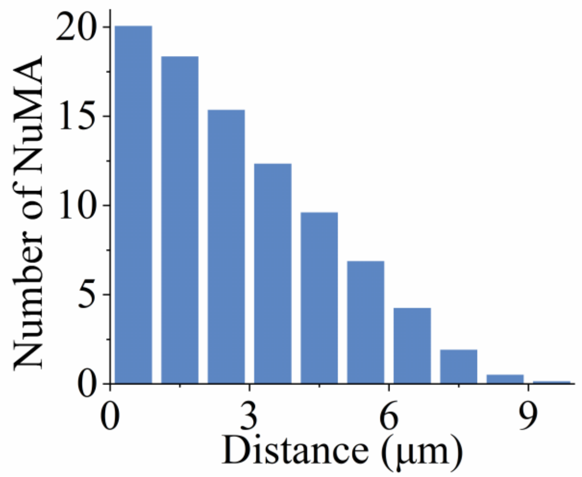

Figure S17: Mean distribution of NuMA numbers in the overlap of two parallel iMTs.

The spindle pole is set as x = 0. The simulations are done with v_{p0} = 24 nm/s, the Second set of values for kinesin-5 motor given in Table S1, the Second set of values related to the polymerization of kMT from the plus end given in Table S4 and other parameter values given in Tables S2, S3 and S5. Due to the poleward flux, the number of NuMAs per μm increases as the distance to the spindle pole decreases.

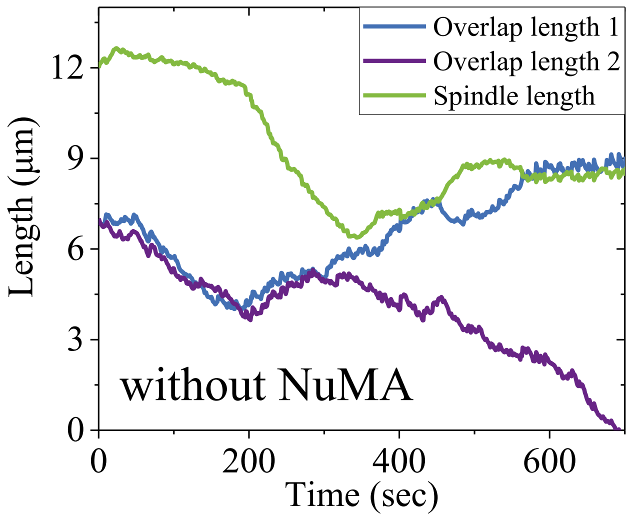

Figure S18: An example of the results for 2 ensembles of MTs without NuMA.

The simulations are done with the Second set of values for kinesin-5 motor given in Table S1, the Second set of values related to the polymerization of kMT from the plus end given in Table S4 and other parameter values given in Tables S2, S3 and S5, as well as v_{p0} = 24 nm/s, which are the same as those in Fig. 2. Without NuMA, the flux rates of different iMT pairs could be very different. Consider the same polymerization rates for all iMs, the overlap zone formed by the iMT pair with a larger flux rate would become narrower and narrower, whereas that with a smaller flux rate would become wider and wider, finally resulting the spindle system to be unstable.

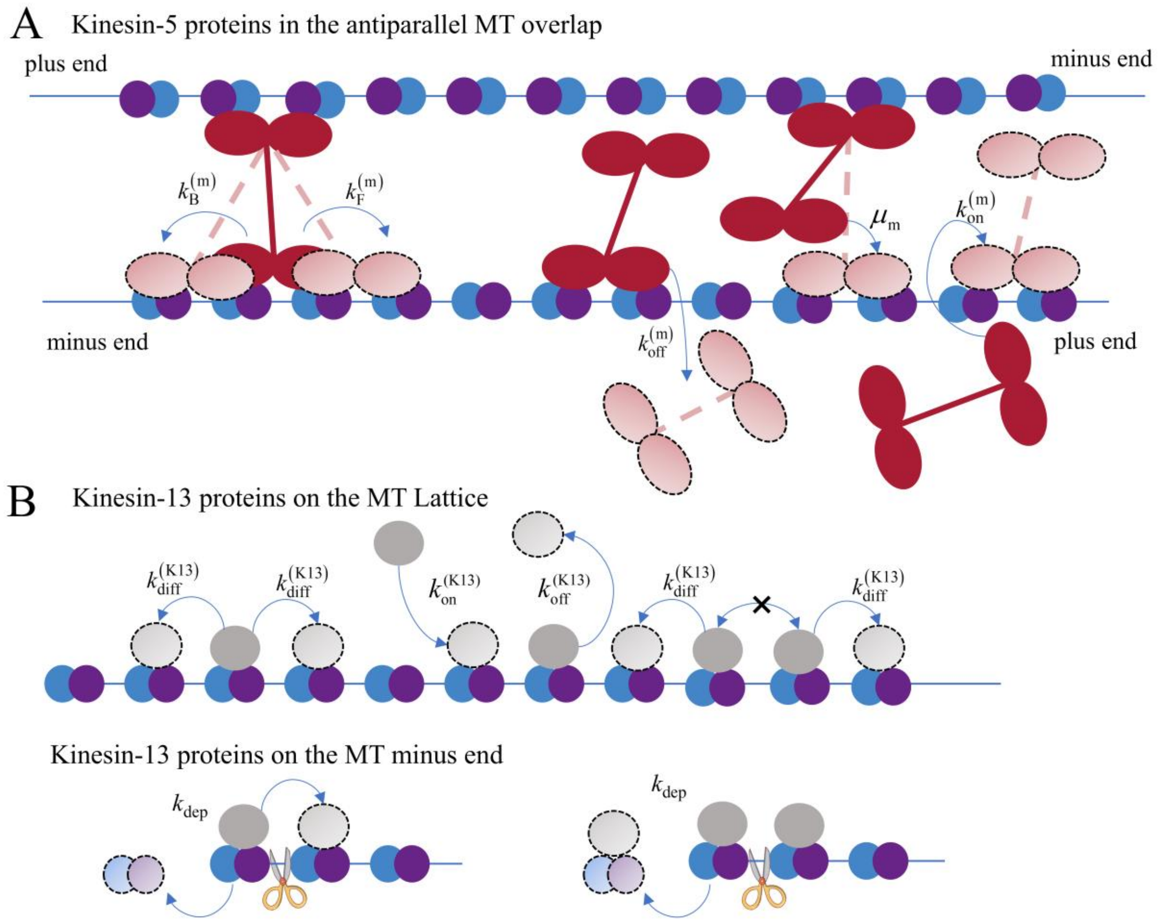

Figure S19: Schematic illustrations of the activities of kinesin-5 and kinesin-13.

(A) Activity of kinesin-5. The dark red ovals represent kinesin-5 heads, while the light red ovals indicate potential positions of the heads at the next moment. The blue and purple circles represent tubulin heterodimers on the MT.

(B) Activity of kinesin-13. The dark gray circles represent kinesin-13 molecules, while the light gray circles indicate their possible positions at the next moment. The blue and purple circles represent tubulin heterodimers on the MT.

SI References

S1. Wang, Y., Liu, Y., Wang, P., and Xie, P. (2024). Computational Studies Reveal How Passive Cross-Linkers Regulate Anaphase Spindle Elongation. J. Phys. Chem. B *128*, 1194-1204.

S2. Liu, Y., Wang, Y., Wang, P., and Xie, P. (2021). Effect of Kinesin-5 Tail Domain on Motor Dynamics for Antiparallel Microtubule Sliding. Int. J. Mol. Sci. *22*, 7857.

S3. Xie, P. (2020). Non-tight and tight chemomechanical couplings of biomolecular motors under hindering loads. J. Theor. Biol. *490*, 110173.

S4. Xie, P., Guo, S., and Chen, H. (2019). ATP-Concentration- and Force-Dependent Chemomechanical Coupling of Kinesin Molecular Motors. J. Chem Inf. Model. *59*, 360-372.

S5. Xie, P., Guo, S.K., and Chen, H. (2019). A Generalized Kinetic Model for Coupling between Stepping and ATP Hydrolysis of Kinesin Molecular Motors. Int. J. Mol. Sci. *20*, 4911.

S6. Xie, P. (2021). Insight into the chemomechanical coupling mechanism of kinesin molecular motors. Commun. Theor. Phys. *73*, 57601.

S7. Xie, P. (2022). Effect of varying load in moving period of a step on dynamics of molecular motors. Eur. Phys. J. E *45*, 28.

S8. Xie, P. (2020). Theoretical Analysis of Dynamics of Kinesin Molecular Motors. ACS Omega *5*, 5721-5730.

S9. Shimamoto, Y., Forth, S., and Kapoor, T.M. (2015). Measuring Pushing and Braking Forces Generated by Ensembles of Kinesin-5 Crosslinking Two Microtubules. Dev. Cell *34*, 669-681.

S10. Valentine, M.T., Fordyce, P.M., Krzysiak, T.C., Gilbert, S.P., and Block, S.M. (2006). Individual dimers of the mitotic kinesin motor Eg5 step processively and support substantial loads in vitro. Nat. Cell Biol. *8*, 470-476.

S11. Valentine, M.T., and Block, S.M. (2009). Force and Premature Binding of ADP Can Regulate the Processivity of Individual Eg5 Dimers. Biophys. J. *97*, 1671-1677.

S12. Cooper, J.R., Wagenbach, M., Asbury, C.L., and Wordeman, L. (2010). Catalysis of the microtubule on-rate is the major parameter regulating the depolymerase activity of MCAK. Nat. Struct. Mol. Biol. *17*, 77-82.

S13. McHugh, T., and Welburn, J.P.I. (2023). Potent microtubule-depolymerizing activity of a mitotic Kif18b–MCAK–EB network. J. Cell Sci. *136*, jcs260144.

S14. Xie, P. (2024). Modeling study of kinesin-13 MCAK microtubule depolymerase. Euro. Biophys. J. *53*, 339–354.

S15. Patel, J.T., Belsham, H.R., Rathbone, A.J., Wickstead, B., Gell, C., and Friel, C.T. (2016). The family-specific alpha4-helix of the kinesin-13, MCAK, is critical to microtubule end recognition. Open Biol. *6*, 160223.

S16. Maffini, S., Maia, A.R.R., Manning, A.L., Maliga, Z., Pereira, A.L., Junqueira, M., Shevchenko, A., Hyman, A., Yates, J.R., and Galjart, N., *et al.* (2009). Motor-Independent Targeting of CLASPs to Kinetochores by CENP-E Promotes Microtubule Turnover and Poleward Flux. Curr. Biol. *19*, 1566-1572.

S17. Steblyanko, Y., Rajendraprasad, G., Osswald, M., Eibes, S., Jacome, A., Geley, S., Pereira, A.J., Maiato, H., and Barisic, M. (2020). Microtubule poleward flux in human cells is driven by the coordinated action of four kinesins. EMBO J. *39*, e105432.

S18. Renda, F., Pellacani, C., Strunov, A., Bucciarelli, E., Naim, V., Bosso, G., Kiseleva, E., Bonaccorsi, S., Sharp, D.J., and Khodjakov, A., *et al.* (2017). The Drosophila orthologue of the INT6 onco-protein regulates mitotic microtubule growth and kinetochore structure. PLOS Genet. *13*, e1006784.

S19. Fernandez, N., Chang, Q., Buster, D.W., Sharp, D.J., and Ma, A. (2009). A Model for the Regulatory Network Controlling the Dynamics of Kinetochore Microtubule Plus-Ends and Poleward Flux in Metaphase. Proc. Natl. Acad. Sci. U.S.A. *106*, 7846-7851.

S20. Wang, H., Brust-Mascher, I., Cheerambathur, D., and Scholey, J.M. (2010). Coupling between microtubule sliding,

plus-end growth and spindle length revealed by kinesin-8 depletion. Cytoskeleton *67*, 715-728.

S21. Reber, S.B., Baumgart, J., Widlund, P.O., Pozniakovsky, A., Howard, J., Hyman, A.A., and Jülicher, F. (2013). XMAP215 activity sets spindle length by controlling the total mass of spindle microtubules. Nat. Cell Biol. *15*, 1116-1122.

S22. Harborth, J., Wang, J., Gueth-Hallonet, C., Weber, K., and Osborn, M. (1999). Self assembly of NuMA: multiarm oligomers as structural units of a nuclear lattice. EMBO J. *18*, 1689-1700.

S23. Klemm, A., Bosilj, A., Tolic, I., and Pavin, N. (2018). Metaphase Kinetochore Movements are Regulated by Kinesin-8 Motors and Microtubule Dynamic Instability. Mol. Biol. Cell *29*, 11.

S24. Bloom, K., and Joglekar, A. (2010). Towards building a chromosome segregation machine. Nature *463*, 446-456.

S25. Iemura, K., Yoshizaki, Y., Kuniyasu, K., and Tanaka, K. (2021). Attenuated Chromosome Oscillation as a Cause of Chromosomal Instability in Cancer Cells. Cancers *13*, 4531.

S26. Schwietert, F., and Kierfeld, J. (2019). Bistability and oscillations in cooperative microtubule and kinetochore dynamics in the mitotic spindle. New J. Phys. *22*, 53008.

S27. Banigan, E.J., Chiou, K.K., Ballister, E.R., Mayo, A.M., Lampson, M.A., and Liu, A.J. (2015). Minimal model for collective kinetochore–microtubule dynamics. Proc. Natl. Acad. Sci. U.S.A. *112*, 12699-12704.

S28. Shimamoto, Y., Maeda, Y.T., Ishiwata, S., Libchaber, A.J., and Kapoor, T.M. (2011). Insights into the micromechanical properties of the metaphase spindle. CELL *145*, 1062-1074.

S29. Wang, Y., Liu, Y., Wang, P., and Xie, P. (2022). Dynamics of cooperative transport by multiple kinesin motors and diffusing microtubule-associated proteins. Commun. Theor. Phys. *74*, 105601.

S30. Rieckhoff, E.M., Berndt, F., Elsner, M., Golfier, S., Decker, F., Ishihara, K., and Brugués, J. (2020). Spindle Scaling Is Governed by Cell Boundary Regulation of Microtubule Nucleation. Curr. Biol. *30*, 4973-4983.

S31. Akiyoshi, B., Sarangapani, K.K., Powers, A.F., Nelson, C.R., Reichow, S.L., Arellano-Santoyo, H., Gonen, T., Ranish, J.A., Asbury, C.L., and Biggins, S. (2010). Tension directly stabilizes reconstituted kinetochore-microtubule attachments. Nature *468*, 576-579.

S32. Banigan, E.J., Chiou, K.K., Ballister, E.R., Mayo, A.M., Lampson, M.A., and Liu, A.J. (2015). Minimal model for collective kinetochore–microtubule dynamics. Proc. Natl. Acad. Sci. U.S.A. *112*, 12699-12704.

S33. VandenBeldt K.J., et al. (2006) Kinetochores use a novel mechanism for coordinating the dynamics of individual microtubules. Curr. Biol. *16*,1217–1223.

S34. Chen, W. and Zhang, D. (2004). Kinetochore fibre dynamics outside the context of the spindle during anaphase. Nat. Cell Biol. *6*, 227-231.

S35. Nicklas R.B. (1983). Measurements of the force produced by the mitotic spindle in anaphase. J. Cell Biol. *97*, 542–548.

S36. Risteski, P., Božan, D., Jagrić, M., Bosilj, A., Pavin, N., and Tolić, I.M. (2022). Length-dependent poleward flux of sister kinetochore fibers promotes chromosome alignment. Cell Rep. *40*, 111169.

S37. Dumont S., Salmon E.D., Mitchison T.J. (2012). Deformations within moving kinetochores reveal different sites of active and passive force generation. Science 337, 355-358.
